# Functional Characterization of an Aldol Condensation Synthase PheG for the Formation of Hispidin from *Phellinus Igniarius*

**DOI:** 10.1002/advs.202413192

**Published:** 2025-01-28

**Authors:** Wanting Zhang, Ruliang Zheng, Weiling Geng, Xinyuan Wu, Xiaojuan Gao, Li Zhou, Zhenyu An, Cheng Liu, Zhijun Song, Hongyan Ji, Hao Yang, Xiuli Wu

**Affiliations:** ^1^ College of Pharmacy Key Laboratory of Protection Development and Utilization of Medicinal Resources in Liupanshan Area Ministry of Education Ningxia Medical University Yinchuan 750004 P.R. China; ^2^ Department of Pharmaceutics General Hospital of Ningxia Medical University Yinchuan 750004 P. R. China

**Keywords:** aldol condensase, catalytic mechanisms, enzyme catalysis, heterologous expression, hispidin

## Abstract

Hispidin (**1**) is a polyphenolic compound with a wide range of pharmacological activities that is distributed in both plants and fungi. In addition to natural extraction, hispidin can be obtained by chemical or enzymatic synthesis. In this study, the identification and characterization of an undescribed enzyme, PheG, from *Phellinus igniarius* (*P. igniarius*), which catalyzes the construction of a key C─C bond in the enzymatic synthesis of hispidin are reported. It is demonstrated in vitro that PheG generates hispidin by catalyzing C─C bond formation in the aldol condensation reaction. Based on these results, a plausible pathway for hispidin biosynthesis is proposed by utilizing the primary triacetic acid lactone (TAL, **2**) and 3,4‐dihydroxybenzaldehyde (**3**). The mechanisms for the aldol condensation reaction of PheG are investigated using molecular dynamics (MD) simulations, molecular mechanics/generalized Born surface area (MM/GBSA) binding free energy calculations, density functional theory, and site‐specific mutations. The locations of the key amino acid residues that catalyze the conversion of substrates **2** and **3** to hispidin at the active site of PheG‐1 are identified. This study provides a new method for preparing hispidin with high efficiency and low cost.

## Graphical abstract

Hispidin is a polyphenolic compound with a variety of biological activities. Herein, a new aldol condensase PheG from *Phellinus igniarius* (*P. igniarius*) can catalyze tricetolatone and 3, 4‐dihydroxybenzaldehyde to hispidin. The catalytic mechanism of nucleophilic addition of PheG is determined by MM/GBSA, density functional theory calculation, and site‐directed mutation.

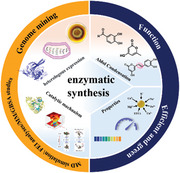

## 1 Introduction

**Figure 1:**
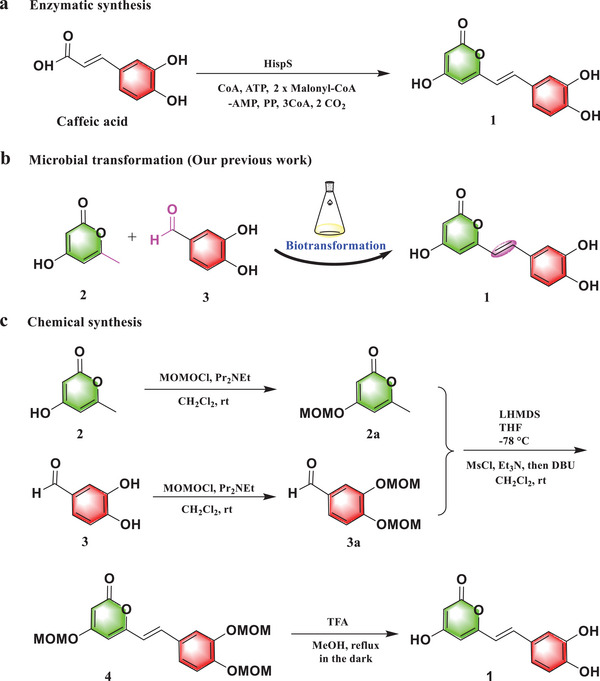
The different synthesis pathways for hispidin. a) The enzymatic synthesis of hispidin, b) the microbial transformation synthesis of hispidin, c) the chemical synthesis of hispidin.

Hispidin, a common polyphenolic compound with antioxidant, anti‐diabetic, antiviral, and anti‐tumor activities in medicinal and edible macrofungi, was first isolated from *Polyporus hispidus* (*P. hispidus*) in 1961.^[^ ^1^ ^]^ Further research has revealed that hispidin derivatives (partial structures shown in Figure S1, Supporting Information) have been found in various related fungal species of the *Hymenochaetaceae* family, including the genera *Inonotus* and *Phellinus*, and have shown a wide variety of biological activities including antioxidant, cytotoxic, anti‐diabetic, and anti‐inflammatory activities.^[^ ^2^, ^3^ ^]^ In view of the unique structure and strong antioxidant activity of hispidin derivatives, these compounds have always attracted the attention of many researchers, and some scholars believe that hispidin derivatives are the primary medicinal components responsible for the antioxidant effects in medicinal fungi.^[^ ^4^ ^]^ Moreover, hispidin has been identified in the luminescent mushroom *Neonothopanus nambi* (*N. nambi*) as a key precursor of fungal luciferin.^[^ ^5^, ^6^ ^]^ However, elucidating the biosynthetic process of hispidin and its derivatives has attracted significant attention. As early as 1973, some scholars proved, using the isotope tracer method, that the biosynthetic pathway of hispidin in *P. hispidus* originated from phenylalanine. In 2018, Kotlobay et al. reported that caffeic acid is converted to hispidin by hispidin synthase (HispS) from *N. nambi* (**Figure** **1**a).^[^ ^7^ ^]^ In our previous work, we identified another hispidin biosynthetic pathway involving **2** and **3** based on the structural characteristics of the extracted compounds and the capture of some intermediate compounds in *P. igniarius*.^[^ ^8^ ^]^ This speculated biosynthetic route was verified by feeding **2** and **3** into a liquid culture of *P. igniarius* (Figure 1b).^[^ ^9^ ^]^ The results showed that the addition of **2** significantly increased the hispidin yield, while the addition of **3** had no effect. Therefore, the low yield of hispidin was due to the limited availability of **2** metabolized by *P. igniarius*, and the key enzyme in *P. igniarius* could catalyze **2** and **3** to produce hispidin. To identify the key enzyme, the whole genome of *P. igniarius* was assembled and analyzed, combined with some protein information inferred by iTRAQ. Seven gene sequences were the focus of attention. However, the mechanism by which hispidin is catalytically generated remains unclear.

In this work, we report, for the first time, that an uncharacterized enzyme, PheG, from *P. igniarius* could catalyze **2** and **3** to produce hispidin by a one‐step enzymatic reaction. Compared with multi‐step chemical synthesis, enzymatic synthesis has obvious advantages, including high efficiency and low pollution, for the production of hispidin (Figure 1c).^[^ ^10^ ^]^

## 2 Results and Discussion

### 2.1 Genome Mining of Supposed Biosynthetic Gene Clusters for 2

**Figure 2:**
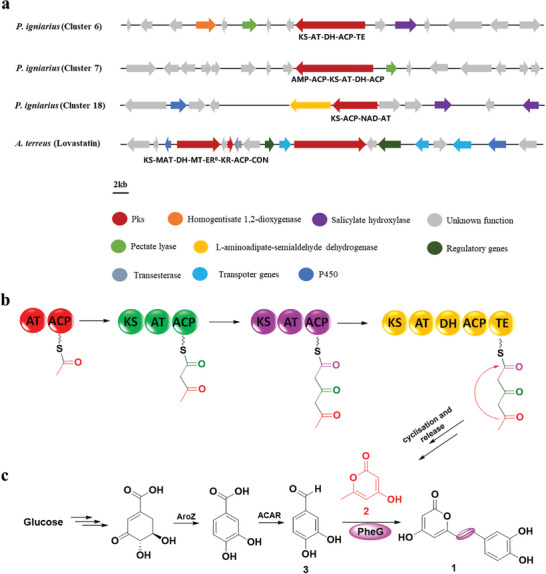
Proposed biosynthetic pathways for hispidin in *P. igniarius*. a) Comparative analysis of biosynthetic genes (putative core genes and functionally characterization synthetic genes) of compound **2**. b) The proposed biosynthetic pathway for **2**. c) The proposed biosynthetic pathway for hispidin.

In our previous study, we found that the addition of **2** increased the production of hispidin (**1**), a metabolite of *P. igniarius*, whereas the addition of **3** had little effect on the production of hispidin.^[^ ^11^ ^]^ Therefore, we speculate that **2** is the rate‐limiting substrate of **1** biosynthesis. To the best of our knowledge, **2** was biosynthesized by PKS,^[^ ^12^, ^13^ ^]^ **3** has been discovered from the *P. igniarius*.^[^ ^14^, ^15^ ^]^ Thus, we speculated that **2** and **3** could be catalyzed by an unknown enzyme PheG to biosynthesize **1** based on previous experimental results and previous studies by other scientists (**Figure** **2**c).^[^ ^8^, ^10^, ^16^, ^17^ ^]^

In order to illustrate the possibility of our hypothesis, we first analyzed the metabolites of *P. igniarius* with HPLC‐MS, and the results showed that the metabolites contained trace amounts of **1** and **3**, which is consistent with previous literature reports, and this result preliminarily supports the possibility of our hypothesis.^[^ ^14^, ^15^ ^]^ Subsequently, 20 biosynthetic gene clusters were identified in the *P. igniarius* genome through anti‐SMASH and local BLAST analyses, including 14 terpenes, one complete NRPS, 3 complete PKS, and one unknown gene cluster (Figure S2, Supporting Information). We compared the three candidate PKS gene clusters with the biosynthetic gene clusters of lovastatin (Figure 2a) and found that candidate gene cluster 6 had the highest similarity (Figure S3, Supporting Information).^[^ ^12^, ^13^ ^]^ These results suggest that cluster 6 may be involved in the biosynthesize of **2** (Figure 2b). Thus, we proposed that possible biosynthetic pathways for hispidin through an unknown function enzyme PheG (Figure 2c).

### 2.2 Mining and Functional Identification of PheGs

**Figure 3:**
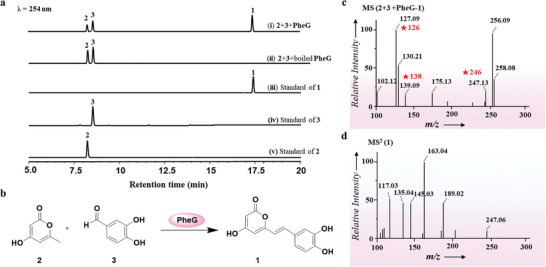
Catalytic function verification of PheG. a) HPLC/DAD of catalytic reaction. b) Chemical reaction process catalyzed by PheG. c) MS of **2** and **3** catalyzed by PheG. d) MS^2^ of compound **1**.

Some scholars have speculated that there is a special biosynthetic pathway for hispidin derivatives in *P. igniarius* different from the previously thought phenylalanine pathway^[^ ^11^ ^]^ and considered TAL was the key intermediate in the synthesis of hispidin derivatives.^[^ ^8^ ^]^ This hypothesis was verified through iTRAQ proteomic analysis in a feeding experiment^[^ ^9^ ^]^ and some proteins that may be involved in hispidin synthesis were predicted. In this study, we successfully cloned and heterologously expressed seven proteins (Figures S4–S17, Table S4, Supporting Information) that may be involved in the synthesis of hispidin. Candidate genes were cloned into pCZN1 vectors, and proteins were expressed in *Escherichia coli*.^[^ ^18^, ^19^ ^]^ The functions of the candidate proteins (0.5 mm) were characterized using **2** and **3** (4.0 mm) as substrates in 60 mL of distilled water incubated at 35 °C for 4 h. The reaction mixtures were analyzed by HPLC/DAD and HPLC/MS/MS. The results showed that the candidate protein PheG (GME6982_g, GenBank accession number: PQ799238) converted **2** and **3** to produce additional new peaks (**Figure** **3**a,b; Figure S18, Supporting Information). The target compound was chromatographed on a Sephadex LH‐20 column (petroleum ether: dichloromethane: methanol, 5:4:1) and identified as hispidin by comparison of NMR and MS spectroscopic data with its reported in the literature (Figure 3c; Figures S19,S20, Supporting Information).^[^ ^1^ ^]^ The mass spectra of the total reaction product (Figure 3c) showed [M+H]^+^ ions at 127.09 (**2**), 139.09 (**3**), and 247.13 Da (hispidin), respectively. The MS/MS spectra of hispidin revealed the main cleavage fragment information, as shown in Figure 3d and Figures S21,S22 (Supporting Information). This result further supports our initial hypothesis (Figure 2c), and it is also suggested that there is a new way to biosynthesis hispidin in *P. igniarius*. Subsequently, we compared PheG sequences to 20 candidate secondary metabolite gene clusters. Interestingly, PheG is not in the 20 candidate gene clusters, but in the sequenced genome.

### 2.3 Properties of PheGs

**Figure 4:**
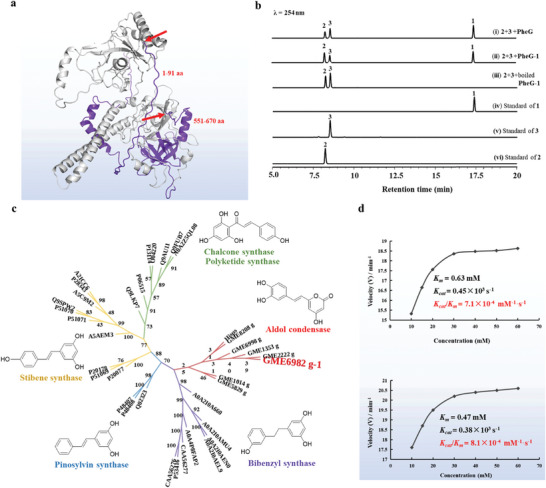
Phylogenetic analysis and functional characterization of PheG and PheG‐1. a) Differences in the 3D structure of PheG and PheG‐1(grey structure). The red arrow shows where the intercept was made. b) HPLC/DAD of catalytic reaction by PheG and PheG‐1, respectively. c) Maximum likelihood tree of PheG‐1 with representative synthase using the neighbor‐joining method by MEGA 11.0. d) Differences in the kinetic parameters of PheG (above) and PheG‐1(below).

Detailed 3D structural analysis showed that the 1–91 aa and 551–670 aa regions of the PheG sequence are intrinsically disordered regions (IDRs) lacking a tertiary structure but possessing a good hydrophobic core.^[^ ^20^, ^21^ ^]^ Therefore, to verify whether the IDR participated in catalytic reactions, the remaining sequence (named GME6982_g‐1, **Figure** **4**a), with the IDRs removed, was heterologously expressed according to the same method used for GME6982_g (Figure S4b, Table S4, Supporting Information). The enzyme induced by IPTG, with a molecular weight of 65.97 kDa (Figures S23–S25, Supporting Information), named PheG‐1, showed the same catalytic function as PheG (Figure 4b). According to the results of BLAST on NCBI, PheG, and PheG‐1 belong to non‐redundant protein sequences and show no significant similarity with existing proteins using UniProtKB and Swiss–Prot (Figures S26,S27, Supporting Information). Sequence alignment (Figure S28, Supporting Information) showed that the protein has high homology with hypothetical fungal proteins. Phylogenetic analysis (Figure 4c) showed that PheG‐1 formed a branch distinct from catalytic enzymes that produce structures similar to hispidin, and their sequences showed low similarity to reported functional proteins (Figures S29,S30, Supporting Information).^[^ ^22^, ^23^ ^]^ The biochemical characteristics of PheG enzymes were investigated using **2** and **3** as substrates.^[^ ^24^, ^25^, ^26^ ^]^ PheG showed its maximum activity at pH 7.0 and 35 °C, while PheG‐1 exhibited its maximum catalytic activity at pH 7.0 and 40 °C. PheG‐1 has a wider tolerance range to temperature and pH than PheG (Figures S31–S34, Supporting Information). Metal ions and EDTA had little influence on the activities of PheG (Table S5, Supporting Information) and PheG‐1 (Table S6, Supporting Information). The kinetic parameters, *Km* and *Kcat*, of PheG were determined using TAL and 2 as substrates at pH 7.0 and 35 °C by standard methods.^[^ ^27^, ^28^ ^]^ The *K_m_* and *K_cat_* values were 0.63 mm and 0.45 × 10^3^ s⁻¹, respectively (Figure 4d). The *K_m_* and *K_cat_* of PheG‐1 were determined as 0.47 mm and 0.38 × 10^3^ s⁻¹ using the same substrates at pH 7.0 and 40 °C. Therefore, since the *K_cat_*/*K_m_* value of PheG‐1 (8.1 × 10⁻⁴ mm⁻¹ s⁻¹) is higher than that of PheG (7.1 × 10⁻⁴ mm⁻¹ s⁻¹), PheG‐1 showed a higher catalytic efficiency (Figure 4d). Although PheG and PheG‐1 exhibited similar catalytic activities, PheG‐1 was superior to PheG in terms of thermostability, pH stability, and affinity. Therefore, further research was conducted on PheG‐1, which has an optimized structure and more stable properties.

### 2.4 Structural Analysis and MD Simulation of PheG‐1

**Figure 5:**
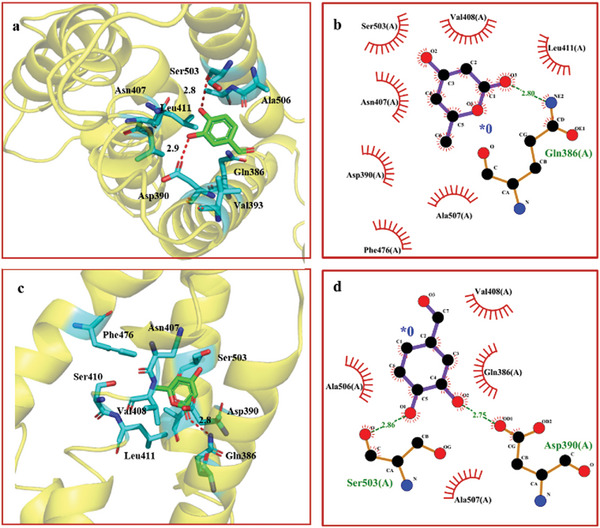
The hypothetical binding mode of **2** or **3** with PheG‐1. a) Compound **2** (green carbon atoms spheres and sticks representation) steric fit in hypothetical binding mode to PheG‐1. b) Molecular interactions of **2** in complex with PheG‐1. c) Compound **3** (green carbon atoms spheres and sticks representation) steric fit in hypothetical binding mode to PheG‐1. d) Molecular interactions of **3** in complex with PheG‐1.

The 3D structure of PheG‐1 could not be determined by X‐ray diffraction because of difficulties in crystallization. Therefore, protein modeling and prediction of the tertiary structure of PheG‐1 were carried out using SWISS‐MODEL^[^ ^29^, ^30^ ^]^ and I‐TASSER (Iterative Threading ASSEmbly Refinement, https://zhanggroup.org//I‐TASSER/).^[^ ^31^, ^32^ ^]^ The best model was determined by comparing the template modeling (TM) scores^[^ ^33^ ^]^ and root mean square deviation values.^[^ ^34^ ^]^ In this study, MOE software was used to conduct molecular docking analysis of PheG‐1 and small molecules. PyMOL (version 2.5.7), Discovery Studio Visualizer (version 2021), and LigPlot+ (version 2.2.8) were used to analyze the details of the interactions between ligands and proteins and to draw 3D diagrams of the interactions (Figure S35, Supporting Information). As shown in **Figure** **5**a,b, small molecule **3** docked into the active site of PheG‐1 and interacted with the surrounding amino acid residues with high affinity (−3.94 kcal mol^−1^). The results showed that **3** interacts with Asp390 (2.8 Å) and Ser503 (2.9 Å) of PheG‐1 through hydrogen bonds. In addition, we found that neighboring amino acid residues around the compound, such as Gln386, Gln389, Leu411, and Ala506, can form van der Waals (VDW) interactions with the small molecule. Similarly, small molecule **2** is also embedded in the active site of PheG‐1 with good affinity (−3.90 kcal mol^−1^) and interacts favorably with the surrounding amino acid residues. As shown in Figure 5c,d, small molecule **2** can form a hydrogen bond (2.8 Å) with Gln386 in the active site of the protein and a cationic interaction (π‐donor) with Val408. In addition, it forms alkyl and π‐alkyl interactions with Leu411, Ala476, and Ala507, and forms VDW interactions with Asp390, Leu409, Ser410, and Val504. In summary, small molecules **2** and **3** can steadily bind to PheG‐1 through hydrogen bonding and VDW interactions, providing a reliable basis for subsequent molecular mechanism research.

### 2.5 Free Energy Landscape (FEL) Analyses and MM/GBSA Studies

**Figure 6:**
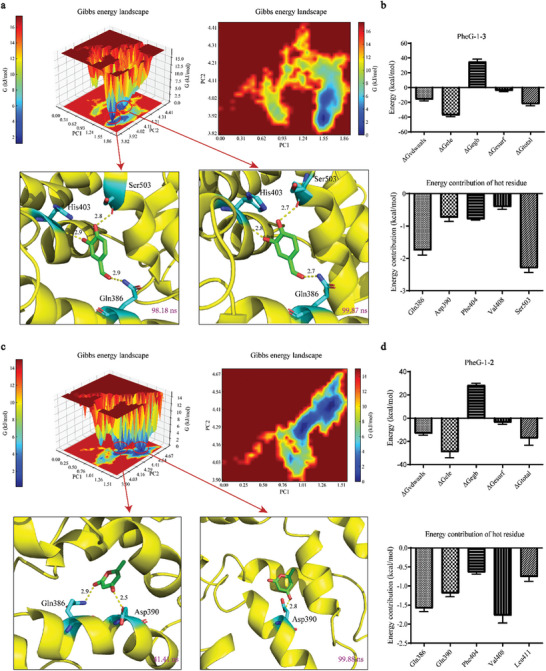
Free energy landscape, binding free energies, and hotspot residue analysis of the tested system. a) 3D and 2D free energy landscapes (upper panel) for the PheG‐1–**3** complex, along with two binding modes (bottom panel) derived from low‐energy wells (blue denotes low free energy regions, and red denotes higher free energy regions). b) Binding free energy components (upper panel) and the energy contribution plot of selected amino acid residues (contributions < −0.3 kcal mol^−1^) for the PheG‐1–**3** complex (bottom panel). c) 3D and 2D free energy landscapes (upper panel) for the PheG‐1–**2** complex, accompanied by two binding modes (bottom panel) derived from low‐energy wells. d) Binding free energy components (upper panel) and the energy contribution plot of selected amino acid residues (contributions < −0.5 kcal mol^−1^) for the PheG‐1–**2** complex (bottom panel).

The MD simulation parameters aligned with our expectations and were suitable for subsequent analyses (Figure S36, Supporting Information). The Gibbs free energy landscape (FEL) visualizes the dynamic evolution and fluctuations of the relative free energy of a system.^[^ ^35^ ^]^ In our analysis, the density of the data points projected onto the plane defined by PC1 and PC2 served as an indicator of the relative frequency of the molecular states. Within the PheG‐1–**3** complex, the relative free energies ranged from 0 to 16.0 kcal mol^−1^. Analysis of the 3D and 2D free‐energy contour plots revealed two distinct energy minima during MD simulations (upper panel of **Figure** **6**a). Representative structures corresponding to these energy minima were captured (Figure 6a, bottom panel). At 98.18 ns, the conformation with a relative free energy of 0 kcal mol^−1^ facilitated the formation of hydrogen bonds between compound **3** and residues Gln386 (2.9 Å), His403 (2.9 Å), and Ser503 (2.8 Å) of PheG‐1. The conformation at 99.87 ns, with a relative free energy of 1.919 kcal mol^−1^, exhibited hydrogen bonding between compound **3** and residues Gln386 (2.8 Å), His403 (2.7 Å), and Ser503 (2.8 Å). These interactions are likely to play a critical role in modulating the functional dynamics of this domain. In the PheG‐1–**2** system, the relative free energy fluctuated between 0 and 14.0 kcal mol^−1^. The FEL revealed several transient energy basins throughout the MD simulation (upper panel of Figure 6c). Two key conformations were identified at the lowest energy minima, corresponding to simulation time points of 41.41 and 99.88 ns, respectively (bottom panel of Figure 6c). At 41.41 ns, the conformation exhibited a relative free energy of 0 kcal mol^−1^, facilitating the formation of hydrogen bonds between compound **2** and residues Gln386 (2.9 Å) and Asp390 (2.5 Å). At 99.88 ns, the conformation had a relative free energy of 0.068 kcal mol^−1^, with TAL forming a hydrogen bond with Asp390 at a distance of 2.8 Å. The MM/GBSA method, a prevalent approach for calculating and predicting the free energy of binding between small molecule ligands and large protein receptors, was used to assess the binding energies of the PheG‐1–**2** and PheG‐1–**3** complexes.^[^ ^36^, ^37^ ^]^ For the PheG‐1–**3** complex (upper panel of Figure 6b), negative values of Δ*G*_vdwaals_ (van der Waals energy), Δ*G*_ele_ (electrostatic energy), and Δ*G*_esurf_ (nonpolar contribution) indicated that van der Waals, electrostatic, and nonpolar solvation energies favorably contribute to the stability of the complex. Conversely, a positive Δ*G*_egb_ (polar contribution) value suggested the presence of repulsive polar interactions, likely due to the interaction between the polar segment of compound **3** and polar residues, resulting in polar–polar repulsion. Despite the repulsive polar interactions, the total binding free energy (Δ*G*_total_) was calculated to be −22.89 ± 0.52 kcal mol^−1^, indicating that the overall complex formation is energetically favorable. Regarding the PheG‐1–**2** system (upper panel of Figure 6d), Δ*G*_vdwaals_, Δ*G*_ele_, and Δ*G*_esurf_ were also negative, with the electrostatic component, Δ*G*_ele_, being the most significant at −28.68 ± 0.80 kcal mol^−1^, highlighting the importance of electrostatic interactions. The total free energy (Δ*G*_total_) was −17.40 ± 0.38 kcal mol^−1^, indicating that the binding process between PheG‐1 and TAL is both spontaneous and thermodynamically favorable. In the PheG‐1–**3** complex (Figure 6b, bottom panel), the residues Gln386, Asp390, Phe404, Val408, and Ser503 contributed energies ranging from −2.40 to −0.31 kcal mol^−1^, exceeding the −0.3 kcal mol^−1^ threshold. In the PheG‐1–**2** complex (Figure 6d, bottom panel), the same residues—Gln386, Asp390, Phe404, Val408, and Ser503—showed energy contributions between −1.92 and −0.62 kcal mol^−1^, all more negative than −0.5 kcal mol^−1^. Notably, in both complexes, the most energetically significant residues were located within the same domain.

### 2.6 Catalytic Mechanisms of PheG‐1

**Figure 7:**
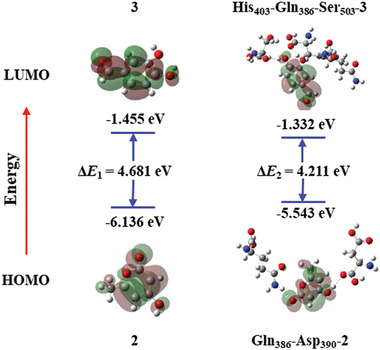
The orbital distribution of highest occupied molecular orbital (HOMO) and lowest unoccupied molecular orbital (LUMO).

Molecular docking combined with molecular dynamics (MD) simulations was employed to elucidate the pivotal amino acid residues within the active site of PheG‐1 that catalyze the conversion of substrates **2** and **3** into hispidin. Therefore, Asp390 and Ser503, which interact with **3**, and Gln386, which interact with **2**, were mutated to Ala.^[^ ^38^ ^]^ The mutant proteins expressed (Figures S37,S38, Table S7, Supporting Information) were used to catalyze the reaction in vitro. The results showed that these proteins no longer had catalytic capacity, which was consistent with the predicted molecular docking results (Figure S39, Supporting Information). To further investigate the mechanism by which PheG‐1 catalyzes the reaction between **2** and **3** to produce hispidin, we used density functional theory and frontier orbital theory. The stable binding conformations of **2**, **3**, and PheG‐1 were determined (Figure 6a,c), and the single‐point energy of each substrate in the reaction system was calculated over 54 iterations. The Δ*G*₁ > 0 (Gibbs free energy variation) for **2** and **3** producing hispidin (Tables S8,S9, Supporting Information) indicated that a catalyst or other conditions were required for the addition reaction,^[^ ^39^ ^]^ which is consistent with our experimental results. The Δ*G*₂ < 0 indicated that the reaction could proceed when **2** and **3** combined with PheG‐1 stably through hydrogen bonds with Ser503, His403, Asp390, and Gln386. Meanwhile, the HOMO–LUMO orbital distribution of the substrates changed significantly when substrates **2** and **3** were bound to amino acid residues (**Figure** **7**).^[^ ^40^ ^]^ The HOMO of **2** shifted toward the methyl group, which increased the nucleophilic activity of the methyl group, whereas the LUMO of **3** shifted toward the aldehyde group (Figure S40, Supporting Information). The nucleophilic properties of the methyl group of **2** and the electrophilic properties of the aldehyde group of **3** increased. The energy gap (Δ*E*) represents the difference in energy between the LUMO and HOMO, which indicates the chemical reactivity of the molecules. The HOMO–LUMO gap (Δ*E*₁) between **2** and **3** was calculated to be 4.681 eV. When PheG‐1 binds to the substrates **2** and **3**, Δ*E*₂ between **2** and **3** decreases to 4.211 eV, allowing the reaction to proceed. Due to the catalysis of PheG‐1, the energy gap between the two substrates decreased significantly. After the nucleophilic addition reaction of the methyl group of **2** attacking the carbonyl carbon of **3**, a molecule of water is spontaneously removed to form a more stable conjugated system, producing the target compound hispidin.

## 3 Conclusion

In summary, we characterized the first aldol condensation synthase, PheG, from *P. igniarius* using an in vitro biochemical assay. It can efficiently catalyze the production of hispidin using **2** and **3** as substrates. Structural analysis, site‐directed mutagenesis, and molecular docking revealed that residues Gln386, Asp390, and Ser503 were key amino acids of PheG in recognizing **2** and **3**, as interpreted by theoretical calculations. Although hispidin synthase (HispS), which catalyzes the formation of hispidin from caffeic acid, has been reported, PheG represents a novel enzyme that can catalyze the formation of hispidin. Thus, our findings uncover a new strategy for generating novel hispidin derivatives using combinatorial biosynthesis and synthetic biology.

## Conflict of Interest

The authors declare no conflict of interest.

## Supporting information



Supporting Information

## Data Availability

The data that support the findings of this study are available from the corresponding author upon reasonable request.
