# Supplementary material for: Functional Characterization of an Aldol Condensation Synthase PheG for the Formation of Hispidin from Phellinus Igniarius
Source: Adv Sci (Weinh). 2025 Jan 28;12(11):2413192. doi: 10.1002/advs.202413192 (PMC11924017; doi:10.1002/advs.202413192)
Supplement: Supplementary file 1 — Supporting Information [file ADVS-12-2413192-s001.docx]

Supporting Information

**Functional Characterization of an Aldol Condensation Synthase PheG for the Formation of Hispidin from Phellinus Igniarius**

Wanting Zhang, Ruliang Zheng, Weiling Geng, Xinyuan Wu, Xiaojuan Gao, Li Zhou, Zhenyu An, Cheng Liu, Zhijun Song,* Hongyan Ji,* Hao Yang,* and Xiuli Wu*

**Table of Contents**

[1. Supplementary Experimental Procedures 4](#_Toc187019145)

[1.1 General materials 4](#_Toc187019146)

[1.2 Plasmid construction 4](#_Toc187019147)

[1.3 Heterologous expression of the candidate genes 5](#_Toc187019148)

[1.4 Denaturation of inclusion body protein 5](#_Toc187019149)

[1.5 The purification of fusion proteins 5](#_Toc187019150)

[1.6 Protein sequence analysis 6](#_Toc187019151)

[1.7 Secondary structure and disorder prediction 6](#_Toc187019152)

[1.8 Functional verification of protein 6](#_Toc187019153)

[1.9 Enzyme characterization 6](#_Toc187019154)

[1.10 *In vitro* activity assay for purified PheGs 6](#_Toc187019155)

[1.11 The kinetic constant of the enzyme 7](#_Toc187019156)

[1.12 Three-dimensional structure prediction of PheG-1 7](#_Toc187019157)

[1.13 Binding pocket prediction of PheG-1 and docking with small molecules 7](#_Toc187019158)

[1.14 Molecular dynamics simulation studies 7](#_Toc187019159)

[1.15 Site-directed mutagenesis 8](#_Toc187019160)

[1.16 DFT calculations 8](#_Toc187019161)

[2. Supplementary Figures 9](#_Toc187019162)

[Figure S1 Structural formulae of hispidin derivatives 10](#_Toc187019163)

[Figure S2 20 secondary metabolite gene clusters of *P. igniarius* 12](#_Toc187019164)

[Figure S3 Comparison of gene cluster 6 and LovB by DNAMAN 8 13](#_Toc187019165)

[Figure S4 The plasmid profile of pCZN1-GME6982_g 14](#_Toc187019166)

[Figure S5 Enzyme digestion and identification 15](#_Toc187019167)

[Figure S6 The plasmid profile of pCZN1-GME1014_g 16](#_Toc187019168)

[Figure S7 Enzyme digestion and identification of pCZN1-GME1014_g 17](#_Toc187019169)

[Figure S8 The plasmid profile of pCZN1-GME8208_g 18](#_Toc187019170)

[Figure S9 Enzyme digestion and identification of pCZN1-GME8208_g 19](#_Toc187019171)

[Figure S10 The plasmid profile of pCZN1-GME2222_g 20](#_Toc187019172)

[Figure S11 Enzyme digestion and identification of pCZN1-GME2222_g 21](#_Toc187019173)

[Figure S12 The plasmid profile of pCZN1-GME1353_g 22](#_Toc187019174)

[Figure S13 Enzyme digestion and identification of pCZN1-GME1353_g 23](#_Toc187019175)

[Figure S14 The plasmid profile of pCZN1-GME5829_g 24](#_Toc187019176)

[Figure S15 Enzyme digestion and identification of pCZN1-GME5829_g 25](#_Toc187019177)

[Figure S16 The plasmid profile of pCZN1-GME6998_g 26](#_Toc187019178)

[Figure S17 Enzyme digestion and identification of pCZN1-GME6998_g 27](#_Toc187019179)

[Figure S18 HPLC-DAD of catalytic product by PheGs 28](#_Toc187019180)

[Figure S19 ^1^H-NMR spectrum of hispidin recorded at 400 MHz in CH_3_OD 29](#_Toc187019181)

[Figure S20 ^13^C-NMR spectrum of hispidin recorded at 100 MHz in CH_3_OD 30](#_Toc187019182)

[Figure S21 HR-ESI-MS analysis of reaction products of 2 and 3 by PheG-1 31](#_Toc187019183)

[Figure S22 The MS^2^ of compounds 32](#_Toc187019184)

[Figure S23 SDS-PAGE purification of PheG and PheG-1 33](#_Toc187019185)

[Figure S24 SDS-PAGE identification of PheG and PheG-1 34](#_Toc187019186)

[Figure S25 Western Blot identification of PheG and PheG-1 35](#_Toc187019187)

[Figure S26 Partial screenshots of BLAST results of PheG and PheG-1 36](#_Toc187019188)

[Figure S27 A partial screenshots of some PheGs templates obtained by SWISS-MODEL 37](#_Toc187019189)

[Figure S28 The results of sequence alignment (subfamily) of PheG and PheG-1 38](#_Toc187019190)

[Figure S29 The sequence similarity comparison of PheG-1 and reported functional protein 39](#_Toc187019191)

[Figure S30 Maximum likelihood tree of PheG-1 40](#_Toc187019192)

[Figure S31 The optimal temperature and stability of PheG 41](#_Toc187019193)

[Figure S32 The optimal temperature and stability of PheG-1 42](#_Toc187019194)

[Figure S33 The optimal pH and stability of PheG 43](#_Toc187019195)

[Figure S34 The optimal pH and stability of PheG-1 44](#_Toc187019196)

[Figure S35 The three-dimensional structure of PheG-1 enzyme protein 45](#_Toc187019197)

[Figure S36 Analysis of molecular dynamics simulation results 46](#_Toc187019198)

[Figure S37 The electrophoresis picture of mutant plasmid double digestion (*Nde I-Xba I* restriction sites) 47](#_Toc187019199)

[Figure S38 SDS-PAGE of *His*6-MiCGT purified by affinity chromatography 48](#_Toc187019200)

[Figure S39 HPLC-DAD of catalytic product by mutants 49](#_Toc187019201)

[Figure S40 The orbital distribution of highest occupied molecular orbital (HOMO) and lowest unoccupied molecular orbital (LUMO) 50](#_Toc187019202)

[3. Supplementary Tables 51](#_Toc187019203)

[Table S1 Bacterial strains and plasmids used and constructed in this study 51](#_Toc187019204)

[Table S2 Media used in this study 52](#_Toc187019205)

[Table S3 Primers in PCR experiments used in this study 53](#_Toc187019206)

[Table S4 Gene and translated amino acid sequences used in this study 54](#_Toc187019207)

[Table S5 Effects of various metal ions and inhibitory additives on PheG 57](#_Toc187019208)

[Table S6 Effects of various metal ions and inhibitory additives on PheG-1 58](#_Toc187019209)

[Table S7 Primer sequence used for site-directed mutations in this study 59](#_Toc187019210)

[Table S8 Gbiss free energy and front-line orbital energy before and after pheG-1 participates in the catalytic reaction 60](#_Toc187019211)

[Table S9 Absolute values and cartesian coordinates (Å) for optimized structures 61](#_Toc187019212)

[Table S10 Total 29 genes used for the phylogenetic analysis of figure 4c 69](#_Toc187019213)

[Table S11 Total 15 genes used for the phylogenetic analysis of figure S30a 70](#_Toc187019214)

[Table S12 Total 49 genes used for the phylogenetic analysis of figure S30b 71](#_Toc187019215)

[4. References 73](#_Toc187019216)

# 1. Supplementary Experimental Procedures

## 1.1 General materials

Bacterial strains and plasmids used and created during this study are listed in Table S1. *P. igniarius* was propagated on modified Martin medium (MMM, Table S2) solid agar or liquid medium at 28 ℃. When grown in liquid culture, *P. igniarius* was cultured in 250 mL baffled Erlenmeyer flasks containing 150 mL MMM at 28 ℃ on a gyratory shaker at 180 rpm unless otherwise noted. The precursor feeding experiment was performed after *P. igniarius* was cultured for 15 days, the TAL dissolved in double distilled water (ddH_2_O) was added into the fermentation liquor at a final concentration of ~0.1mg/mL. Then, the co-culture was then allowed to grow for another 5 days under the same conditions. 2.0 mL fermentation broth was partitioned by ethyl acetate (EtOAc, 3 ×2.0mL). The EtOAc residue was dissolved in 2mL methanol for HPLC detection. *Escherichia* *coli* strains were cultured in lysogeny broth (LB) at 37 °C^[1]^ and supplemented with antibiotics once applicable. LB agar was prepared with NaCl (10.0 g/L tryptone, 10.0 g/L NaCl, 5.0 g/L yeast extract, 15.0 g/L agar, 1.0 mol/L NaOH). Cloning was performed in *Escherichia coli* Arctic-Express^TM^. The microorganism strains involved this paper were preserved with 25% (v/v) glycerine at -80 °C.

Oligonucleotides used in this study were purchased from Sangon Biotech (Shanghai) Co., Ltd. PCR reactions were taken place in a peqSTAR PCR cycler (Peqlab) using Q5® High-Fidelity 2x Master Mix (New England Biolabs) for applications requiring high fidelity such as sequencing and cloning, for other applications TaKaRa Ex Taq® (Takara Bio) was used. PCR reaction products were purified for subsequent cloning and sequencing by gel extraction using Universal DNA Purification Kit (Tiangen Biotech (Beijing) Co., Ltd). Plasmid constructs were cloned using ClonExpress™II (Vazyme Biotech Co., Ltd). Deoxyribonucleic acid (DNA) Sanger sequencing was performed by Sangon Biotech (Shanghai) Co., Ltd.

1D-NMR spectra were recorded at 400 MHz for ^1^H and 100 MHz for ^13^C, respectively, on Bluker 400 MHz spectrometers in methanol-*d*_4_ with solvent peaks used as references. Chemical shifts (*δ*) are reported in parts per million relatively to the resonance of the residual solvent. Coupling constants (*J*) are reported in Hertz (Hz). The reaction mixture was detected by HPLC-DAD (Agilent 1200, German) at 254 nm packed with COSMOSIL Packed Column 5C_18_-ms-II (4.6 mm×250 mm, 5 μm), and eluted by gradually increased methanol in 0.2% formic acid water from 20% to 80% (V/V) over 25 min with 1 mL/min flow velocity and 10 μL injection volume.

Electrospray ionization (positive ionization) in Ultra Scan mode with a capillary voltage of 3.5 kV and a heated capillary temperature of 325 °C was used for LC-MS analysis. HR-ESI-MS measurements were conducted on a Thermo Fisher Exactive Orbitrap equipped with an electrospray ion source and a Betasil 100-3 C18 column (150 ×2.1 mm). The following elution gradient was used: 5% B in A for 1 min, 5% to 98% B in A for 15 min, 98% B in A for 3 min, solvent A: H_2_O + 0.1% HCOOH, solvent B: acetonitrile, flow rate: 0.2 mL min-1, injection volume: 5 µL. Optical rotation measurements were performed using a 0.5 dm cuvette on a JASCO P-1020 polarimeter at 25 °C (unless otherwise noted).

## 1.2 Plasmid construction

All sequences are in the 5’ to 3’ orientation. Phusion polymerase was used for all PCR reactions. Based on PAS (PCR-based Accurate Synthesis)^[2]^ method, full-length splicing primers were designed (Table S3), and the protective base Synthesis gene GME1014_g (GenBank accession number: PQ799242), GME1353_g (GenBank accession number: PQ799241), GME2222_g (GenBank accession number: PQ799240), GME5829_g (GenBank accession number: PQ799239), GME8208_g (GenBank accession number: PQ799236), GME6998_g (GenBank accession number: PQ799237), GME6982_g (GenBank accession number: PQ799238), and GME6982_g-1 were designed at both ends of the primers, which were inserted between the *Nde I*-*Xba I* sites of the vector pCZN1.^[3]^ The recombinant plasmids were transferred into the TOP10 clone strain. Positive clones were selected for sequencing.

## 1.3 Heterologous expression of the candidate genes

The obtained plasmids pET28b(+)-GME1014_g, pET28b(+)-GME1353_g, pET28b(+)-GME2222_g, pET28b(+)-GME5829_g, pET28b(+)-GME8208_g, pET28b(+)-GME6998_g, pCzn1-GME6982_gp, CZN1-GME6982_g and pCZN1-GME6982_g-1 containing N-terminal His_6_-Tag were transformed into Arctic Express and BL21(DE3) successively. A single colony was inoculated into 5.0 mL LB with 37 °C, 220 rpm for overnight. Then, the 0.3 mL bacteria were inoculated in 30.0 mL LB medium containing 50.0 μg/mL [Ampicillin](C:/Users/DELL/Desktop/Application/8.9.6.0/resultui/html/index.html#/javascript:;) and shaken with the same culture condition until to OD_600_ of 0.6-0.8. After that, 1.0 mL culture was centrifuged at 10000 rpm for 2 min, and the sediment was resuspended 100 μL 1×sample loading buffer for the bacterial precipitation. The remaining cultures were added with isopropyl-*β*-D-thiogalactopyranoside (IPTG) at the final concentration of 0.5 mM to induced the fusion protein and cultured 37 °C, 220 rpm for 4 h.^[4]^ The 1.0 mL culture was centrifuged at 10000 rpm at room temperature for 2 min, then the precipitate was resuspended with 100 μL 1×sample loading buffer for next assay. The residual culture was centrifuged with 10 min for discarding the supernatant, and then suspended with PBS to precipitate. The resuspension was crushed by ultrasonic wave, after that, the supernatant and precipitate are respectively added to the loading buffer. 12% SDS-PAGE assay was performed with a Coomassian bright blue stain.

## 1.4 Denaturation of inclusion body protein

The thallus precipitate was suspended in 20 mL lysate (20 mM Tris-HCl containing 1 mM Phenylmethylsulfonyl fluoride (PMSF) and bacteria protease inhibitor cocktail, pH 8.0), After ultrasonic crushing, they were centrifuged 1000 rpm for 20 min at 4 ℃. The precipitates obtained by centrifugation were washed 3 times with inclusion body washing solution (20 mM Tris, 1 mM EDTA, 2 M Urea, 1 M NaCl, 1% Triton X-100, pH 8.0). The inclusion body was then dissolved in a solution buffer (20 mM Tris, 5 mM DTT, 0.15 M Nacl,8 M Urea, pH 8.0) in proportion and placed overnight at 4 ℃. Centrifugation was performed at 10000 rpm for 15 min at room temperature. The obtained supernatant was dropped into 20 mM Tris-HCl, 0.15 M NaCl, pH 8.0 buffer, gradually multiplied gradient dilution and stirred slowly, and the protein solution was put into dialysis bag in 20 mM TrIS-HCl, 0.15 M NaCl, pH 8.0 solution for dialysis overnight.

## 1.5 The purification of fusion proteins

The supernatant of bacteria disruption was injected into a Ni-IDA Binding-Buffer pre-balanced Ni-IDA Sepharose C1-6B affinity chromatography column (CC) at a flow rate of 0.5 mL/min using a low-pressure chromatography system.^[5]^ The CC was rinsed with the NI-IDA Binding Buffer (20.0 mM Tris-HCl, 10.0 mM imidazole, 0.15 M NaCl, pH 8.0) at 0.5 mL/min until and the OD280 value of the effluent reached the baseline. Ni-IDA Washing-Buffer (20.0 mM Tris-HCl, 30.0 mM imidazole, 0.15 M NaCl, pH 8.0) was rinsed at a flow rate of 1.0 mL/min until the outflow OD280 reached baseline. Ni-IDA Elution-buffer (20.0 mM Tris-HCl, 250.0 mM imidazole, 0.15 M NaCl, pH 8.0) was used to elute the target protein at 1.0 mL/min flow rate. The effluent was dialysised (dialysis bag, 8000-14000D) overnight with PBS using 12% SDS-PAGE analysis.

## 1.6 Protein sequence analysis

The primary amino acid sequences of 7 candidate protein and PheG-1 are shown in Table S4. The protein sequences were compared and analyzed by BLAST on NCBI. At the same time, UniportKB and Swiss-prot were used to search the similarity of the protein sequence. We also use sequence alignment (subfamily) of protein sequences to find proteins with high homology to this protein.

## 1.7 Secondary structure and disorder prediction

The amino acid sequence of was analyzed using the PredictionProtein (PP, https://predictprotein.org). RePROF and ProtBert-BFD-sec predict secondary structure elements, namely Helix, Strand and Other. ^[6-^

^7]^Intrinsically disordered proteins are predicted by Meta-Disorder (MD) from protein sequences. The prediction is based on a system of neural networks that combines the outputs from several original prediction methods (NORSnetD, ISOPRED2, PROFbval and Ucon), with the evolutionary profiles and sequence features that correlate with the protein disorder such as predicted solvent accessibility and protein flexibility. ^[8-9]^

## 1.8 Functional verification of protein

The general reaction mixture including the final concentration 4.0 mmol·L^-1^ of 3,4-dihydroxy benzaldehyde, TAL and Adenosine triphosphate, and 0.5 mmol·L^-1^ of enzyme in 60.0 mL ddH_2_O was incubated at 35 ℃ for 4 h. Same volume of ethyl acetate were added to stop reaction and then partitioned for three times. The ethyl acetate extract was evaporated under reduced pressure and used to detect on HPLC/UV and HPLC-MS. The positive reaction fraction was separated by Sephadex LH-20 (petrol-dichloromethane-methanol, 5:4:1) to afford target product (50.0 μg), which was used for nuclear magnetic data detection to determine the chemical structure.

## 1.9 Enzyme characterization

The standard process for the determination of enzyme characterization was implemented in total reaction volume 100.0 μL containing 4.0 mmol·L^-1^ TAL and 3,4-dihydroxybenzaldehyde, 0.2 mol·L^-1^ phosphate buffer (pH 7.5), suitable amount of enzyme solution and measuring at 365 nm, which is the strong absorption of styrylpyrrole compounds. One unit of enzyme activity corresponds to a reduction of 1.0 μmol of substrate per minute.

## 1.10 *In vitro* activity assay for purified PheGs

The optimum pH was determined by incubating the PheG and PheG-1 at 35 °C in phosphate buffer (3.0–10.0) and the enzyme activity was assayed according the above standard process by adding 10 μL of enzyme solution for 10 min. The optimum reaction temperature of PheG and PheG-1 was determined at different temperatures ranging from 20 to 50 °C in pH 7.0 for reacting 30 min. It’s thermostability was incubated in pH 7.0 at various temperatures (20, 25, 30, 35, 40, 45 and 50 °C) in different time intervals (0–300 min). The sampling for pH and temperature stability was carried at 20 minutes interval.

The effects of metal ions and inhibitory additives were determined by preincubating the enzyme with 5 mM solution of the following K^+^, Na^+^, Cu^2+^, Zn^2+^, Mg^2+^, Ca^2+^ and ethylenediaminetetraacetic acid (EDTA) in pH 7.0 for 20 min to determine the residual activity. The enzyme activity without additives was defined as 100%.

## 1.11 The kinetic constant of the enzyme

Under the optimal enzymatic reaction conditions, using different concentration of TAL and 3,4-dihydroxy benzaldehyde as substrate, the kinetic constants of the enzyme were analyzed and determined. The *K_m_* and *V_max_* values were calculated with Graphpad Prism software (Graphpad, San Diego, CA) according to non-linear regression plot using Michaelis–Menten equation.^[10]^ The *K_cat_* was determined by the equation *K_cat_* = *V_max_*/[E]. All the assays were carried out in triplicate and against a control assay without enzyme.

## 1.12 Three-dimensional structure prediction of PheG-1

SWISS-MODEL is a fully automated server for modeling the three-dimensional (3D) structure of a protein from its amino acid sequence using homology modeling techniques. The SWISS-MODEL (https://swissmodel.expasy.org/) template library^[11]-[12][13]^ (SMTL version 2021-03-17, PDB release 2021-03-12) was searched with BLAST ([Camacho et al.](D:/2021-实验相关材料/蛋白建模/pheG_2021-03-24/report.html#blastp))^[14]^ and HHblits ([Steinegger et al.](D:/2021-实验相关材料/蛋白建模/pheG_2021-03-24/report.html#hhblits))^[15]^ for evolutionary related structures matching the target sequence. Based on the I-TASSER (https://zhanggroup.org//I-TASSER/) (Iterative Threading Assembly Refining) server (Yang et al., 2014), the three-dimensional structure of PheG-1 were re-predicted.^[16]^ The TM (Template Modeling) score and RMSD (Root Mean Square Deviation) value of the protein model predicted by I-TASSER were compare to determine the best model.

## 1.13 Binding pocket prediction of PheG-1 and docking with small molecules

Before docking, MOE program was used to construct the structure of the compounds and optimize the energy based on MMFF94x force field. The protein PheG-1 was conducted for energy minimization by I-TASSER (https://zhanggroup.org//I-TASSER/) (Iterative Threading the Assembly refine) server forecasts and the binding site was identified by the built-in Site Finder function of the software. The dominant conformation of the compound combined with PheG-1 was performed by using the parameters configured by the system itself and output the first 10 results with a high score of London dG. Molecular visualization is a key aspect of modeling research analysis and communication. The software of PyMol (version: 2.5.7), Discovery Studio Visualizer (version: 2021) and Ligplot^+^ (version: v.2.2.8) were analyzed the details of the interaction between ligands and receptors and drew 3D diagrams of interactions.

## 1.14 Molecular dynamics simulation studies

Gromacs2022.3 software was used for molecular dynamics simulation. For small molecule preprocessing, AmberTools22 is used to add GAFF force field to small molecules, while Gaussian 16W is used to hydrogenate small molecules and calculate RESP potential. Potential data will be added to the topology file of molecular dynamics system. The simulation conditions were carried out at static temperature of 300K and atmospheric pressure (1 Bar). Amber99sb-ildn was used as force field, water molecules were used as solvent (Tip3p water model), and the total charge of the simulation system was neutralized by adding an appropriate number of Na^+^ ions. The simulation system adopts the steepest descent method to minimize the energy, and then carries out the isothermal isovolumic ensemble (NVT) equilibrium and isothermal isobaric ensemble (NPT) equilibrium for 100000 steps, respectively, with the coupling constant of 0.1 ps and the duration of 100 ps. Finally, the free molecular dynamics simulation was performed. The process consisted of 5000000 steps, the step length was 2fs, and the total duration was 100ns. After the simulation was completed, the built-in tool of the software was used to analyze the trajectory, and the root-mean-square variance (RMSD) and protein rotation radius of each amino acid trajectory were calculated, combined with the free energy (MM/GBSA), free energy topography and other data.^[17-18]^

## 1.15 Site-directed mutagenesis

Site-directed mutagenesis was performed by overlap extension PCR to replace the candidate amino acids with alanine using the plasmid pCZN1-GME6982_g-1 as the template (*Nde I*-*Xba I* restriction sites). The mutations were confirmed by nucleotide sequencing, and the recombinant plasmid was transformed into *E. coli* BL21 (DE3) competent cells. Saturation mutagenesis at positions Q386, D390 and S503 were performed according to the codon bias in *E. coli* or the nucleotides used in pCZN1-GME6982_g-1. Site-directed mutagenesis was performed using the Q5® Site-Directed Mutagenesis Kit (New England Biolabs, USA) and primers were designed according to their kit requirements. The wild-type plasmid was used as the template and relevant primers were shown in Table S7. Every engineered pCZN1-GME6982_g-1 variant was fused to a hexahistidine tag at the C-terminus to facilitate easy purification via affinity chromatography. The enzyme activity assays of the mutants were performed and analyzed as described above.

## 1.16 DFT calculations

All DFT calculations were conducted utilizing Gaussian 16 software.^[19]^ All molecular structures in this study were thoroughly optimized at the non-localized B3LYP-D3/6-31G (d) (SMD) functional level,^[20]-[21][22][23]^ employing hybrid basis sets. All geometric optimizations in the calculation process have no negative frequency, and single point energy is calculated on the basis of geometric optimization. The Gibbs free energy △G of the reaction system was calculated at 298.15K and 1atm, and the influence of pheG-1 on the reaction was illustrated. Finally, we conducted HOMO/LUMO frontier orbital analysis^[24]^ to explain the mechanism of pheG-1 catalyzing this reaction from the perspective of molecular orbital arrangement.

# 2. Supplementary Figures

## Figure S1 Structural formulae of hispidin derivatives

The reference information in the Figure S1 is as follows.

[1] S. Y. Mo, Y. C. Yang, W. Y. He, J. G. Shi, *Chinese Chem. Lett*. **2003**, *14*, 704-706.

[2] S. Y. Mo, S. J. Wang, G. X. Zhou, Y. C. Yang, Y. Li, X. G. Chen, J. G. Shi, *J. Nat. Prod.* **2004**, *67*, 823-828.

[3] Y. Wang, S. Y. Mo, S. J. Wang, S. Li, Y. C. Yang, J. G. Shi, *Org. Lett*. **2005**, *7*, 1675-1678.

[4] Y. Wang, X. Y. Shang, S. J. Wang, S.Y. Mo, S. Li, Y. C. Yang, F. Ye, J. G. Shi, L. He, *J. Nat. Prod.* **2007**, *70,* 296-299.

[5] Y. Wang, S. J. Wang, S. Y. Mo, S. Li, Y. C. Yang, J. G. Shi, *Org. Lett*. **2005**, *7*, 4733-4736.

[6] J. Y. Cho, Y. J. Kwon, M. J. Sohn, S. J. Seok, W. G. Kim, *Bioorg. Med. Chem. Lett*. **2011**, *21*, 1716-1718.

[7] L. f. Zan, J. C. Qin, Y. M. Zhang, Y. H. Yao, H. Y. Bao, X. Li, *Chem. Pharm. Bull*. **2011**, 5*9*, 770-772.

[8] I. K. Lee, S. J. Seok, W. K. Kim, B. S. Yun, *J. Nat. Prod.* **2006**, *69*, 299-301.

[9] I. K. Lee, Y. S. Kim, Y. W. Jang, J. Y. Jung, B. S. Yun, *Bioorg. Med. Chem. Lett*. **2007**, *17*, 6678-6681.

[10] C. Chepkirui, T. Cheng, J. Matasyoh, C. Decock, M. Stadler, *Phytochem. Lett*. **2018**, *25*, 141-146.

[11] L. W. Tian, Y. J. Feng, T. D. Tran, Y. Shimizu, T. Pfeifer, H. T. Vu, R. J. Quinn, *Bioorg. Med. Chem. Lett*. **2017**, *27*, 4007-4010.

[12] I. K. Lee, B. S. Yun, *Bioorg. Med. Chem. Lett*. **2006**, *16*, 2376–2379.

[13] I. K. Lee, B. S. Yun, *J. Antibiot.* **2011**, *64*, 349-359.

[14] I. K. Lee, J. Y. Jung, S. J. Seok, W. G. Kim, B. S. Yun, *Bioorg. Med. Chem. Lett*. **2006**, *16*, 5621–5624.

[15] I. K. Lee, B. S. Yun, *Bioorg. Med. Chem. Lett*. **2007**, *15*, 3309–3314.

[16] I. K. Lee, G. S. Seo, N. B. Jeon, H. W. Kang, B. S. Yun, *J. Antibiot*. **2009**, *62*, 631–634.

[17] K. Kojima, T. Ohno, M. Inoue, H. Mizukami, A. Nagatsu, *Chem. Pharm. Bull*. **2008**, *56*, 173–175.

[18] H. V. K. Wangun, C. Hertweck, *J. Eur. J. Org. Chem*. **2007**, *20*, 3292–3295.

[19] C. S. Wu, Z. M. Lin, L. N. Wang, D. X. Guo, S. Q. Wang, Y. Q. Liu, H. Q. Yuan, H. X. Lou, *Bioorg. Med. Chem. Lett*. **2011**, *21*, 3261-3267.


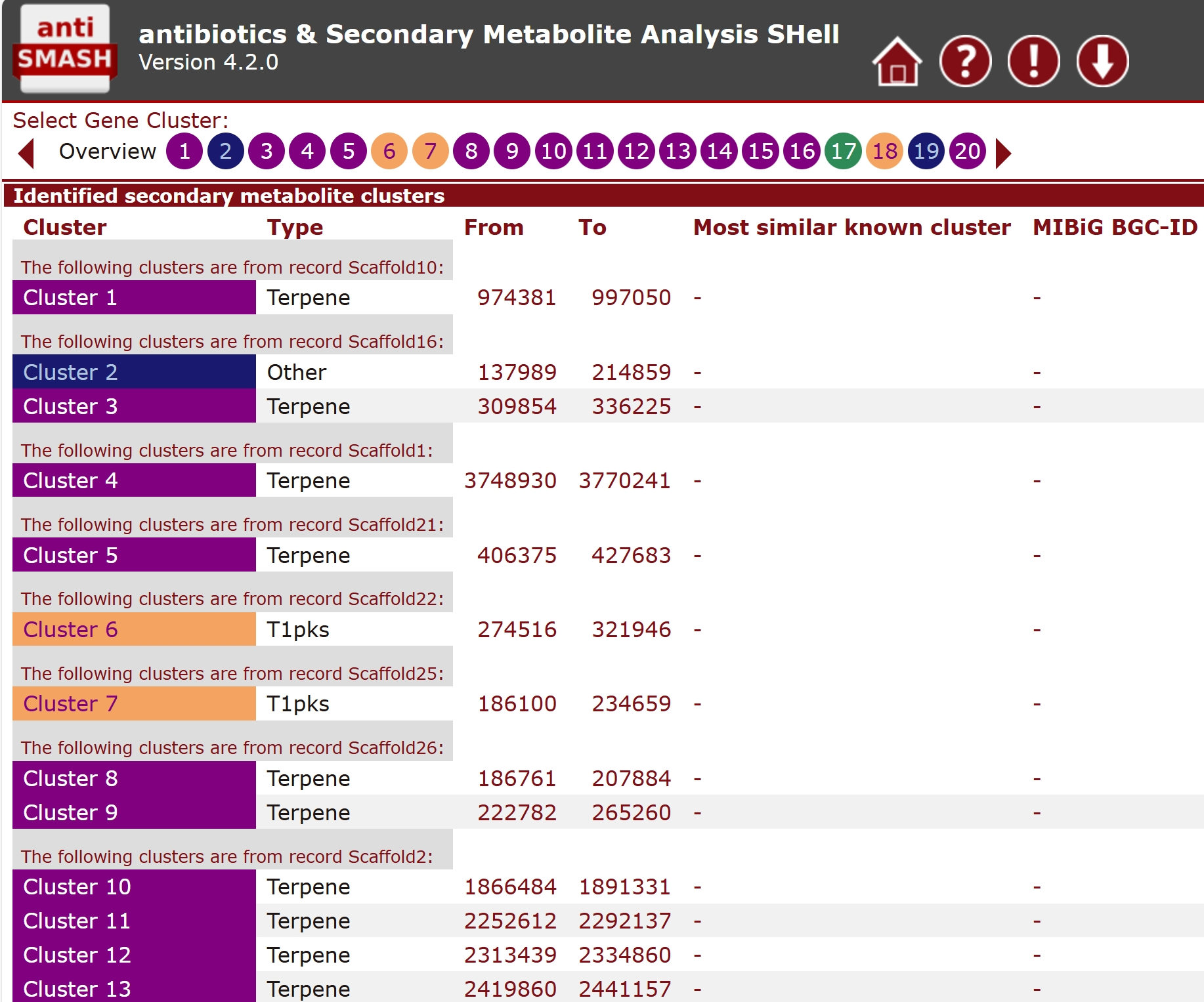


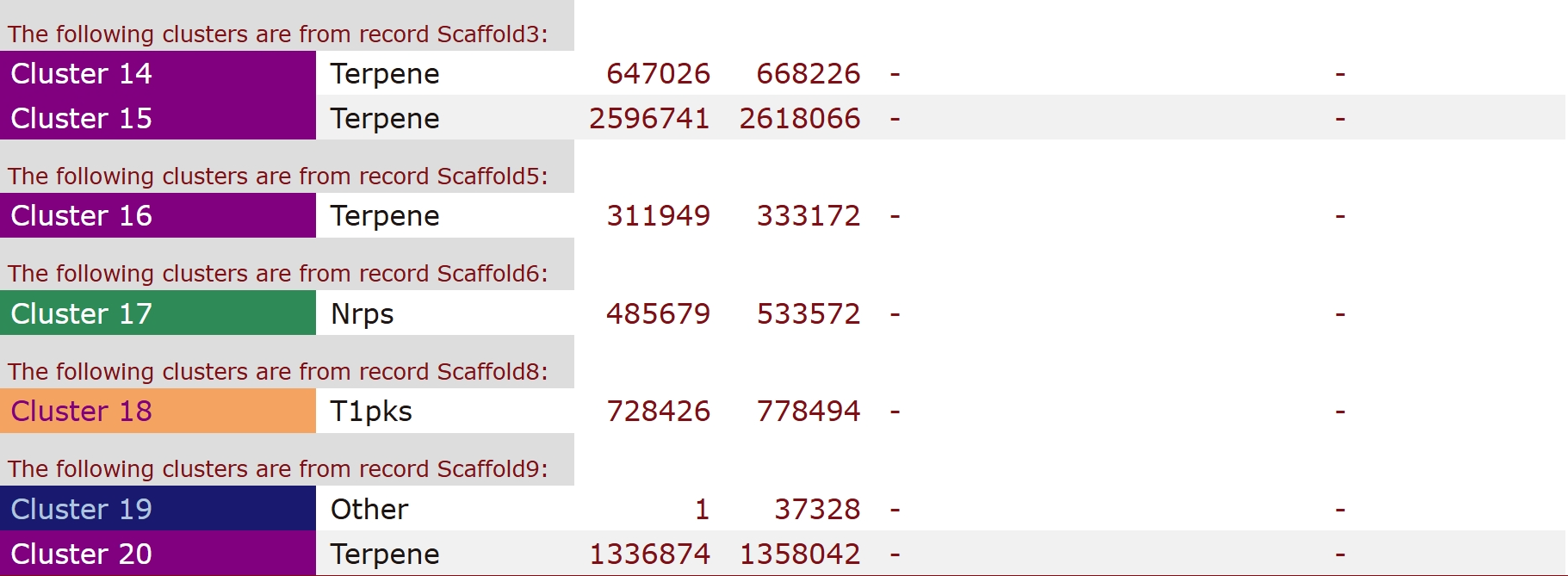


## **Figure S2** 20 secondary metabolite gene clusters of *P. igniarius*


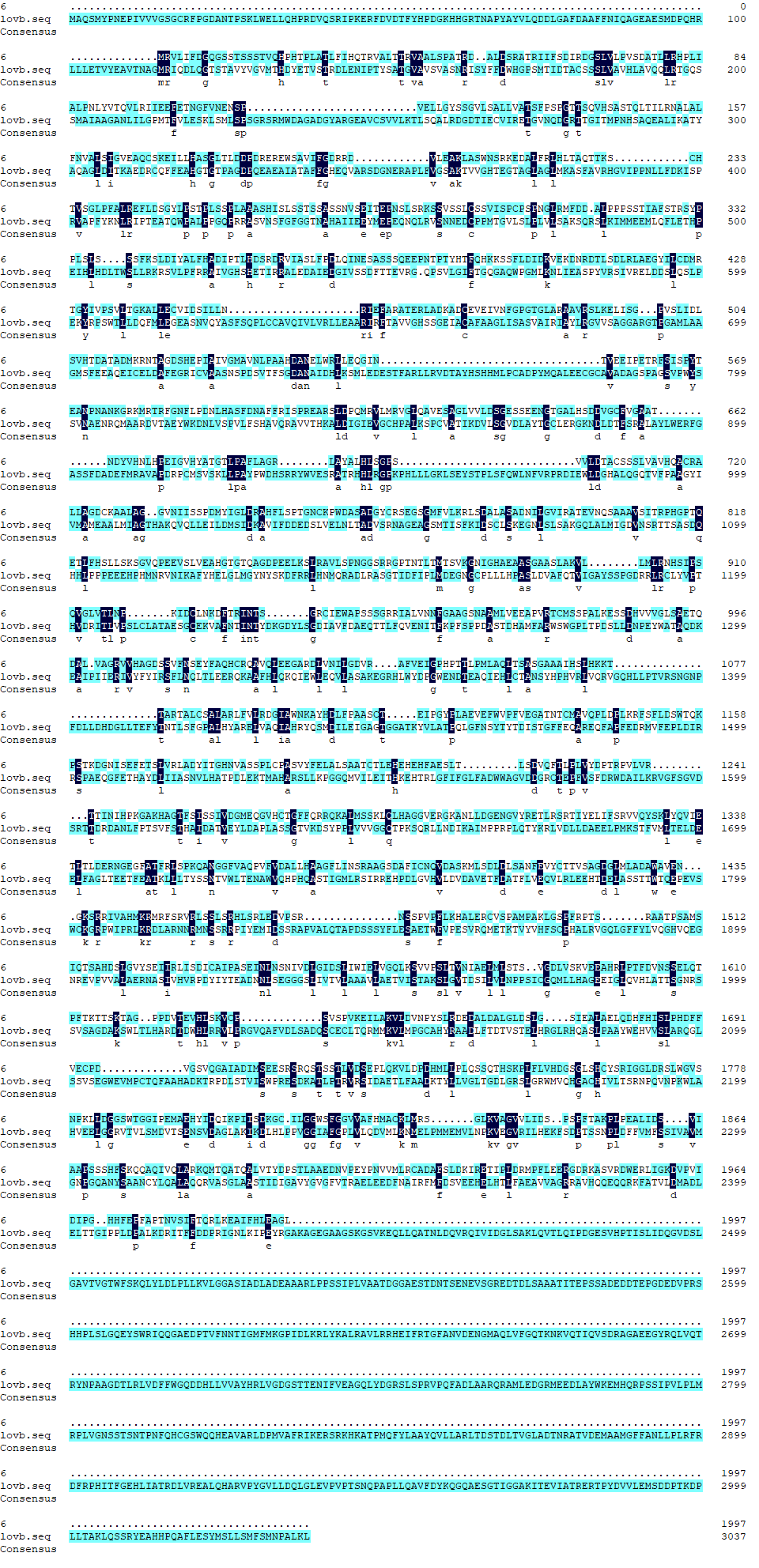


## Figure S3 Comparison of gene cluster 6 and LovB by DNAMAN 8


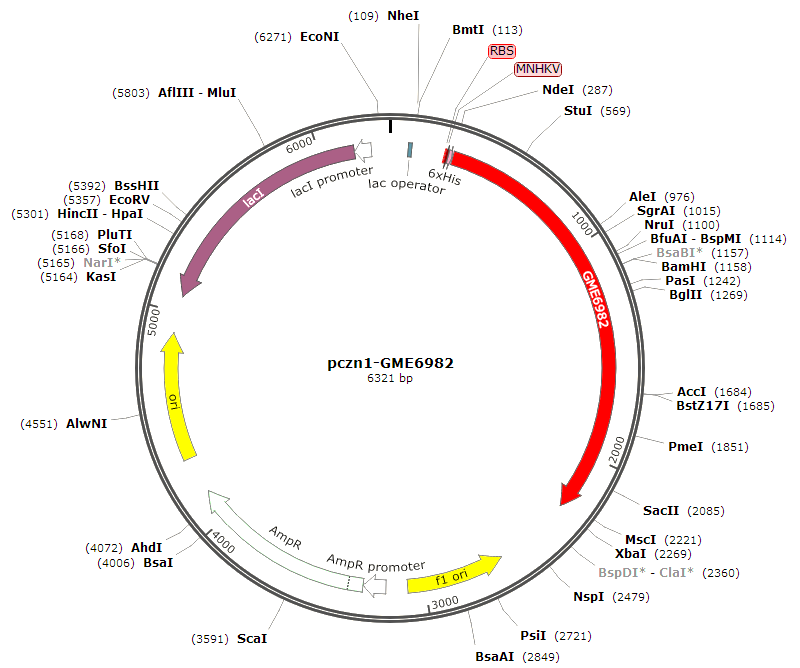


## Figure S4 The plasmid profile of pCZN1-GME6982_g

The target gene was inserted into the pCZN1 vector by homologous recombination through the two restriction sites of NdeI and XbaI.


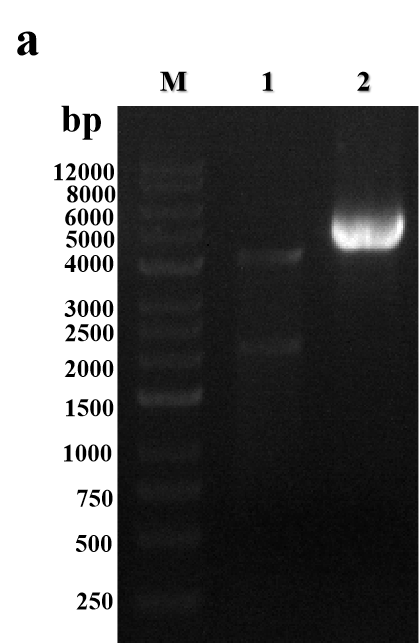

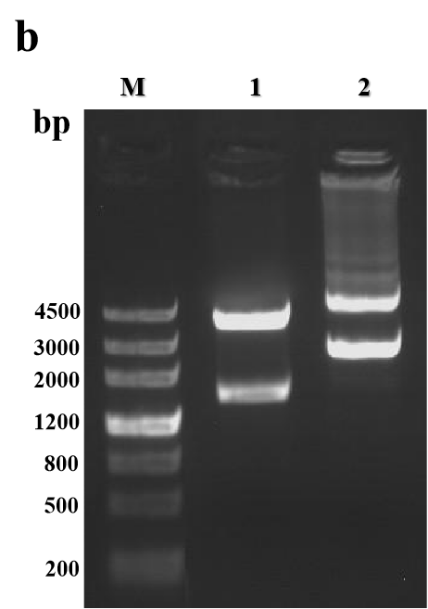


## Figure S5 Enzyme digestion and identification

M = Marker; 1 = Plasmid after double enzyme digestion; 2 = Plasmid before digestion; a) Plasmid profile of pCZN1-GME6982_g. In the swimming lane corresponding to the digested plasmid, there was a corresponding target band between 2000 bp and 2500 bp plasmid (1980 bp); b) Plasmid profile of pCZN1-GME6982_g-1. In the swimming lane corresponding to the digested plasmid, there was a corresponding target band between 1200 bp and 2000 bp plasmid (1755 bp).


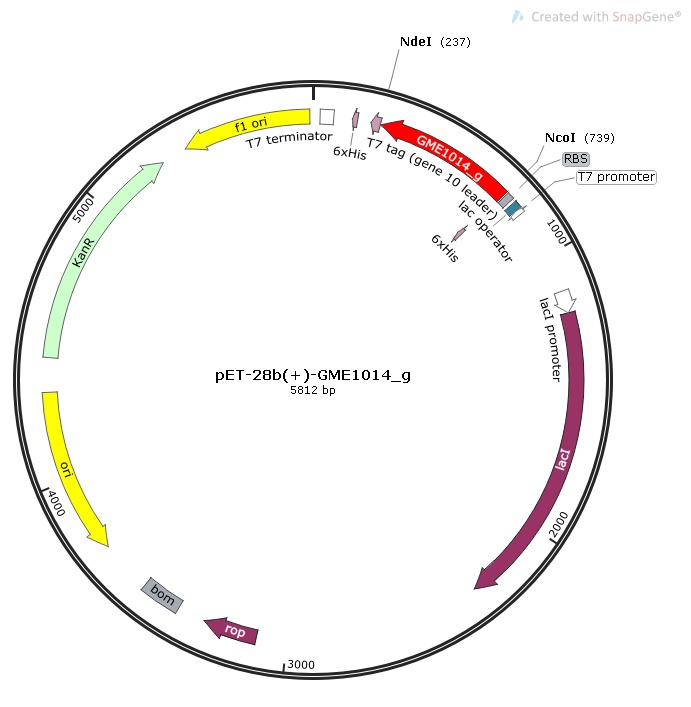


## Figure S6 The plasmid profile of pCZN1-GME1014_g

The target gene was inserted into the pCZN1 vector by homologous recombination through the two restriction sites of NdeI and XbaI.


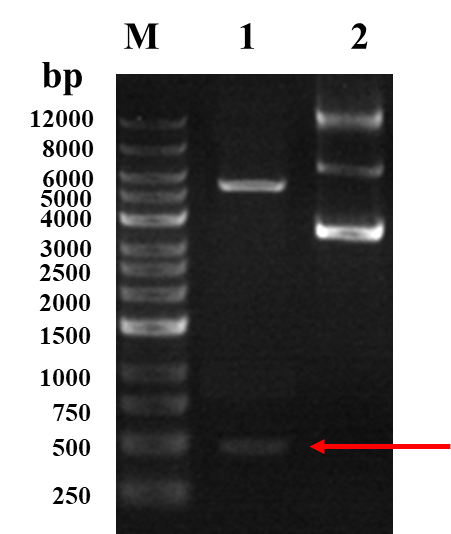


## Figure S7 Enzyme digestion and identification of pCZN1-GME1014_g

M = Marker; 1 = Plasmid after double enzyme digestion; 2 = Plasmid before digestion.


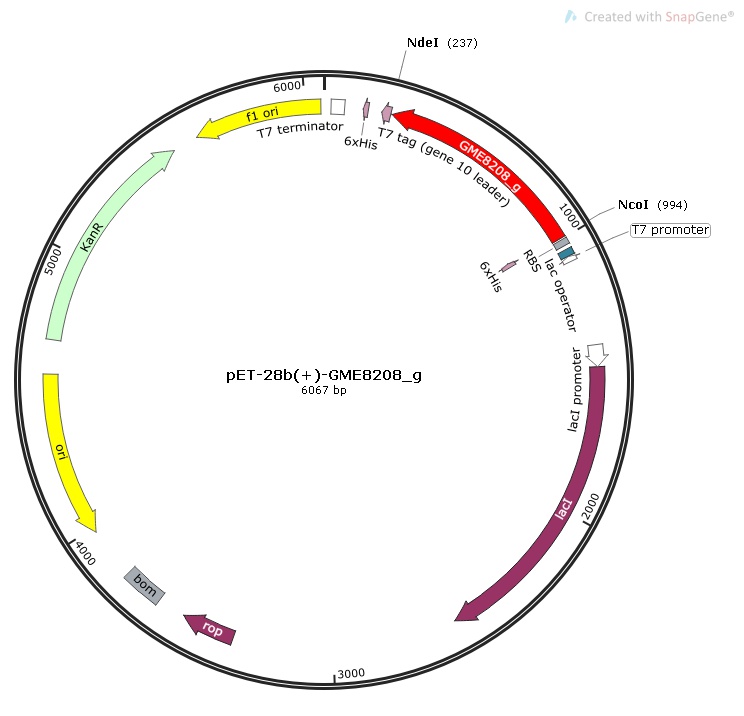


## Figure S8 The plasmid profile of pCZN1-GME8208_g

The target gene was inserted into the pCZN1 vector by homologous recombination through the two restriction sites of NdeI and XbaI.


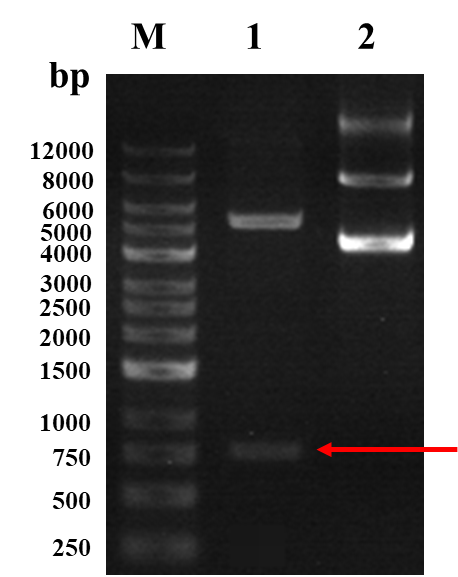


## Figure S9 Enzyme digestion and identification of pCZN1-GME8208_g

M = Marker; 1 = Plasmid after double enzyme digestion; 2 = Plasmid before digestion.


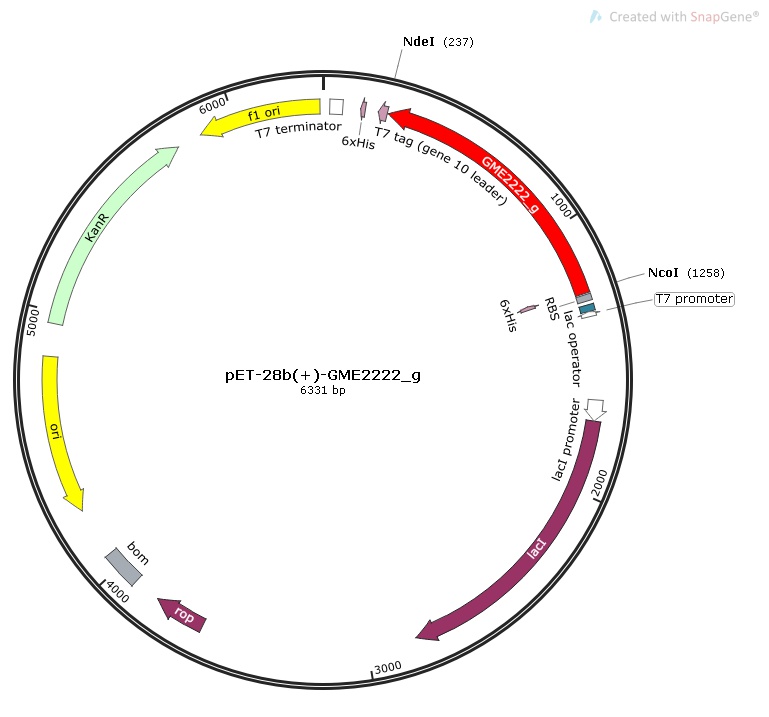


## Figure S10 The plasmid profile of pCZN1-GME2222_g

The target gene was inserted into the pCZN1 vector by homologous recombination through the two restriction sites of NdeI and XbaI.


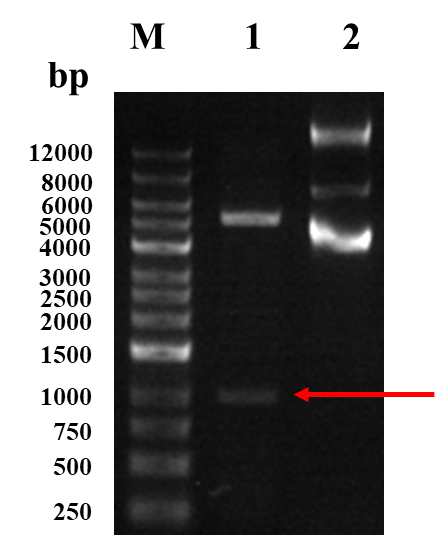


## Figure S11 Enzyme digestion and identification of pCZN1-GME2222_g

M = Marker; 1 = Plasmid after double enzyme digestion; 2 = Plasmid before digestion.


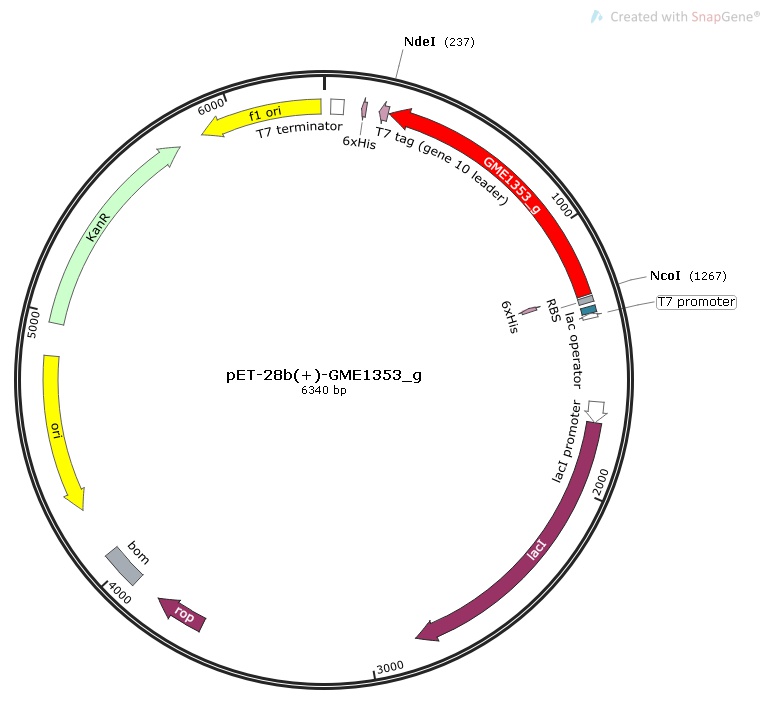


## Figure S12 The plasmid profile of pCZN1-GME1353_g

The target gene was inserted into the pCZN1 vector by homologous recombination through the two restriction sites of NdeI and XbaI.


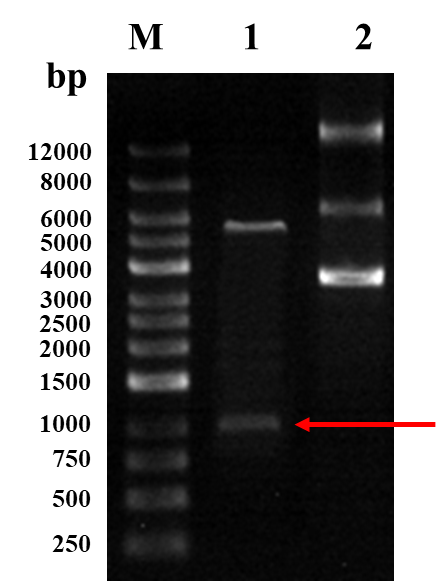


## Figure S13 Enzyme digestion and identification of pCZN1-GME1353_g

M = Marker; 1 = Plasmid after double enzyme digestion; 2 = Plasmid before digestion.


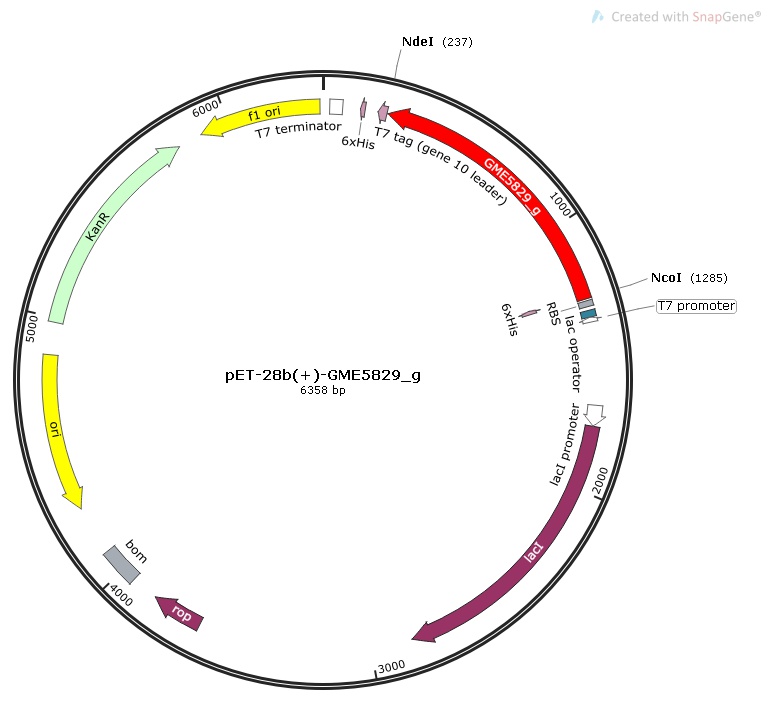


## Figure S14 The plasmid profile of pCZN1-GME5829_g

The target gene was inserted into the pCZN1 vector by homologous recombination through the two restriction sites of NdeI and XbaI.


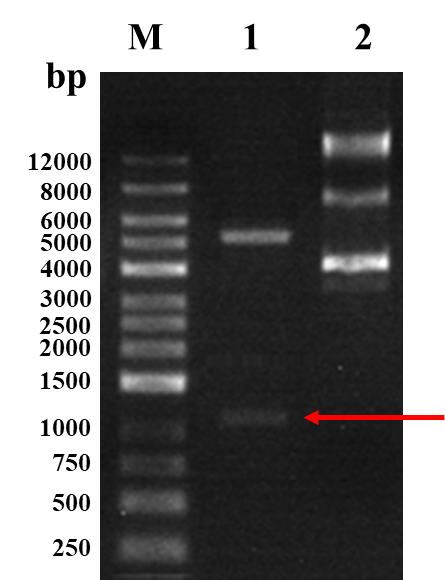


## Figure S15 Enzyme digestion and identification of pCZN1-GME5829_g

M = Marker; 1 = Plasmid after double enzyme digestion; 2 = Plasmid before digestion.


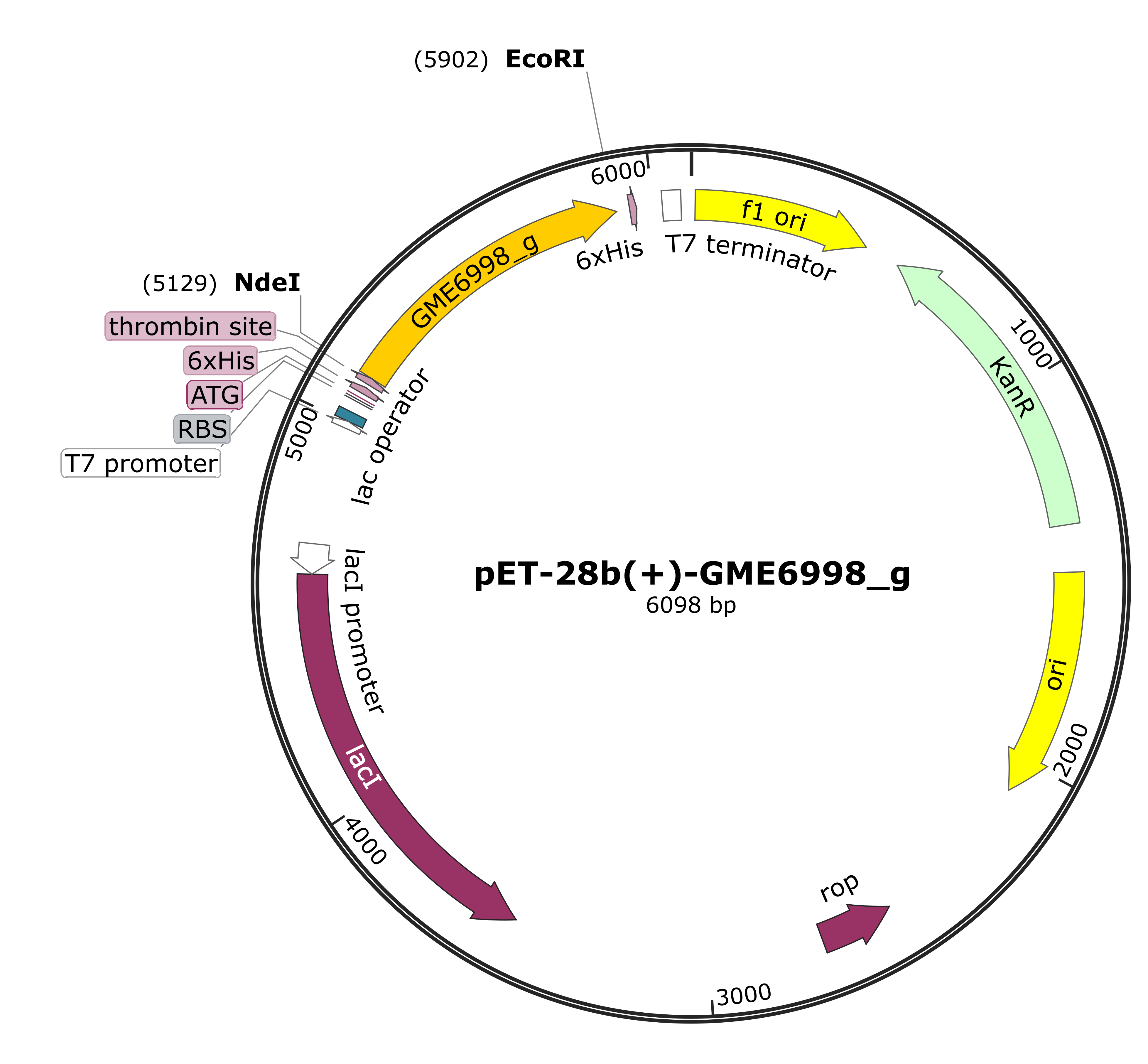


## Figure S16 The plasmid profile of pCZN1-GME6998_g

The target gene was inserted into the pCZN1 vector by homologous recombination through the two restriction sites of NdeI and XbaI.


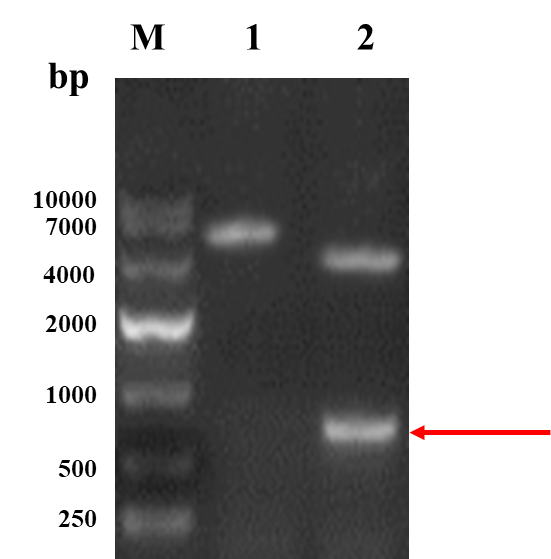


## Figure S17 Enzyme digestion and identification of pCZN1-GME6998_g

M = Marker; 1 = Plasmid double enzyme before digestion; 2 = Plasmid after digestion.


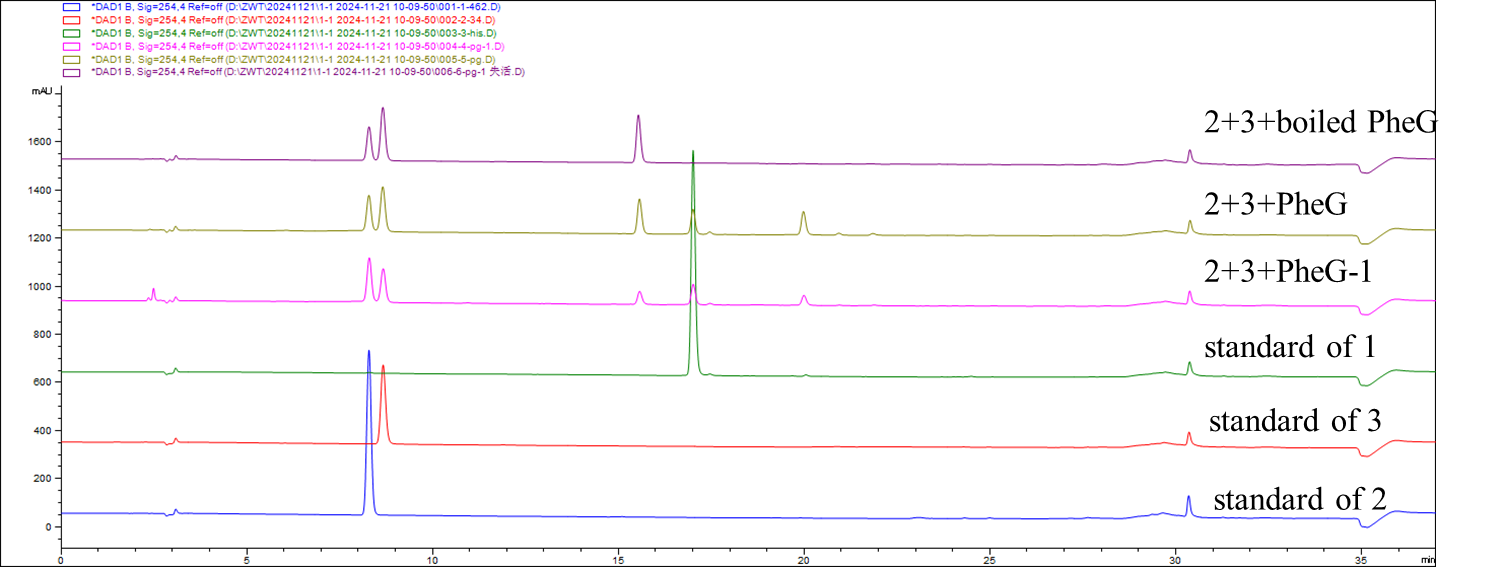


## Figure S18 HPLC-DAD of catalytic product by PheGs


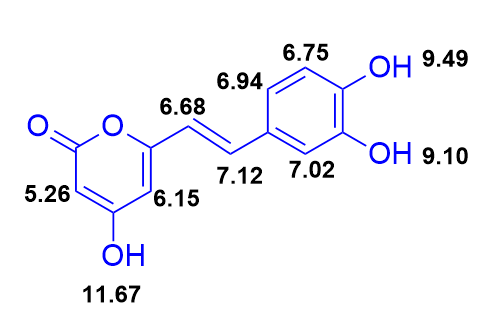

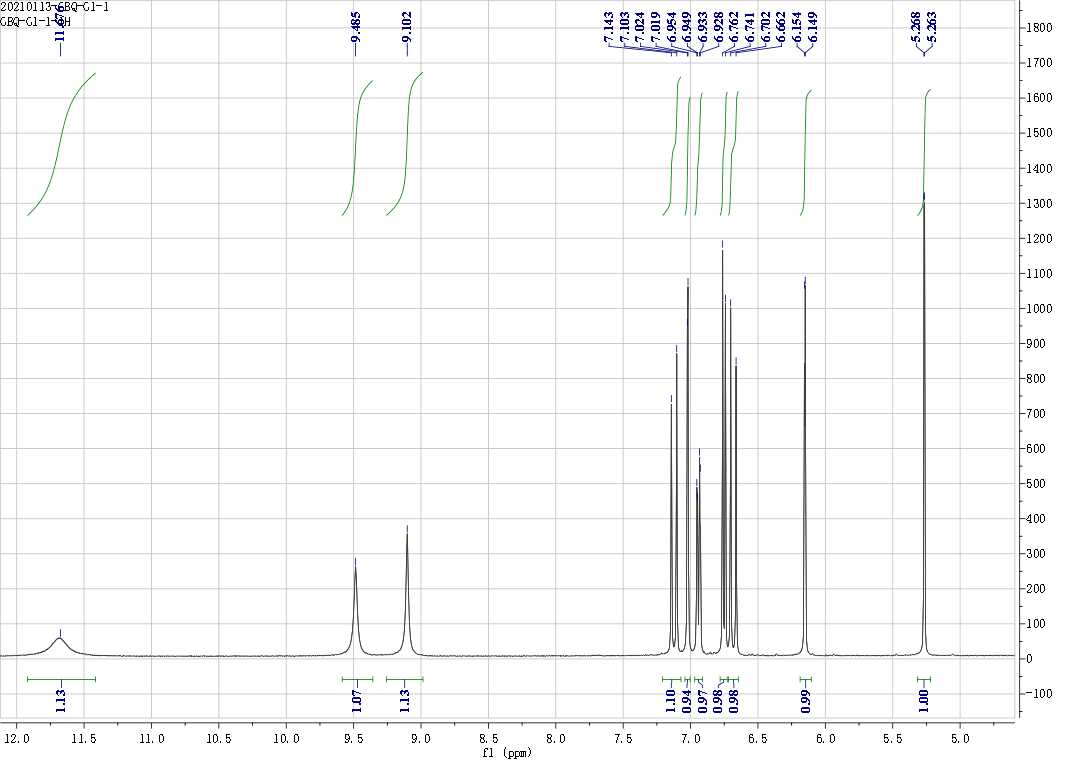


## Figure S19 ^1^H-NMR spectrum of hispidin recorded at 400 MHz in CH_3_OD


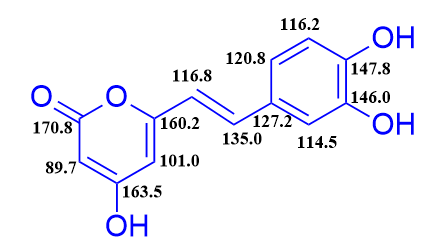

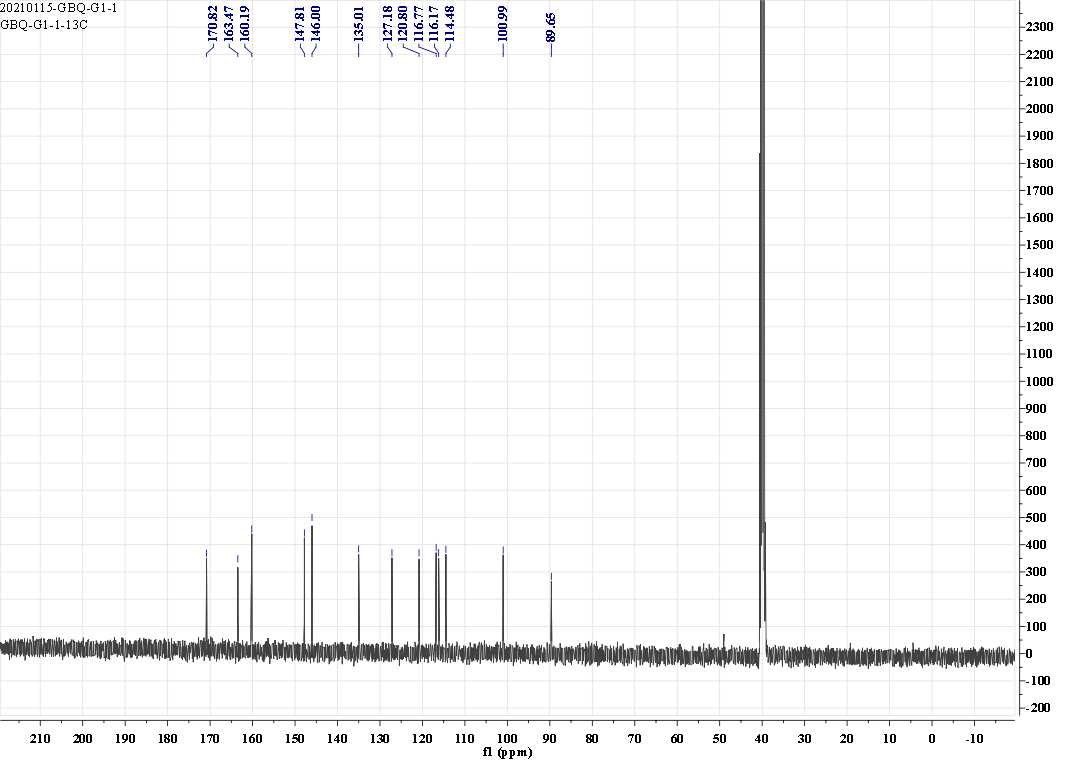


## Figure S20 ^13^C-NMR spectrum of hispidin recorded at 100 MHz in CH_3_OD


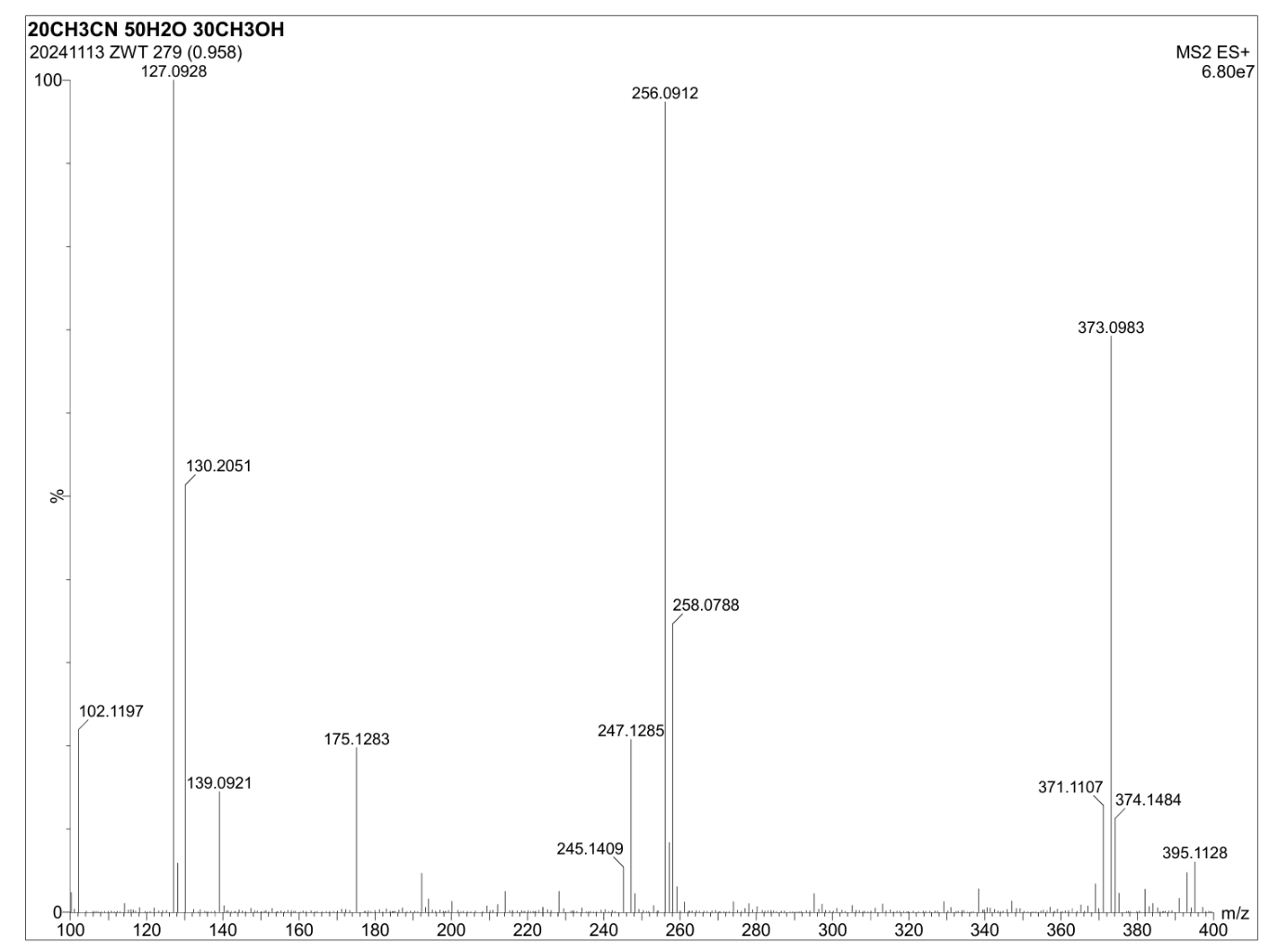


## Figure S21 HR-ESI-MS analysis of reaction products of 2 and 3 by PheG-1

a

b

**c
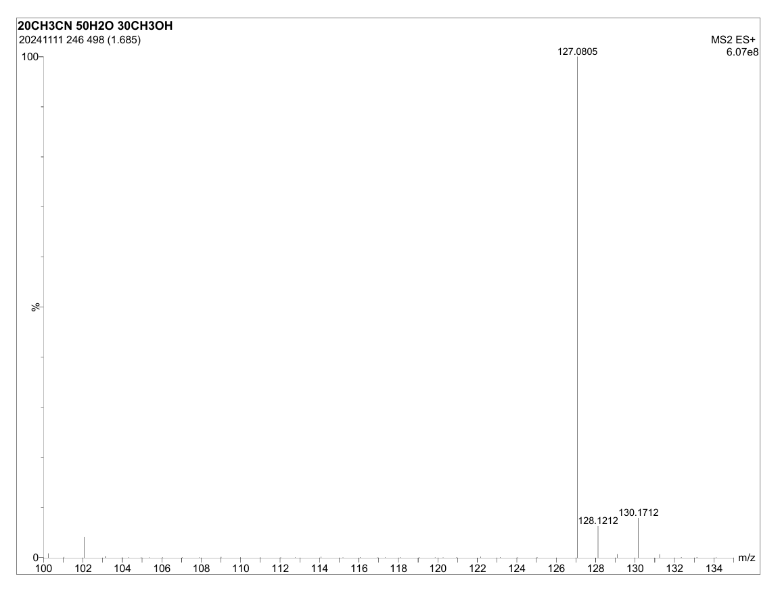
**

**d
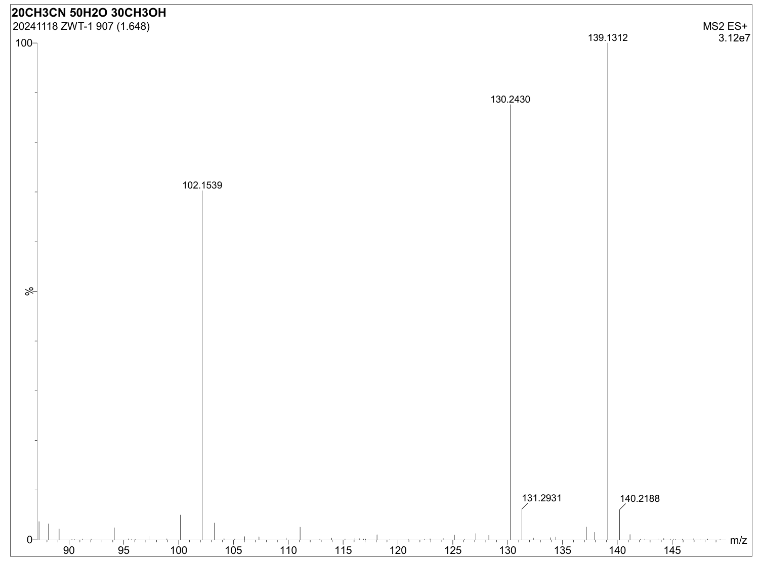
**

## Figure S22 The MS^2^ of compounds

a) MS^2^ of compound **1**. b) Proposed fragment process of compound **1**. c) MS^2^ of compound **2**. d) MS^2^ of compound **3**.


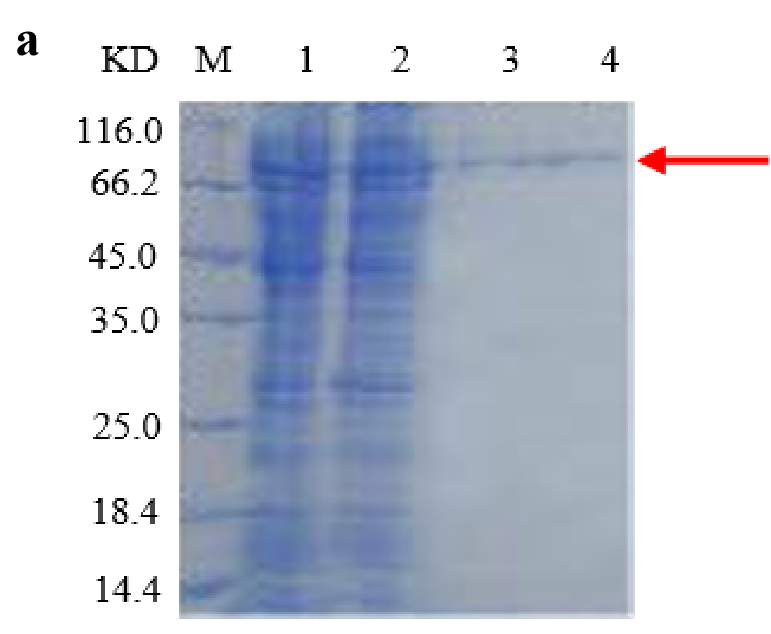

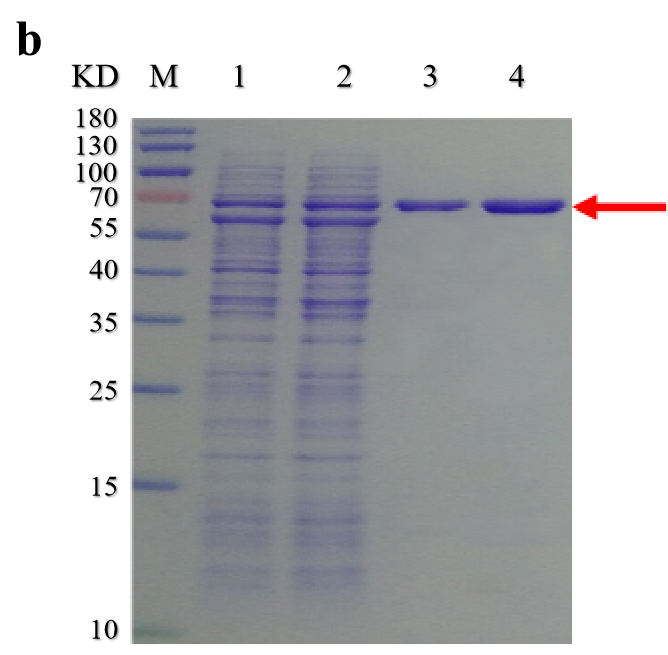


## Figure S23 SDS-PAGE purification of PheG and PheG-1

M = Marker; 1 = Dispose of samples after crushing; 2 = Outflow; 3-4 = Elution; a) SDS-PAGE purification of PheG; b) SDS-PAGE purification of PheG-1. The location indicated by the red arrow is the target protein.


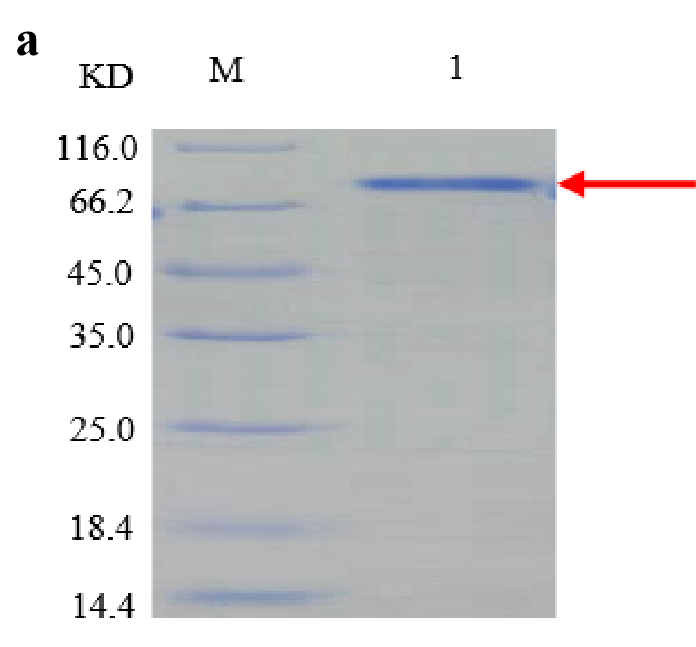

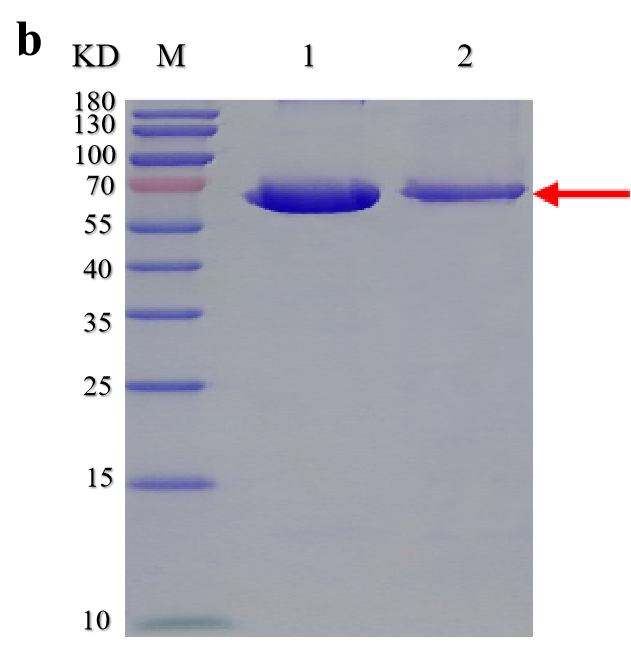


## Figure S24 SDS-PAGE identification of PheG and PheG-1

a) SDS-PAGE identification of PheG. M = Marker; 1 = Purified sample; b) SDS-PAGE identification of PheG-1. M = Marker; 1 = 0.5 mg/mL BSA; 2 = Purified sample. The location indicated by the red arrow is the target protein.

**
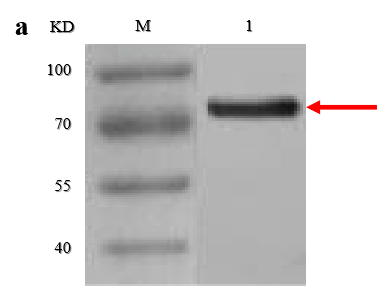

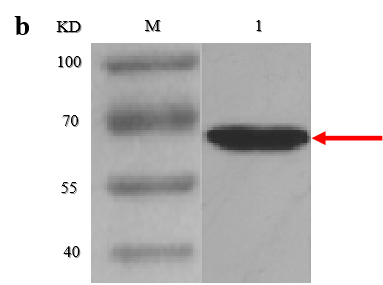
**

## Figure S25 Western Blot identification of PheG and PheG-1

M = Marker; 1 = Purified sample. a) Western Blot identification of PheG; b) Western Blot identification of PheG-1. The location indicated by the red arrow is the target protein


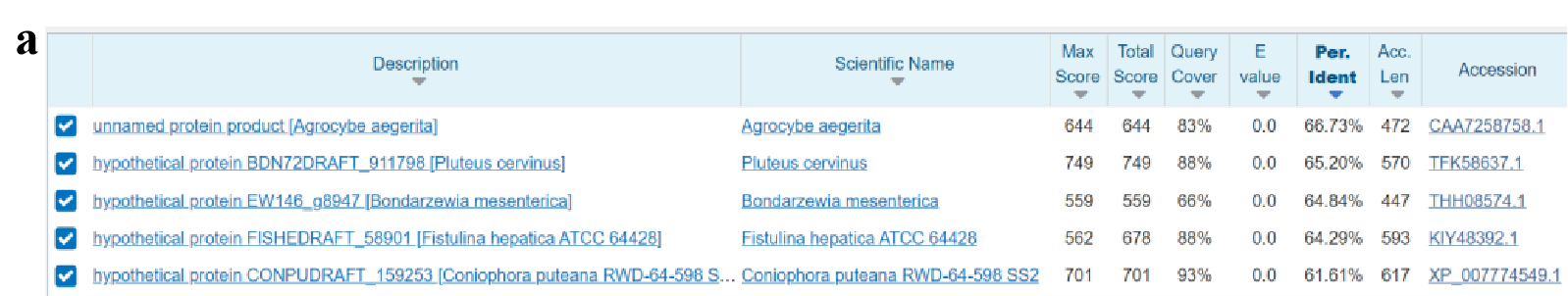


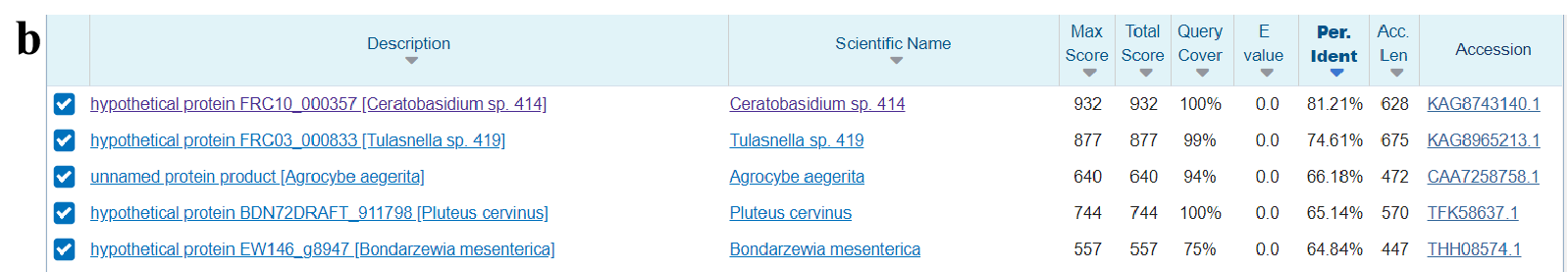


## Figure S26 Partial screenshots of BLAST results of PheG and PheG-1

a) BLAST results of PheG; b) BLAST results of PheG-1.


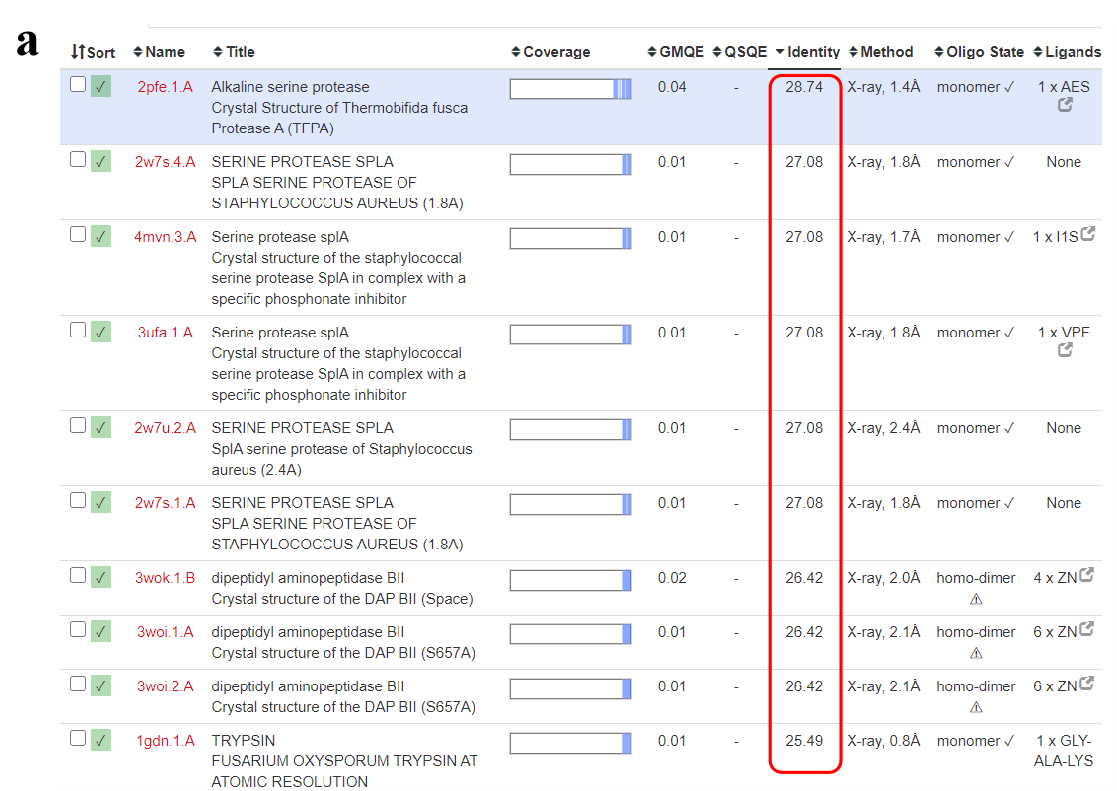

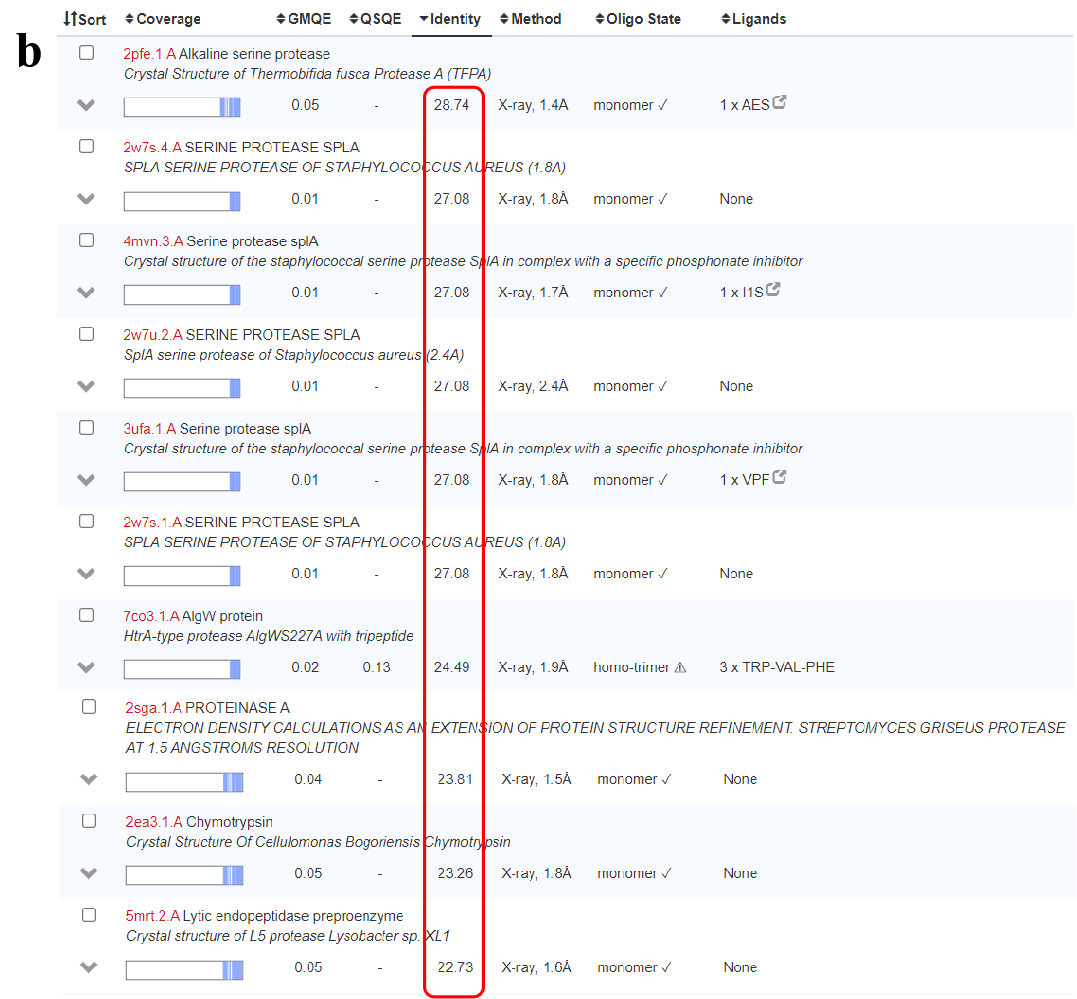


## Figure S27 A partial screenshots of some PheGs templates obtained by SWISS-MODEL

a) PheG; b) PheG-1; The numbers of the red box indicate the homology between each template and the protein sequence.

**a**


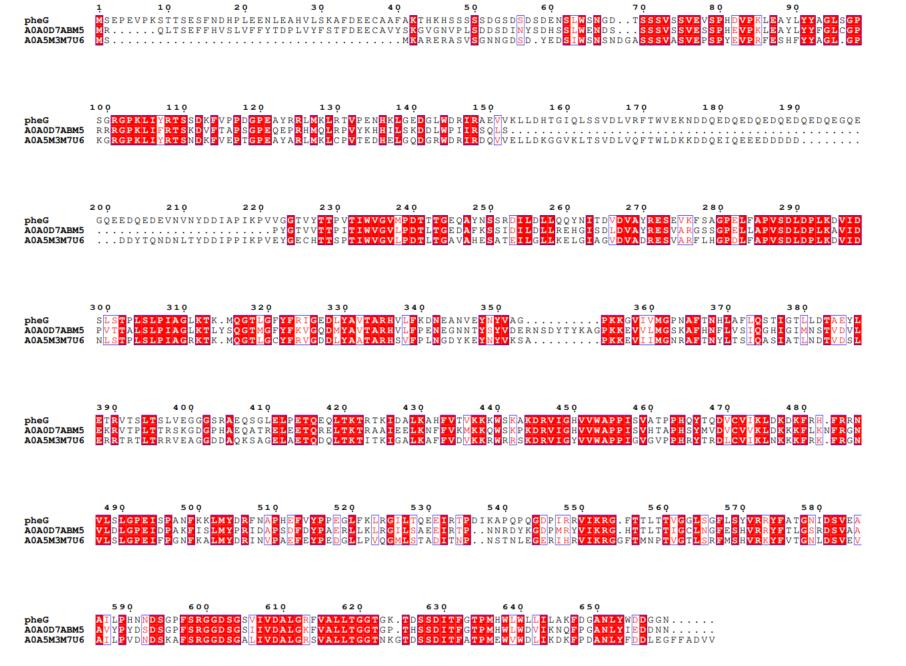


**b**
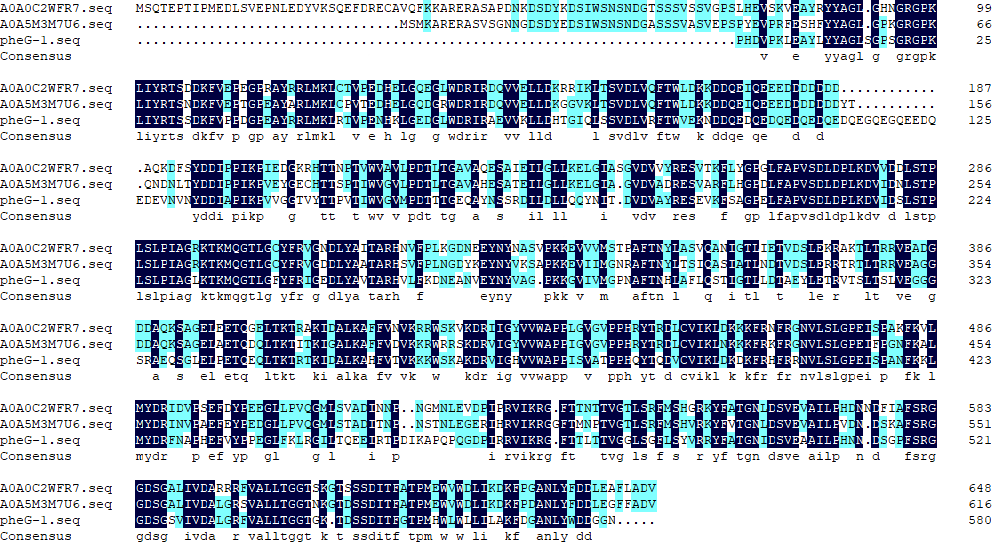


## Figure S28 The results of sequence alignment (subfamily) of PheG and PheG-1

a) Alignment of amino-acid sequences of PheG. The red section represents amino acid similarity; b) Alignment of amino-acid sequences of PheG-1. The gray section represents amino acid similarity.


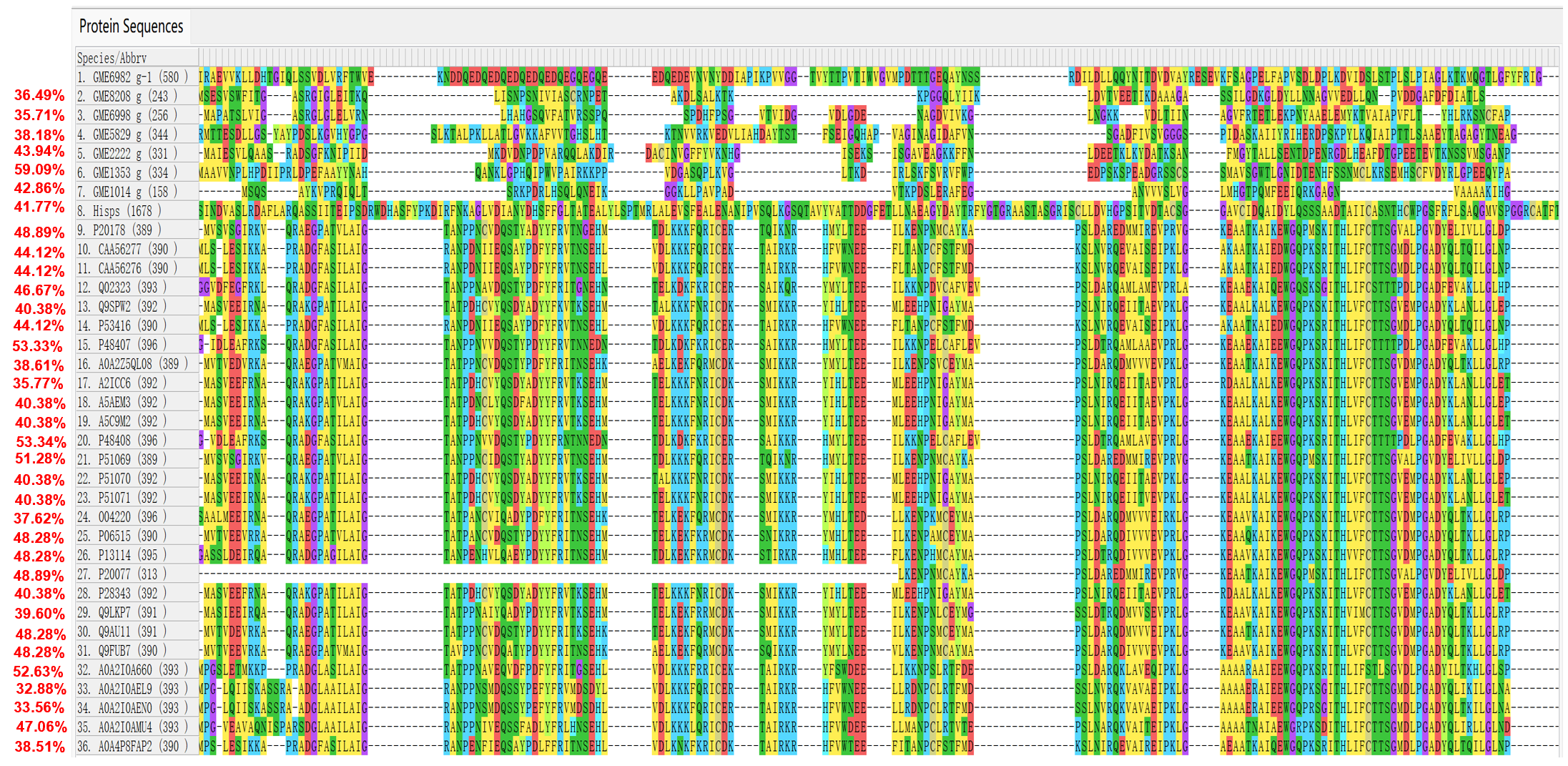


## Figure S29 The sequence similarity comparison of PheG-1 and reported functional protein

a
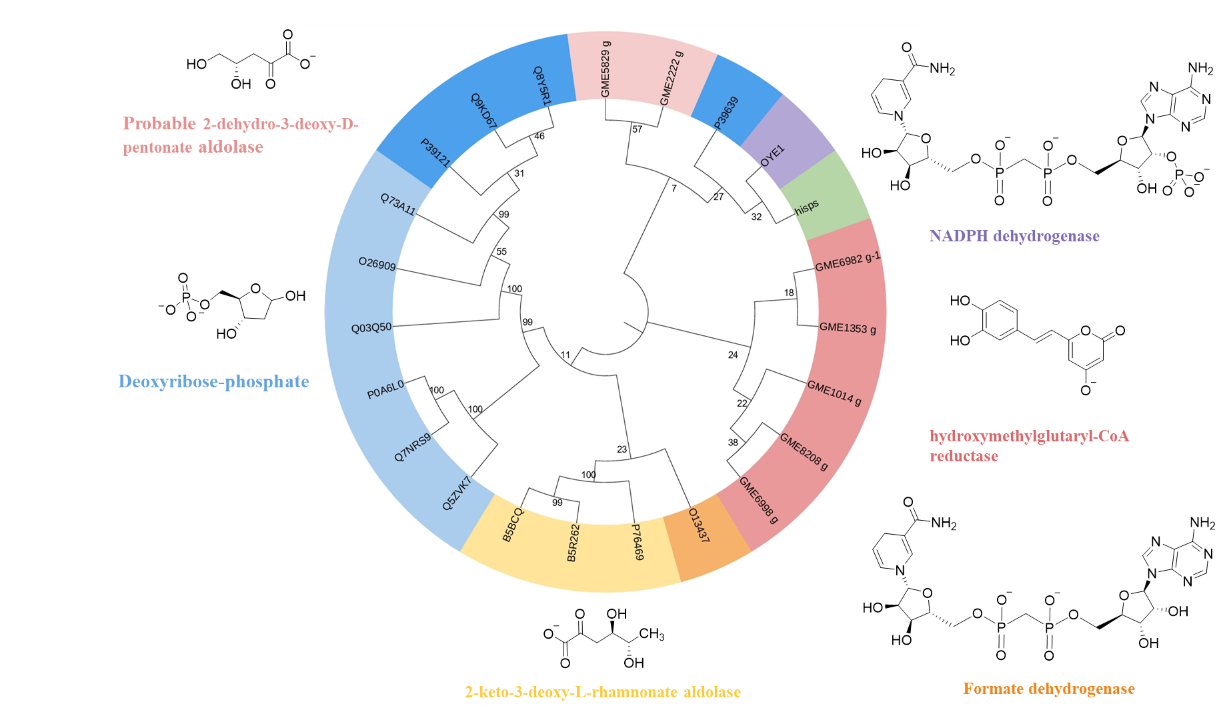


**b
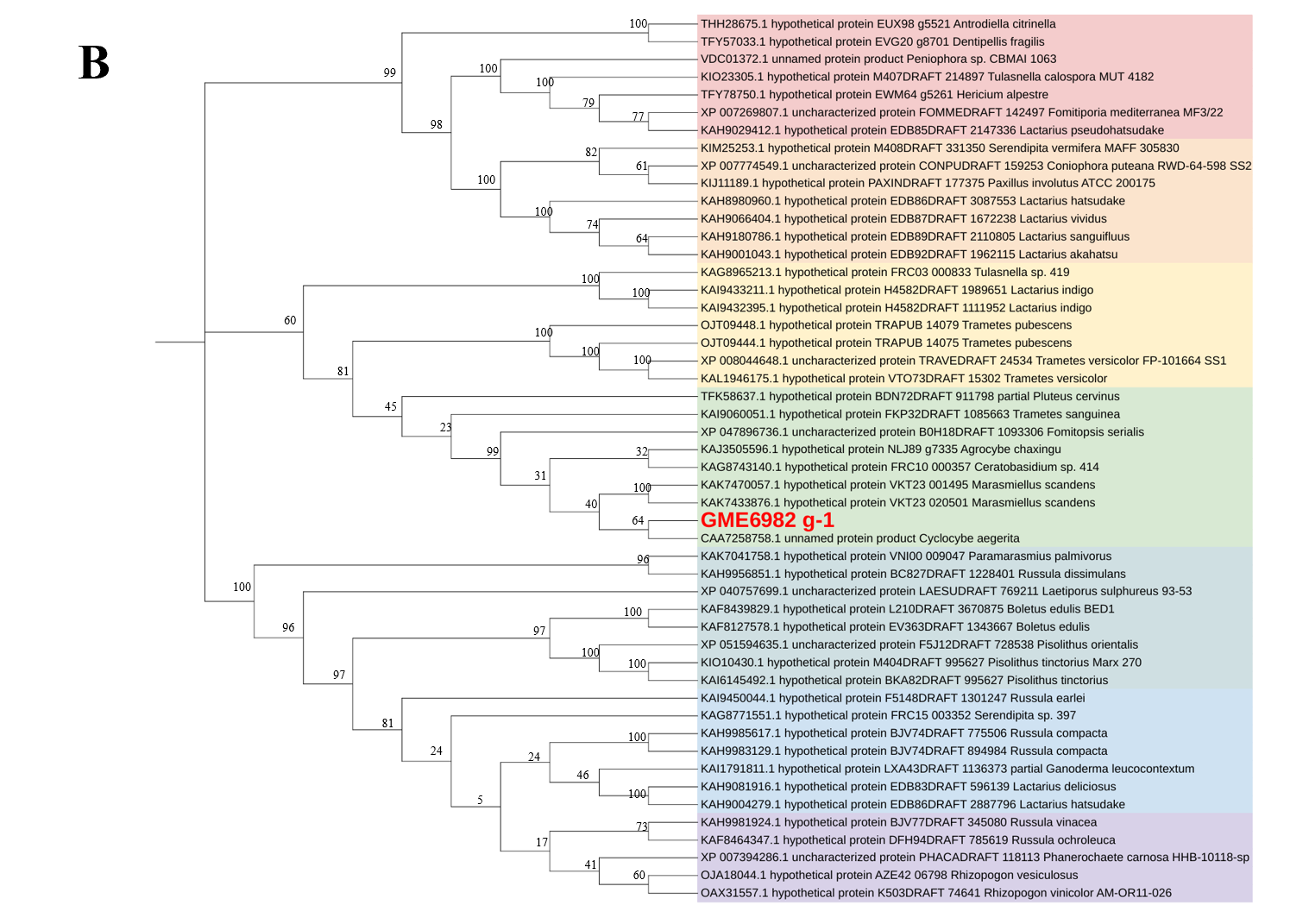
**

## Figure S30 Maximum likelihood tree of PheG-1

a) Maximum likelihood tree of PheG-1 with hydroxyl aldolase using the neighbor-joining method by MEGA 11.0; b) Maximum likelihood tree of the 50 entries with the highest similarity between PheG-1 using the neighbor-joining method by MEGA 11.0.


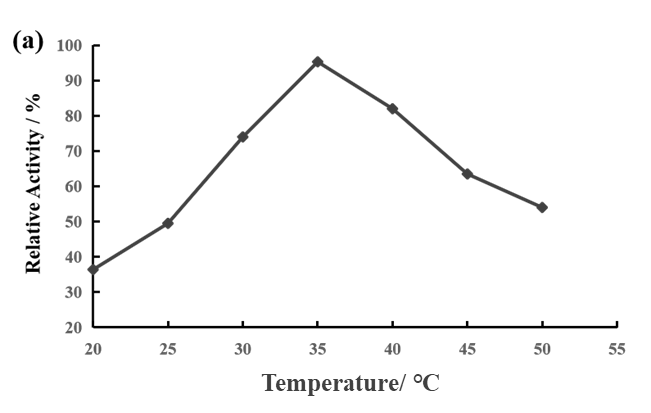

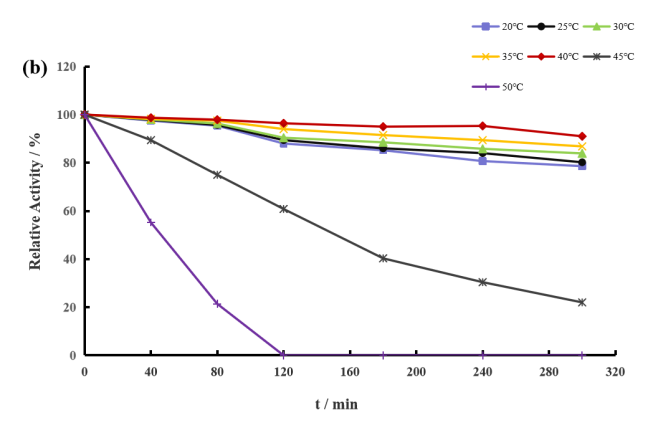


## Figure S31 The optimal temperature and stability of PheG

a) The effect of temperature on PheG activity. The activity was pH 7.5 at 20-50 ℃; b) The effect of temperature on the stability of PheG. The activity was measured under the standard procedure after the enzyme was incubated at 20, 25, 30, 35, 40, 45,50 ℃ in different time intervals and the residual activity was measured according to the standard conditions. Each value represents the average of triplicate experiments.


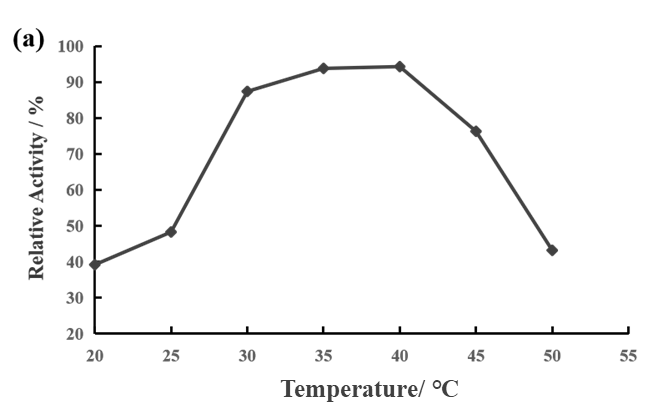
*
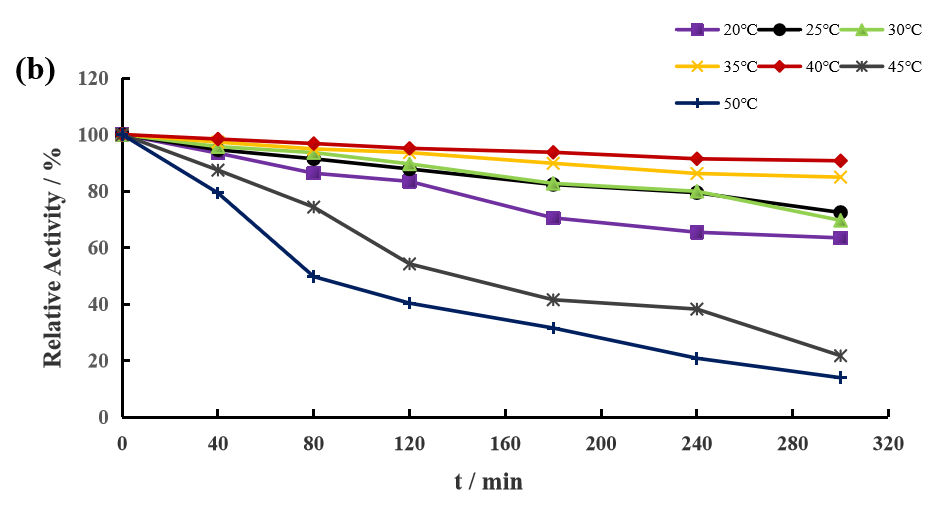
*

## Figure S32 The optimal temperature and stability of PheG-1

a) The effect of temperature on PheG-1 activity. The activity was pH 7.0 at 20-50 ℃; b) The effect of temperature on the stability of PheG-1. The activity was measured under the standard procedure after the enzyme was incubated at 20, 25, 30, 35, 40, 45,50 ℃ in different time intervals and the residual activity was measured according to the standard conditions. Each value represents the average of triplicate experiments.


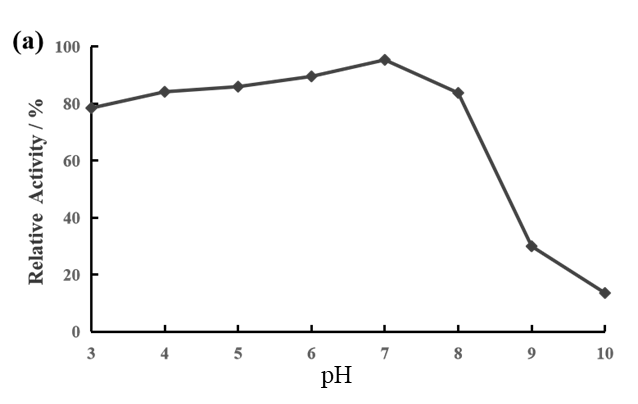


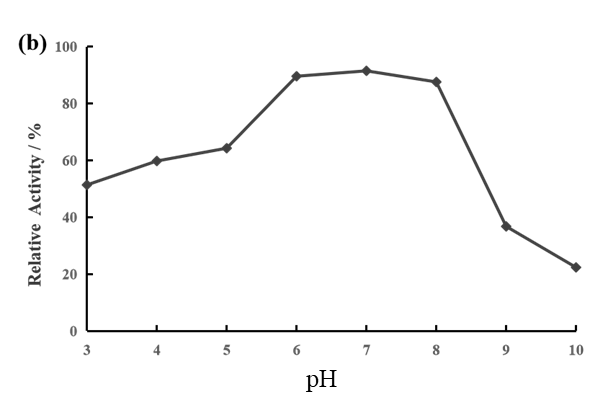


## Figure S33 The optimal pH and stability of PheG

a) The activity was assayed at 35 ℃ in buffers containing a pH range of 3.0-10.0; b) The effect of pH on the stability of PheG. The activity was measured under the standard procedure after the enzyme was in incubated different pH buffers at 35 ℃. Each value represents the average of triplicate experiments. Errors bars represent the standard deviation.


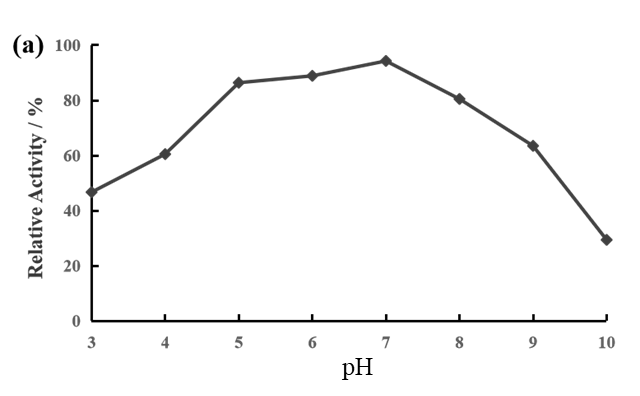


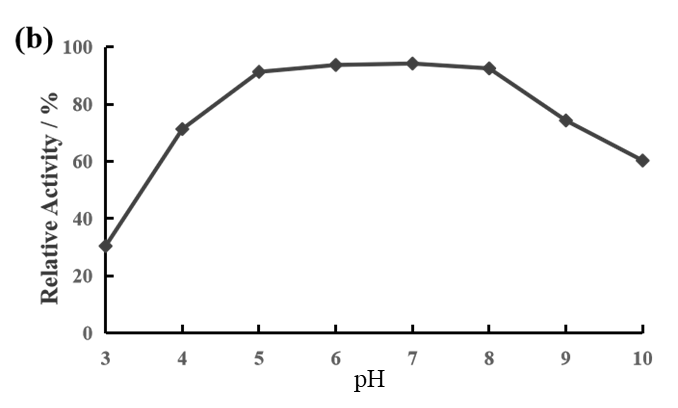


## Figure S34 The optimal pH and stability of PheG-1

a) The activity was assayed at 35 ℃ in buffers containing a pH range of 3.0-10.0; b) The effect of pH on the stability of PheG-1. The activity was measured under the standard procedure after the enzyme was in incubated different PH buffers at 35 ℃. Each value represents the average of triplicate experiments.

**a
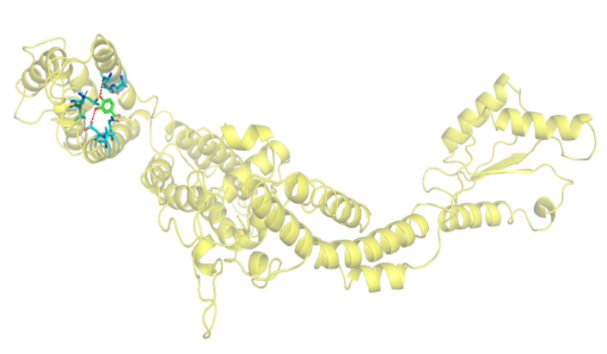
**

**b
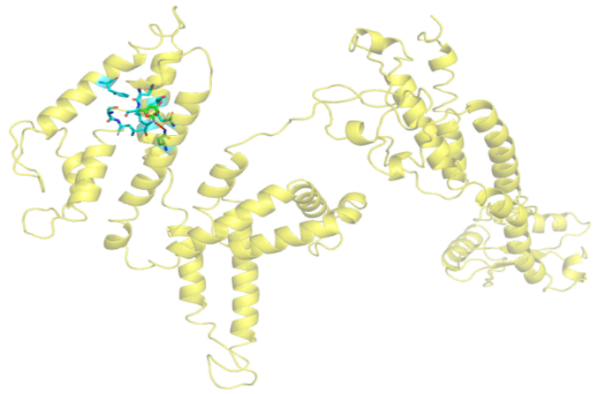
**

## Figure S35 The three-dimensional structure of PheG-1 enzyme protein

The blue-green part represents the hypothetical binding sites of **3** (a) and **2** (b) and PheG-1, respectively


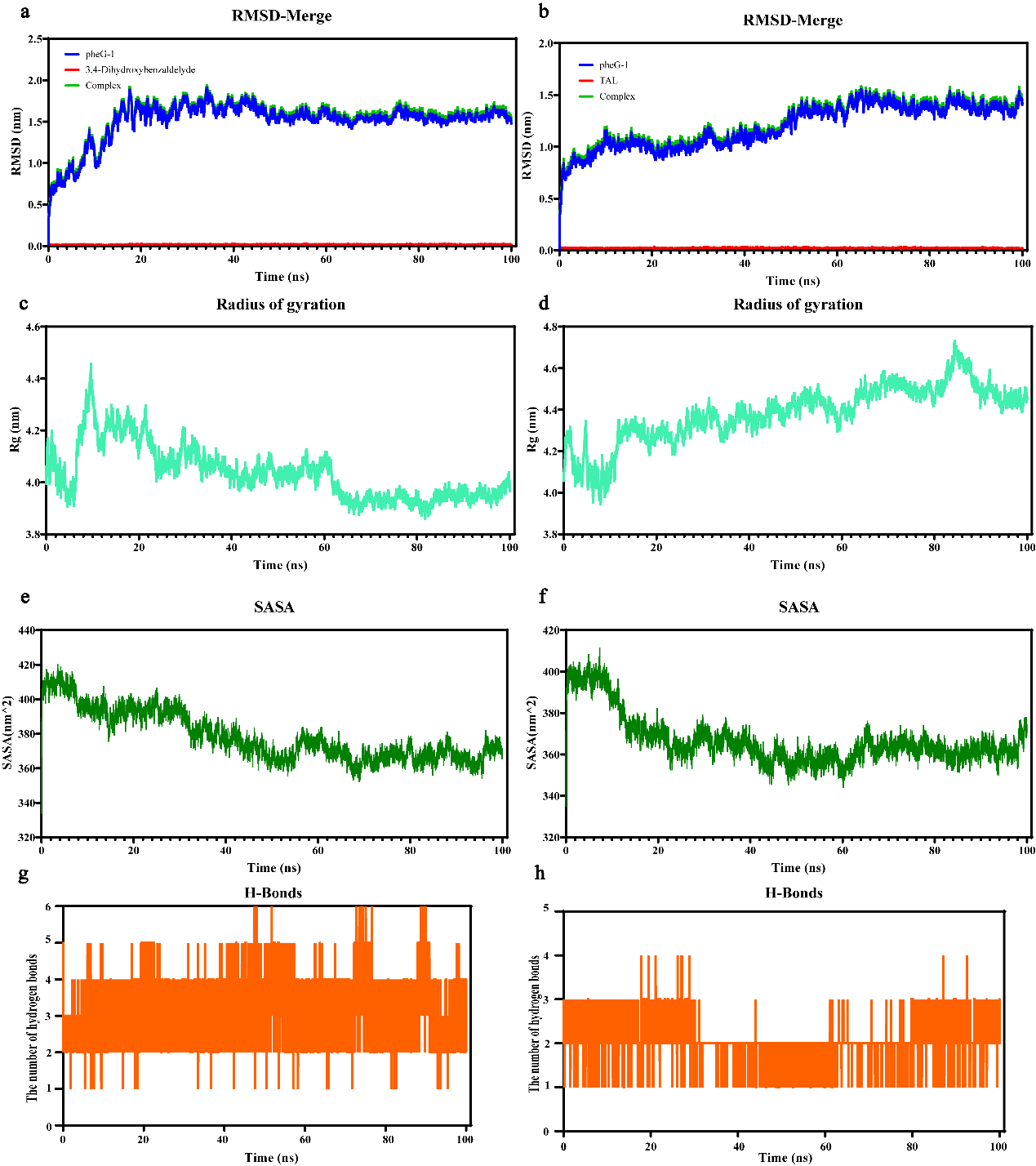


## Figure S36 Analysis of molecular dynamics simulation results

a, b) Root-mean-square deviations (RMSD) curve of PheG-1 (Cyan line), small molecule (Red line), and the PheG-1-3,4-dihydroxybenzaldehyde or PheG-1–**2** complex (Blue line); c, d) Rg curve of the PheG-1–**3** or PheG-1-TAL complex; e, f) Solvent accessible surface area (SASA) variation plot for the PheG-1–**3** or PheG-1–**2** complex; g, h) Variation in the numbers of hydrogen bonds between the ligand and the PheG-1–**3** or PheG-1–**2** complex.

**M 1 2 3**


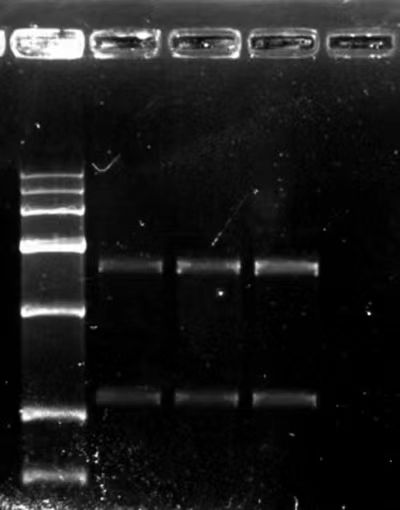


**1000 bp**

**1500 bp**

**3000 bp**

**5000 bp**

**7500 bp**

## Figure S37 The electrophoresis picture of mutant plasmid double digestion (*Nde I-Xba I* restriction sites)

M- Marker; 1 = plasmid after Q386 mutation; 2 = plasmid after D390 mutation; 3 = plasmid after S503 mutation.

**M 1 2 3**


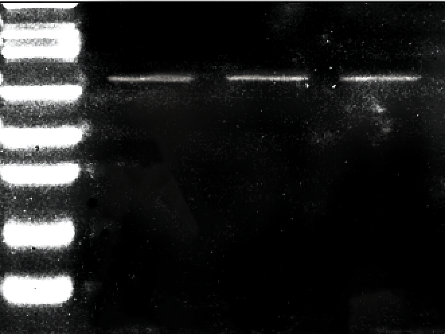


**20 kDa**

**55 kDa**

**35 kDa**

**40 kDa**

**70 kDa**

**100 kDa**

## Figure S38 SDS-PAGE of *His*6-MiCGT purified by affinity chromatography

M = Marker; 1. purified protein expressed by the mutant strain after Q386 mutation; 2. purified protein expressed by the mutant strain after D390 mutation; 3. purified protein expressed by the mutant strain after S503 mutation.

**a**


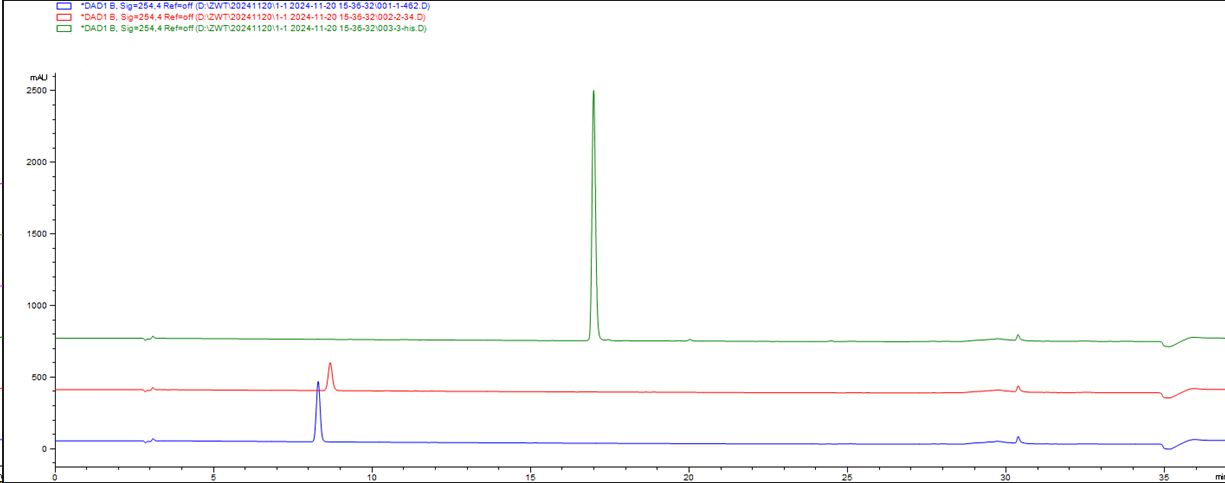


standard of **2**

standard of **3**

standard of **1**

**b**
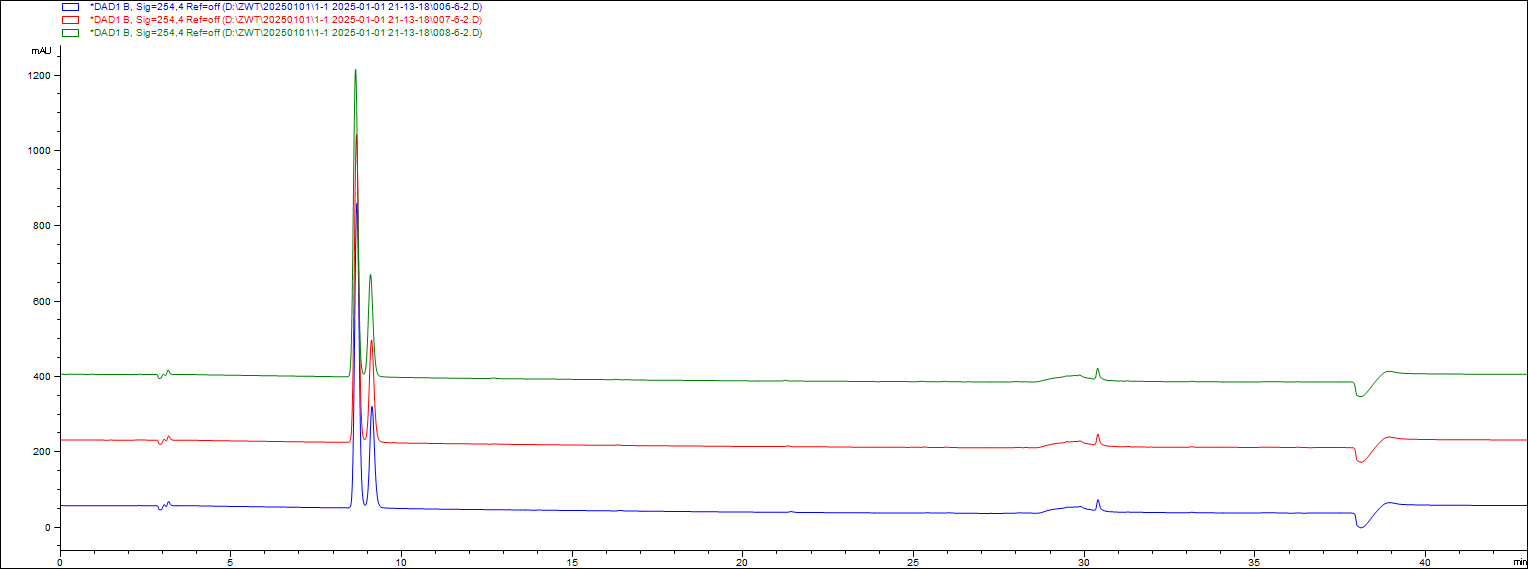


green: 2+3+mutant_S503_

red: 2+3+mutant_D390_

blue: 2+3+mutant_Q386_

## Figure S39 HPLC-DAD of catalytic product by mutants


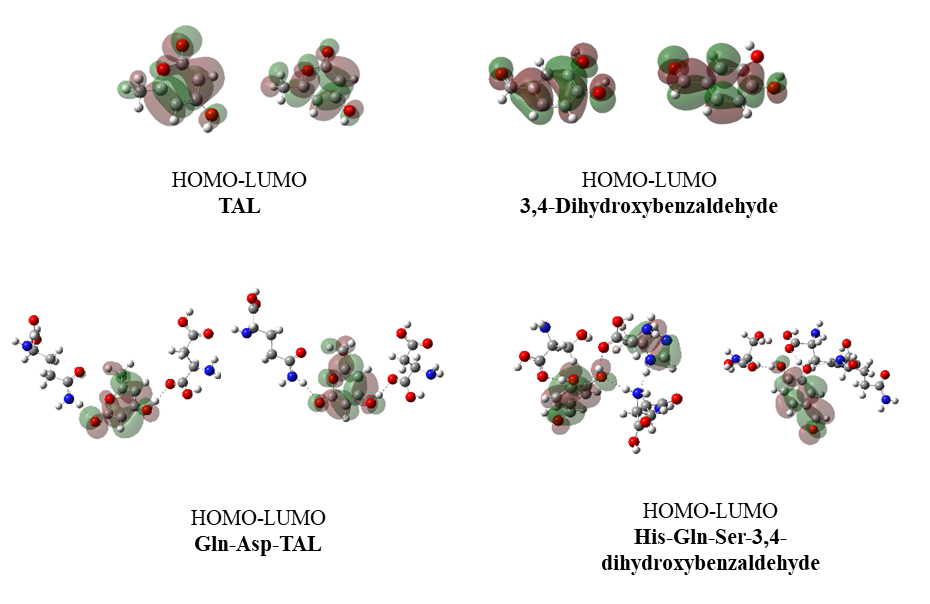


## Figure S40 The orbital distribution of highest occupied molecular orbital (HOMO) and lowest unoccupied molecular orbital (LUMO)

# 3. Supplementary Tables

## Table S1 Bacterial strains and plasmids used and constructed in this study

| Strains/plasmids | Characteristic(s) | source |
| --- | --- | --- |
| pCZN1 | vector | Zoonbio |
| TOP10 | *E. coli* competent cell | Zoonbio |
| *P. igniarius* | *P. igniarius* No 5.95 | CGMCC |
| pCZN1-GME6982 | [recombinant](C:/Users/DELL/Desktop/Application/8.9.9.0/resultui/html/index.html#/javascript:;) [vector](C:/Users/DELL/Desktop/Application/8.9.9.0/resultui/html/index.html#/javascript:;) | This study |
| Arctic-Express^TM^ | Express strain | Zoonbio |

## Table S2 Media used in this study

| Media | Components |
| --- | --- |
| modified Martin medium (MMM) | 5.0 g/L peptone, 1.0 g/L K_2_HPO_3_, 2.0 g/L yeast extract, 20.0 g/L glucose |
| MMM agar | 5.0 g/L peptone, 1.0 g/L K_2_HPO_3_, 2.0 g/L yeast extract, 20.0 g/L glucose, 20.0 g/L agar |
| lysogeny broth (LB) medium | 10.0 g/L tryptone, 10.0 g/L NaCl, 5.0 g/L yeast extract, 1.0 mol/L NaOH |
| LB agar | 10.0 g/L tryptone, 10.0 g/L NaCl, 5.0 g/L yeast extract, 1.0 mol/L NaOH, 15.0 g/L agar |

## Table S3 Primers in PCR experiments used in this study

| Primer name | Primer sequence (3’-5’) |
| --- | --- |
| GME1014_g-F | CCATGGATGTCACAATCAGCGTACAAAGTGC |
| GME1014_g-R | CATATGTTAGTCGGTAACAACGTGCATAGCT |
| GME8208_g-F | CCATGGATGTCTGAGAGCGTTAGTTGGTTTA |
| GME8208_g-R | CATATGCTACCAGGGCAACTGCTCGCCTTTG |
| GME2222_g-F | CCATGGATGGCCATTGAAAGTGTTCTTCAGG |
| GME2222_g-R | CATATGTCACTTATGGGTGTAAGCAGCAGCA |
| GME1353_g-F | CCATGGATGGCCGCCGTCGTCAATCCCTTGC |
| GME1353_g-R | CATATGTTACCATATATCAGTGTTCGACCGA |
| GME5829_g-F | CCATGGATGTCAGCAATCAAGACAGAAACGA |
| GME5829_g-R | CATATGTTACGCATGCCCTCTGACAGGTTTT |
| GME6998_g-F | CATATGATGGCACCCGCTACGTCTCTGGTCA |
| GME6998_g-R | GAATTCTCACCAAGGGAGTCGCAGTGGCGTG |
| GME6982_g-F | CATATGATGTCCGAACCTGAAGTTCCCAAAT |
| GME6982_g-R | TCTAGACTAGTTGCCGCCGTCGTCCCAGTAG |

## Table S4 Gene and translated amino acid sequences used in this study

|  | **Gene sequence** | **Amino acid sequence** |
| --- | --- | --- |
| **GME6982_g** | AGTGAACCGGAAGTGCCGAAAAGCACCACCAGCGAAAGTTTTAATGATCATCCGCTGGAAGAAAATCTGGAAGCCCATGTGCTGAGTAAAGCATTTGATGAAGAATGTGCAGCCTTTGCAAAAACCCATAAACATAGCAGCAGTAGTAGCGATGGTAGCGATAGCGATAGTGATGAAAATAGCCTGTGGAGCAATGGTGACACCAGCAGTAGCGTGAGCAGCGTGGAAGTTAGCCCGCATGATGTTCCGAAACTGGAAGCATATCTGTATTATGCAGGCCTGAGTGGCCCGAGTGGCCGTGGTCCTAAACTGATCTATCGTACCAGCAGCGATAAATTTGTTCCGCCGGATGGCCCGGAAGCCTATCGCCGTCTGATGAAACTGCGCACCGTGCCGGAAAATCATAAACTGGGCGAAGATGGCCTGTGGGATCGTATTCGTGCAGAAGTGGTGAAACTGCTGGATCATACCGGCATTCAGCTGAGCAGTGTGGATCTGGTTCGTTTTACCTGGGTGGAAAAGAATGATGATCAGGAAGATCAGGAAGACCAGGAAGATCAAGAAGATCAGGAGGATCAGGAAGGCCAGGAAGGCCAAGAAGAAGATCAGGAAGATGAAGTTAATGTTAATTACGATGACATCGCCCCGATTAAGCCGGTGGTTGGTGGCACCGTTTATACCACCCCGGTGACCATTTGGGTTGGCGTGATGCCGGATACCACCACCGGTGAACAGGCATATAATAGTAGTCGTGATATTCTGGATCTGCTGCAGCAGTATAATATTACCGATGTTGATGTTGCCTATCGCGAAAGTGAAGTTAAATTTTCAGCAGGTCCGGAACTGTTTGCCCCGGTGAGCGATCTGGATCCGCTGAAAGATGTTATTGATAGCCTGAGTACCCCGCTGAGCCTGCCGATTGCCGGCCTGAAAACCAAAATGCAGGGCACCCTGGGTTTTTATTTTCGCATTGGTGAAGATCTGTATGCAGTTACCGCCCGCCATGTTCTGTTTAAAGATAATGAAGCCAATGTGGAATACAATTATGTGGCCGGTCCGAAAAAAGGCGTTATTGTGATGGGCCCGAATGCATTCACTAATCATCTGGCATTTCTGCAGAGTACCATTGGCACCCTGCTGGATACCGCCGAATATCTGGAAACCCGTGTGACCAGTCTGACCAGCCTGGTTGAAGGCGGTGGTAGCCGTGCCGAACAGAGTGGTCTGGAACTGCCGGAAACCCAGGAACAGCTGACCAAAACCCGTACCAAAATTGATGCCCTGAAAGCACATTTTGTGACCGTGAAAAAGAAATGGAGTAAAGCAAAAGATCGCGTGATTGGTCATGTGGTGTGGGCACCGCCGATTAGCGTGGCAACCCCGCCGCATCAGTATACCCAGGATGTTTGTGTGATTAAGCTGGATAAAGATAAATTCCGCCATTTTCGCCGTAATGTGCTGAGCCTGGGTCCGGAAATTAGTCCGGCCAATTTTAAAAAACTGATGTATGATCGCTTCAACGCCCCGCATGAATTTGTTTATCCGCCGGAAGGTCTGTTTAAACTGCGTGGTATTCTGACCCAGGAAGAAATTCGTACCCCGGATATTAAGGCCCCGCAGCCGCAGGGTGACCCGATTCGTCGCGTTATTAAGCGCGGTTTTACCACCCTGACCACCGTGGGCGGCCTGAGTGGTTTTCTGAGTTATGTTCGTCGCTATTTTGCCACCGGCAATATTGATAGTGTTGAAGCCGCAATTCTGCCGCATAATAATGATAGCGGTCCGTTTAGCCGCGGTGGTGACAGTGGTAGTGTGATTGTGGATGCACTGGGTCGCTTTGTGGCACTGCTGACCGGCGGTACCGGTAAAACCGATAGTAGTGATATTACCTTTGGTACCCCGATGCATTGGCTGTGGCTGCTGATTCTGGCCAAATTTGATGGTGCAAATCTGTATTGGGATGATGGCGGCAATTAATCTAGATAGGTAATCTCTGCTTAAAAGCACAGAATCTAAGATCCCTGCCATTTGGCGGGGATTTTTTTATTTGTTTTCAGGAAATAAATAATCGATCGCGTAATAAAATCTATTATTATTTTTGTGAAGAATAAATTTGGGTG | SEPEVPKSTTSESFNDHPLEENLEAHVLSKAFDEECAAFAKTHKHSSSSSDGSDSDSDENSLWSNGDTSSSVSSVEVSPHDVPKLEAYLYYAGLSGPSGRGPKLIYRTSSDKFVPPDGPEAYRRLMKLRTVPENHKLGEDGLWDRIRAEVVKLLDHTGIQLSSVDLVRFTWVEKNDDQEDQEDQEDQEDQEDQEGQEGQEEDQEDEVNVNYDDIAPIKPVVGGTVYTTPVTIWVGVMPDTTTGEQAYNSSRDILDLLQQYNITDVDVAYRESEVKFSAGPELFAPVSDLDPLKDVIDSLSTPLSLPIAGLKTKMQGTLGFYFRIGEDLYAVTARHVLFKDNEANVEYNYVAGPKKGVIVMGPNAFTNHLAFLQSTIGTLLDTAEYLETRVTSLTSLVEGGGSRAEQSGLELPETQEQLTKTRTKIDALKAHFVTVKKKWSKAKDRVIGHVVWAPPISVATPPHQYTQDVCVIKLDKDKFRHFRRNVLSLGPEISPANFKKLMYDRFNAPHEFVYPPEGLFKLRGILTQEEIRTPDIKAPQPQGDPIRRVIKRGFTTLTTVGGLSGFLSYVRRYFATGNIDSVEAAILPHNNDSGPFSRGGDSGSVIVDALGRFVALLTGGTGKTDSSDITFGTPMHWLWLLILAKFDGANLYWDDGGNSRVISAKHRIDPCHLAGIFLFVFRKIIDRVIKSIIIFVKNKFG |
| **GME6982****_g**-1 | ATGCCTCATGATGTTCCTAAACTGGAAGCATATCTGTATTATGCAGGTCTGTCTGGTCCTTCAGGTCGTGGTCCTAAACTGATTTATCGTACTTCTTCAGATAAATTTGTTCCTCCGGATGGTCCTGAAGCCTATCGCCGTCTGATGAAACTGCGTACCGTTCCGGAAAATCATAAACTGGGTGAAGATGGTCTGTGGGATCGTATTCGCGCCGAAGTTGTGAAACTGCTGGATCATACAGGTATTCAGCTGTCCTCAGTTGATCTGGTTCGTTTTACCTGGGTTGAAAAAAATGATGATCAGGAAGATCAGGAAGATCAGGAAGATCAGGAAGATCAGGAAGATCAGGAAGGTCAGGAAGGTCAGGAAGAAGATCAGGAAGATGAAGTTAATGTTAATTATGATGATATTGCACCGATTAAACCGGTTGTTGGTGGTACCGTTTATACCACCCCGGTTACCATTTGGGTTGGTGTTATGCCGGATACCACCACCGGTGAACAGGCATATAATAGCAGCCGTGATATTCTGGATCTGCTGCAGCAGTATAATATTACCGATGTTGATGTTGCATATCGTGAAAGCGAAGTTAAATTTAGCGCAGGTCCGGAACTGTTTGCACCGGTTAGCGATCTGGATCCGCTGAAAGATGTTATTGATAGCCTGAGCACCCCGCTGAGCCTGCCGATTGCAGGTCTGAAAACCAAAATGCAGGGTACCCTGGGTTTTTATTTTCGTATTGGTGAAGATCTGTATGCAGTTACCGCACGTCATGTTCTGTTTAAAGATAATGAAGCAAATGTTGAATATAATTATGTTGCAGGTCCGAAAAAAGGTGTTATTGTTATGGGTCCGAATGCATTTACCAATCATCTGGCATTTCTGCAGAGCACCATTGGTACCCTGCTGGATACCGCAGAATATCTGGAAACCCGTGTTACCAGCCTGACCAGCCTGGTTGAAGGTGGTGGTAGCCGTGCAGAACAGAGCGGTCTGGAACTGCCGGAAACCCAGGAACAGCTGACCAAAACCCGTACCAAAATTGATGCACTGAAAGCACATTTTGTTACCGTTAAAAAAAAATGGAGCAAAGCAAAAGATCGTGTTATTGGTCATGTTGTTTGGGCACCGCCGATTAGCGTTGCAACCCCGCCGCATCAGTATACCCAGGATGTTTGTGTTATTAAACTGGATAAAGATAAATTTCGTCATTTTCGTCGTAATGTTCTGAGCCTGGGTCCGGAAATTAGCCCGGCAAATTTTAAAAAACTGATGTATGATCGTTTTAATGCACCGCATGAATTTGTTTATCCGCCGGAAGGTCTGTTTAAACTGCGTGGTATTCTGACCCAGGAAGAAATTCGTACCCCGGATATTAAAGCACCGCAGCCGCAGGGTGATCCGATTCGTCGTGTTATTAAACGTGGTTTTACCACCCTGACCACCGTTGGTGGTCTGAGCGGTTTTCTGAGCTATGTTCGTCGTTATTTTGCAACCGGTAATATTGATAGCGTTGAAGCAGCAATTCTGCCGCATAATAATGATAGCGGTCCGTTTAGCCGTGGTGGTGATAGCGGTAGCGTTATTGTTGATGCACTGGGTCGTTTTGTTGCACTGCTGACCGGTGGTACCGGTAAAACCGATAGCAGCGATATTACCTTTGGTACCCCGATGCATTGGCTGTGGCTGCTGATTCTGGCAAAATTTGATGGTGCAAATCTGTATTGGGATGA | PHDVPKLEAYLYYAGLSGPSGRGPKLIYRTSSDKFVPPDGPEAYRRLMKLRTVPENHKLGEDGLWDRIRAEVVKLLDHTGIQLSSVDLVRFTWVEKNDDQEDQEDQEDQEDQEDQEGQEGQEEDQEDEVNVNYDDIAPIKPVVGGTVYTTPVTIWVGVMPDTTTGEQAYNSSRDILDLLQQYNITDVDVAYRESEVKFSAGPELFAPVSDLDPLKDVIDSLSTPLSLPIAGLKTKMQGTLGFYFRIGEDLYAVTARHVLFKDNEANVEYNYVAGPKKGVIVMGPNAFTNHLAFLQSTIGTLLDTAEYLETRVTSLTSLVEGGGSRAEQSGLELPETQEQLTKTRTKIDALKAHFVTVKKKWSKAKDRVIGHVVWAPPISVATPPHQYTQDVCVIKLDKDKFRHFRRNVLSLGPEISPANFKKLMYDRFNAPHEFVYPPEGLFKLRGILTQEEIRTPDIKAPQPQGDPIRRVIKRGFTTLTTVGGLSGFLSYVRRYFATGNIDSVEAAILPHNNDSGPFSRGGDSGSVIVDALGRFVALLTGGTGKTDSSDITFGTPMHWLWLLILAKFDGANLYWDDGGN |
| **GME1014_g** | ATGTCACAATCAGCGTACAAAGTGCCGCGTCAGATCCAACTTACTTCTCGCAAGCCGGACAGACTGCACAGTCAGCTTCAGAATGAGATAAAAGGCGGGAAGCTACTTCCAGCCGTGCCAGCAGACGTCACAAAACCAGACTCCCTTGAACGAGCATTCGAAGGGGCCAACGTGGTCGTTTCGCTTGTCGGACTTATGCACGGAACGCCCCAGATGTTTGAGGAAATCCAACGGAAAGGAGCTGGTAATGTTGCAGCCGCCGCAAAAATACACGGAGCAAAAGTCATACACATCAGTGCCATCGGAGCGGATGAAGCAAGCCAGATTCCGTATGCGCGAACGAAAGCACTCGGTGAAAAGGCTGTGAGGCAAATTTCTCCAGGCGCAACAATCATACGCCCGAGTCTCGTCTTTGGACCAGGCGACGGTTTCTTCGCCGTTAGTACTCGGCCAGCTATGCACGTTGTTACCGACTAA | MSQSAYKVPRQIQLTSRKPDRLHSQLQNEIKGGKLLPAVPADVTKPDSLERAFEGANVVVSLVGLMHGTPQMFEEIQRKGAGNVAAAAKIHGAKVIHISAIGADEASQIPYARTKALGEKAVRQISPGATIIRPSLVFGPGDGFFAVSTRPAMHVVTD |
| **GME1353_g** | TTGACATCGATGAAGCAGACAGTACTTTTAGACTTGCTCACTGGACCGTGAGTGACGGTCCGGCGGGCCGAACGTCAGGAAATAGGGACCCATGCATTAATAAATATGGCCGCCGTCGTCAATCCCTTGCACCCCGACATAATCCCAAGGCTTGATCCAGAGTTCGCTGCATACTATAACGCTCATCAAGCAAACAAGCTTGGGCCTCATCAGATTCCTTGGGTACCAGCTATTCGTAAGAAGCCCCCAGTCGACGGGGCATCTCAGCCATTGAAGGTCGGTCTTACGAAGGATATACGCTTGTCGAAATTTTCAGTACGAGTATTCTGGCCGGAGGATCCCTCCAAGTCTCCAGAAGCGGATGGCCGGTCTTCCTGTTCTTCCATGGCGGCGGATGGACACTTGGAAATATTGATACTGAGAACCACTTCTCCAGCAACATGTGTCTGAAAGCGAAATGCATAGTTGTTTCGTGGATTACCGCCTTGGACCGGAAGAGCAGTATCCTGCTGCTGTTGAAGATGCCGAGGAGCGTTTCGATGGATTGTCGAGCACGGCAAATCAGAGCTCAAAGCAGATCTGTCCCGGTTCGCGGTTGGTGGCTCTTCAAGTGGTGGTAACCTTGCTGCTATCATCTCTCATAAGGCAGCCCTTTGCGAACCGCCCGTCCCGTTGATATTTCAAGTGTTAGTGGTCCCAGTCACGGACAACACCGCTTCCGTATCTGGCCCCTATGCTTCATGGCAGGAGAACCGTAACACGCCAGCCCTGACGCCAGAGAAAATGCTTTGGTTCAAGAATAATTATTCACCAAATCCAGAGGACTGGAAGAAGTGGGATAGCTCCCCAATATTTGCACCGGAAGAATCCTTTAAAAAGGTTCCTGATGCATGGGTTGGAGTGGCCGAACTCGATATCCTGAGAGACGAGGGGTTAGCTTATGCCGAGAAGATCAGGAAGGCTGGCCATAATGTAGAAGTCAAGATTTACAAAGGCTCGCCGCATCCGATCATGGCCATGGATGGGTGAGGGTCATTATCTAGCTTGTCGTTCTTGAAGAGTCTAACGCGGAGTGCATAGTGTGCTACAATCTGGAAGAGACTTCATTGCAGACGCGGCGGTTGCTCTCAGGCGAGCATTTTACGGCGATTCGGTCGAACACTGATATATGGTAAGAGTAGTTAGATGGTGCATATAGGAGAGTTCAAGATGGTAGTAACTC | LTSMKQTVLLDLLTGPVTVRRAERQEIGTHALINMAAVVNPLHPDIIPRLDPEFAAYYNAHQANKLGPHQIPWVPAIRKKPPVDGASQPLKVGLTKDIRLSKFSVRVFWPEDPSKSPEADGRSSCSSMAADGHLEILILRTTSPATCVKRNALFRGLPPWTGRAVSCCC*RCRGAFRWIVEHGKSELKADLSRFAVGGSSSGGNLAAIISHKAALCEPPVPLIFQVLVVPVTDNTASVSGPYASWQENRNTPALTPEKMLWFKNNYSPNPEDWKKWDSSPIFAPEESFKKVPDAWVGVAELDILRDEGLAYAEKIRKAGHNVEVKIYKGSPHPIMAMDGGSLSSLSFLKSLTRSACATIWKRLHCRRGGCSQASILRRFGRTLIYGKSSMVHIGEFKMVVT |
| **GME2222_g** | ATGGCCATTGAAAGTGTTCTTCAGGCTGCAAGTCGAGCTGACTCTGGATTCAAGAACATTCCTATCATTGATATGAAGGACGTGGATAATCCCGACCCTGTTGCGCGACAGCAGCTCGCGAAAGACATTCGGGATGCTTGTATCAACGTCGGTTTCTTCTACGTCAAGAACCACGGAATATCCGAGAAGAGTATTTCTGGAGCCGTCGAGGCAGGAAAGAAGTTTTTTAATCTTGATGAGGAGACAAAGCTCAAATATGACGCTACGAAATCTGCGAATTTCATGGGCTATACAGCCCTCTTGAGTGAGAATACCGATCCGGAGAACAGAGGTGACCTTCATGAGGCGTTCGACACCGGCCCAGAAGAGACGGAGGTCACGAAGAACTCCAGCGTAATGTCTGGTGCTAATCCTTGGCCTGAAAACGAAGTCCCGGGATTCAGAGAAGGCTATCTGAATTATTACAACGAGGCGATAGCATTTGGAAAGAAGCTCTTTCCATTGTTCGCGATTGCATTGGGCCTTCCGGAGACATTCTTTGATGACAAGACAAAAGAAGATGCTGCAGTTATGCGTGTCATTCGGTACCCACCACAGGCGGGGCCTCATGACGACAGAGTCATCGGGATAGGCGCACATACTGACTTCCAGTGCTTCACAATACTCTGGCAGGAGCCTGGTGTCCAAGCACTACAAGTGTTGAATGCCAATAAAGAGTGGGTGAACGCGACACCTATACCTGGAACGCTCGTCATCAACATTGCGGATCAGCTTTCAAGATGGACAAATGATGTCTTCAAGTCAACCGTCCATCGCGCAATCAACCGTTCAGGAGTAGAGCGCCATTCAATGCCGCTTTTCTTCGGTGTCGACTACAACGTCGAGCTTGAGCCGATACCGAGTTGCGTTTCTTCTGAATGTCCGTCGAAGTACGAAGTCGTGACAGCCGGCGAATACGTCAAAGCTCGACTTGCTGCTGCTTACACCCATAAGTGA | MAIESVLQAASRADSGFKNIPIIDMKDVDNPDPVARQQLAKDIRDACINVGFFYVKNHGISEKSISGAVEAGKKFFNLDEETKLKYDATKSANFMGYTALLSENTDPENRGDLHEAFDTGPEETEVTKNSSVMSGANPWPENEVPGFREGYLNYYNEAIAFGKKLFPLFAIALGLPETFFDDKTKEDAAVMRVIRYPPQAGPHDDRVIGIGAHTDFQCFTILWQEPGVQALQVLNANKEWVNATPIPGTLVINIADQLSRWTNDVFKSTVHRAINRSGVERHSMPLFFGVDYNVELEPIPSCVSSECPSKYEVVTAGEYVKARLAAAYTHK |
| **GME5829****_g** | ATGTCAGCAATCAAGACAGAAACGACCCAACCACCCGTCGAATATCGTCGACTGGGTTCCTCCGGTCTGCGCGTATCAGTCCCCATCCTCGGCGCCATGTCCTTCGGCTCCTCCGCCTGGGCCCCATGGGTCGTCAACGAGGACGCCGCACTCCCGCTCCTCAAAGCCGCCTGGGAGCGGGGCGTCACCACCATTGACACCGCCAACGTCTACTCCAACGGCGAGTCCGAACGCATCATCGGCAAGTTCCTCCGTGACGAGAAGATCCCGCGCGAGAAGGTCGTCATCGCCACCAAGTGTAGCGGGCTCGTCGCGGACGATGTGGGTATCAGAACGTATCTGAACCCTGAGATGAGGGATATGAGGGATTATGTGAATCAGAGTGGGCTGAGTCGGGCGGCGATTTTTAACGCTGTTGAGAAATCTTTGGAGAGGCTGGGTATCGCGTACATCCATCGATACGACCCCAACACTCCACCAGCAGAGACGATGCGCGCACTGCACGACCTCGTCCAATCCGGCAAAGTGCGCTACATCGGCGCAAGCTCGATGCGCACCTGGCAGTTTGCCGAGCTCAACCACATCGCGGAGAAGAACGGCTGGACACAATTCATCAGCATGCAGAACGAATATTCACTGCTCTATCGAGAGGAGGAACGCGAAATGATCCCATACTGCAACGCACACGGCATCGGTCTCATCCCCTGGGGTCCCCTCTCCGCAGGCGACCTCGCCTACCCACTCGGCACATCCACAACCCGCCGCGACGCCGCTTCGCGCGCCTATTCCTCCTCCGACAACGAAATCATCAACCGCGTCGACGAGCTCGCCAAAAAGCGCGGGTGGACCATGAGCCAGGTCGCCCTGGCGTGGATCGGCGCAAAAGTGTCCAGCCCTATCGTTGGCGTTAATTCGCCGGAACGCCTTGAACAGGCCATCATATCTGGGAAAGAGCTCACCGAGGACGAAATCAAGTACCTCGAAGAACCATACGTTCCAAAACCTGTCAGAGGGCATGCGTAA | MSAIKTETTQPPVEYRRLGSSGLRVSVPILGAMSFGSSAWAPWVVNEDAALPLLKAAWERGVTTIDTANVYSNGESERIIGKFLRDEKIPREKVVIATKCSGLVADDVGIRTYLNPEMRDMRDYVNQSGLSRAAIFNAVEKSLERLGIAYIHRYDPNTPPAETMRALHDLVQSGKVRYIGASSMRTWQFAELNHIAEKNGWTQFISMQNEYSLLYREEEREMIPYCNAHGIGLIPWGPLSAGDLAYPLGTSTTRRDAASRAYSSSDNEIINRVDELAKKRGWTMSQVALAWIGAKVSSPIVGVNSPERLEQAIISGKELTEDEIKYLEEPYVPKPVRGHA |
| **GME6998_g** | ATGGCACCCGCTACGTgCTCTGGTCATAGGTGCATCTCGCGGGCTGGGCCTCGAGTTGGTACGCAACTTGCACGCTCATGGCTCTCAAGTCTTCGCAACGGTGCGCTCGTCACCTCAATCGCCCGACCACTTCCCGAGTGGTGTTACAGTTATCGACGGAGTCGACCTTGGAGACGAGAATGCAGGAGATGTGATTGTGAAAGGGCTCAATGGCAAGAAGGTCGACCTCACGATTATCAATGCTGGCGTGTTCAGGACTGAGACGCTTGAGAAGCCAAATTATGCGGCTGAACTTGAAATGTACAAAACAGTTGCTATTGCACCAGTTTTCCTCACCTATCATCTACGAAAGTCCAATTGCTTTGCTCCAAATGGGAAGCTTGTGCTCATAACGACAGAGGGAGGCTCCATCTCGCTTCGTACAAAGGAAGAAGGCGGTGGAAACTATGGACACCATGCTAGCAAAGCTGCCGTGAACATGGTCGGCAGGCTATTGTCGCATGACCTTCTCGATGACGGTGTTGCAGTCGTTATGATACATCCTGGGTTCATGCGGACGGACATGACGAAAGGAGTAGGGTTCGATAAGTTCTATGATTCTGGTGGTGCTGTGTTGCCTTCGGAGGCGGCCGCCTCGACCCTCGACTTTGTTGAGAGTTTTTCCATCAAAGATACGGGCTCGTTTTGGGCACCGCGCGGTCCAAGAGACATTGGTGAGGCGGGACGTGTTCTCGGGAAGGACCTCCCCACGCCACTGCGACTCCCTTGGTGA | MAPATSLVIGASRGLGLELVRNLHAHGSQVFATVRSSPQSPDHFPSGVTVIDGVDLGDENAGDVIVKGLNGKKVDLTIINAGVFRTETLEKPNYAAELEMYKTVAIAPVFLTYHLRKSNCFAPNGKLVLITTEGGSISLRTKEEGGGNYGHHASKAAVNMVGRLLSHDLLDDGVAVVMIHPGFMRTDMTKGVGFDKFYDSGGAVLPSEAAASTLDFVESFSIKDTGSFWAPRGPRDIGEAGRVLGKDLPTPLRLPW |
| **GME8208_g** | ATGTCTGAGAGCGTTAGTTGGTTTATTACCGGGGCCTCCCGAGGAATAGGCCTTGAAATAACGAAGCAATTGATTTCGAATCCCAGCAATATTGTGATAGCGTCCTGTCGGAATCCCGAGACGGCCAAGGACTTGTCTGCCCTGAAAACAAAGAAGCCGGGGGGACAGCTGTACATTATTAAGTTAGATGTGACAGTCGAAGAAACAATCAAAGACGCAGCGGCGGGAGCGAGTTCGATACTGGGAGACAAAGGGTTGGACTACCTCCTCAACAACGCAGGCGTCGTCGAAGATTTACTTCAGAATCCCGTTGATGACGGAGCGTTCGATTTTGATATCGCAACCCTCTCATTTGTCATGCAGAGCAATGTGGCTGGGACCGCCCTGGTCACGAGGAGTTTCCTACCCCTCCTCAGGAAAGGTAATAGGAGAGTTGTGATGAATATGTCTAGTGGACTCGGTGGTATCGGGCTGGACTGTGGAAGCAAATGCTGCACATATTCGATTTCAAAAGCAGCTGTGAATATGTTGACGTACAAACAAGCAAAGATCGAGCCGGAAATCAGTGCAATTGCTGTCGACCCAGGCTGGGTAAAGACAAGGTTGGGTGGAGAGGGTGCCGTTCTCGAACCAGCTGAATGCGTCACACCGCTCATCAGCTTTTTGAAAGAGGTGAAGAAGGAACATTCGGGTAAGTTTTTCAATCGCAAAGGCGAGCAGTTGCCCTGGTAG | MSESVSWFITGASRGIGLEITKQLISNPSNIVIASCRNPETAKDLSALKTKKPGGQLYIIKLDVTVEETIKDAAAGASSILGDKGLDYLLNNAGVVEDLLQNPVDDGAFDFDIATLSFVMQSNVAGTALVTRSFLPLLRKGNRRVVMNMSSGLGGIGLDCGSKCCTYSISKAAVNMLTYKQAKIEPEISAIAVDPGWVKTRLGGEGAVLEPAECVTPLISFLKEVKKEHSGKFFNRKGEQLPW |

## Table S5 Effects of various metal ions and inhibitory additives on PheG

| Metal ions | Concentration (mM) | Relative activity (%) |
| --- | --- | --- |
| Control | - | 100.0 |
| K^+^ | 5 | 89.4 |
| Na^+^ | 5 | 102.3 |
| Cu^2+^ | 5 | 103.6 |
| Zn^2+^ | 5 | 94.5 |
| Mg^2+^ | 5 | 96.8 |
| Ca^2+^ | 5 | 104.7 |
| EDTA | 5 | 87.8 |

Data was shown as mean values. Various metal ions and other chemical compounds were added into the reaction mixture to a final concentration of 5 mM. The activity was determined at pH 7.0 and 35 ℃. The enzyme activity without any additives was defined as 100%. All determinations were performed in triplicate.

## Table S6 Effects of various metal ions and inhibitory additives on PheG-1

| Metal ions | Concentration (mM) | Relative activity (%) |
| --- | --- | --- |
| Control | - | 100.0 |
| K^+^ | 5 | 90.5 |
| Na^+^ | 5 | 99.7 |
| Cu^2+^ | 5 | 101.4 |
| Zn^2+^ | 5 | 96.2 |
| Mg^2+^ | 5 | 93.9 |
| Ca^2+^ | 5 | 103.4 |
| EDTA | 5 | 90.3 |

Data was shown as mean values. Various metal ions and other chemical compounds were added into the reaction mixture to a final concentration of 5 mM. The activity was determined at pH 7.0 and 40 ℃. The enzyme activity without any additives was defined as 100%. All determinations were performed in triplicate.

## Table S7 Primer sequence used for site-directed mutations in this study

| Primer name | Primer sequences 5'-3' |
| --- | --- |
| D390F | TATACCCAGGCTGTTTGTGTTATTAAACTGGATAAAGATAAATTTCG |
| D390R | CTGATGCGGCGGGGTTGC |
| S503F | TAATATTGATGCCGTTGAAGCAGCAATTC |
| S503R | CCGGTTGCAAAATAACGAC |
| Q386F | CCCGCCGCATGCGTATACCCAG |
| Q386R | GTTGCAACGCTAATCGGC |

Red nucleotides are the positions chosen for the mutations.

## Table S8 Gbiss free energy and front-line orbital energy before and after pheG-1 participates in the catalytic reaction

|  | Gibbs/_Hartree_ | HOMO_(Hartree/mol)_ | LUMO_（Hartree/mol）_ |
| --- | --- | --- | --- |
| **TAL** | 0.079656 | -0.22549 | -0.04279 |
| **3,4-dihydroxybenzaldehyde** | 0.0852 | -0.22678 | -0.05348 |
| **Hispidin** | 0.162917 | ———— | ———— |
| **Gln-Asp-TAL** | 0.327415 | -0.20373 | -0.01834 |
| **His-Gln-Ser-3,4-Dihydroxybenzaldehyde** | 0.476899 | -0.21806 | -0.04895 |
| **H_2_O** | 0.00378 | ———— | ———— |
| **△**G1 = 0.05 ev | | **△**G2 = -1.99 ev | |
| HOMO-LUMO Gap1 = 4.68ev | | HOMO-LUMO Gap2 = 4.21ev | |

△G1 represents the Gibb free energy change without pheG-1 participating in the catalytic reaction; △G2 represents the Gibbs free energy change in the catalytic reaction; Gap1 represents the front orbital energy gap without pheG-1 participating in the catalytic reaction; Gap2 represents the front orbital energy gap in the catalytic reaction.

1 Hartree = 27.21ev = 627.5Kcal/mol

## Table S9 Absolute values and cartesian coordinates (Å) for optimized structures

**TAL**

| Zero-point correction= | 0.112188 (Hartree/Particle) |
| --- | --- |
| Thermal correction to Energy= | 0.120220 |
| Thermal correction to Enthalpy= | 0.121165 |
| Thermal correction to Gibbs Free Energy= | 0.079656 |
| Sum of electronic and zero-point Energies= | -457.796005 |
| Sum of electronic and thermal Energies= | -457.787972 |
| Sum of electronic and thermal Enthalpies= | -457.787028 |
| Sum of electronic and thermal Free Energies= | -457.828536 |

| C | -1.10166266 | -0.67491136 | -0.00012743 |
| --- | --- | --- | --- |
| C | 0.08356455 | -1.34057663 | -0.00009328 |
| C | 1.29776276 | -0.57555851 | -0.00002116 |
| C | 1.25905649 | 0.79162765 | -0.00004591 |
| C | 0.01040102 | 1.50154456 | -0.00006415 |
| H | 0.09910038 | -2.42697540 | 0.00000649 |
| H | 2.16912345 | 1.38024359 | -0.00003053 |
| O | -0.18636657 | 2.68784238 | 0.00005896 |
| O | -1.16213698 | 0.66529681 | -0.00001533 |
| C | -2.45541542 | -1.31340864 | 0.00009248 |
| H | -3.02444351 | -0.99527427 | 0.88297824 |
| H | -3.02457150 | -0.99549060 | -0.88278437 |
| H | -2.37773955 | -2.40489969 | 0.00022611 |
| O | 2.50093929 | -1.19503560 | 0.00017609 |
| H | 2.37680440 | -2.15473465 | -0.00059691 |

**3,4-dihydroxybenzaldehyde**

| Zero-point correction= | 0.118445 (Hartree/Particle) |
| --- | --- |
| Thermal correction to Energy= | 0.126986 |
| Thermal correction to Enthalpy= | 0.127930 |
| Thermal correction to Gibbs Free Energy= | 0.085200 |
| Sum of electronic and zero-point Energies= | -495.887495 |
| Sum of electronic and thermal Energies= | -495.878953 |
| Sum of electronic and thermal Enthalpies= | -495.878009 |
| Sum of electronic and thermal Free Energies= | -495.920740 |

| C | -1.24434852 | -0.32004103 | -0.00018065 |
| --- | --- | --- | --- |
| C | -0.47913730 | 0.86199809 | -0.00020235 |
| C | 0.90068045 | 0.78481420 | -0.00019871 |
| C | 1.54829275 | -0.47248333 | 0.00014713 |
| C | 0.78992748 | -1.64349368 | -0.00003308 |
| C | -0.60224147 | -1.56445933 | -0.00021325 |
| H | -0.99900668 | 1.81904816 | -0.00023621 |
| H | 1.30994223 | -2.59744791 | -0.00006047 |
| H | -1.19760657 | -2.47589586 | -0.00031656 |
| C | -2.71509578 | -0.23646805 | -0.00008609 |
| H | -3.23263157 | -1.22440909 | -0.00073755 |
| O | -3.35304695 | 0.79814249 | 0.00053748 |
| O | 1.75696247 | 1.86081179 | -0.00027008 |
| H | 1.25348893 | 2.68582084 | 0.00011182 |
| O | 2.90036424 | -0.53851992 | 0.00039806 |
| H | 3.24310987 | 0.37020776 | 0.00051726 |

**Hispidin**

| Zero-point correction= | 0.238774 (Hartree/Particle) |
| --- | --- |
| Thermal correction to Energy= | 0.257307 |
| Thermal correction to Enthalpy= | 0.258251 |
| Thermal correction to Gibbs Free Energy= | 0.186556 |
| Sum of electronic and zero-point Energies= | -969.991836 |
| Sum of electronic and thermal Energies= | -969.973302 |
| Sum of electronic and thermal Enthalpies= | -969.972358 |
| Sum of electronic and thermal Free Energies= | -970.044053 |

| C | -4.73718009 | 0.52517957 | 0.00001705 |
| --- | --- | --- | --- |
| C | -4.47668548 | -0.82076166 | -0.00020590 |
| C | -3.13283349 | -1.30283670 | -0.00024273 |
| C | -2.10599249 | -0.39447647 | 0.00002818 |
| C | -3.67187111 | 1.48711293 | 0.00020058 |
| H | -5.75342799 | 0.90142831 | 0.00001574 |
| H | -2.91197232 | -2.36684376 | -0.00037944 |
| O | -2.35013611 | 0.92999645 | 0.00025796 |
| O | -5.52086174 | -1.68378130 | -0.00040870 |
| H | -5.19275977 | -2.59414800 | -0.00064784 |
| O | -3.73835982 | 2.68924779 | 0.00035034 |
| C | -0.70641503 | -0.76582134 | -0.00004319 |
| H | -0.51388488 | -1.83669467 | -0.00031830 |
| C | 0.30558714 | 0.13447141 | 0.00016097 |
| H | 0.02531908 | 1.18704436 | 0.00035464 |
| C | 1.73668080 | -0.13765525 | 0.00015992 |
| C | 2.28826674 | -1.43367170 | 0.00043870 |
| C | 2.62619835 | 0.95948501 | -0.00013250 |
| C | 3.66551997 | -1.63019241 | 0.00038365 |
| H | 1.63547926 | -2.30239479 | 0.00077834 |
| C | 3.99900086 | 0.76495978 | -0.00022514 |
| H | 2.22518570 | 1.97324927 | -0.00032593 |
| C | 4.53421718 | -0.53682686 | 0.00004489 |
| H | 4.09369761 | -2.62901919 | 0.00064808 |
| O | 5.87671433 | -0.73123992 | 0.00000294 |
| H | 6.30275234 | 0.14115356 | -0.00020370 |
| O | 4.93765598 | 1.77064037 | -0.00050751 |
| H | 4.50254988 | 2.63351992 | -0.00098872 |

**Gln-Asp-TAL**

| Zero-point correction= | 0.404305 (Hartree/Particle) |
| --- | --- |
| Thermal correction to Energy= | 0.436325 |
| Thermal correction to Enthalpy= | 0.437269 |
| Thermal correction to Gibbs Free Energy= | 0.327415 |
| Sum of electronic and zero-point Energies= | -1501.592466 |
| Sum of electronic and thermal Energies= | -1501.560446 |
| Sum of electronic and thermal Enthalpies= | -1501.559502 |
| Sum of electronic and thermal Free Energies= | -1501.669356 |

| C | 0.14414737 | -1.22640112 | -0.90557264 |
| --- | --- | --- | --- |
| C | 1.44796597 | -1.40592567 | -0.56892466 |
| C | 1.82590730 | -2.61932517 | 0.10406632 |
| C | 0.87006766 | -3.56114446 | 0.41450614 |
| C | -0.50165010 | -3.34593662 | 0.09121189 |
| H | 2.18799598 | -0.64935333 | -0.80485306 |
| H | 1.13252696 | -4.47144863 | 0.94076402 |
| O | -1.47063809 | -4.03892434 | 0.34459946 |
| O | -0.78731773 | -2.15531764 | -0.61799293 |
| C | -0.44296058 | -0.04621247 | -1.61069669 |
| H | -0.73954386 | -0.32530374 | -2.63157286 |
| H | -1.35337995 | 0.26966521 | -1.08685717 |
| H | 0.27792109 | 0.77556991 | -1.66797000 |
| O | 3.09936132 | -2.84960711 | 0.44406517 |
| H | 3.67732596 | -2.09840578 | 0.18753012 |
| H | -8.44628713 | 3.01899519 | -2.03513910 |
| C | -7.82747646 | 2.49514712 | -0.33845503 |
| C | -7.42253638 | 1.25900036 | 0.46867496 |
| H | -8.21075081 | 0.50658747 | 0.32400783 |
| C | -6.09750232 | 0.69417153 | -0.08983264 |
| H | -5.29041700 | 1.41986739 | 0.07304164 |
| H | -6.19006876 | 0.56784731 | -1.17534517 |
| C | -5.69782284 | -0.62833747 | 0.56778459 |
| H | -6.39924268 | -1.42814982 | 0.28904440 |
| H | -5.76810880 | -0.51546770 | 1.65829287 |
| C | -4.25921872 | -1.03101806 | 0.22202873 |
| N | -4.02296840 | -2.36834033 | 0.24205792 |
| H | -4.74681018 | -3.00837671 | 0.52881942 |
| H | -3.08456069 | -2.75108360 | 0.14151171 |
| O | -3.40366791 | -0.18913061 | -0.03399673 |
| N | -7.30621322 | 1.53342317 | 1.89703348 |
| H | -6.69582801 | 2.34015673 | 2.02192914 |
| H | -8.21121062 | 1.81918341 | 2.26519884 |
| O | -7.78536242 | 3.63549257 | 0.06128714 |
| O | -8.24008898 | 2.17814555 | -1.59035446 |
| H | 6.25343249 | 5.10538945 | 0.52522796 |
| C | 6.66730429 | 3.28003272 | 0.35513239 |
| C | 6.08141284 | 1.90254224 | 0.59902656 |
| H | 6.39706152 | 1.59194998 | 1.60646879 |
| H | 4.98959250 | 1.95507038 | 0.60422690 |
| C | 6.54423524 | 0.85015061 | -0.42964879 |
| H | 6.19421317 | 1.15491189 | -1.42612129 |
| C | 5.83028638 | -0.47276093 | -0.08477190 |
| O | 6.61528945 | -1.38064236 | 0.46943798 |
| H | 7.51743273 | -0.95646249 | 0.48661910 |
| O | 4.64116877 | -0.64081454 | -0.27820204 |
| N | 7.99500541 | 0.61680416 | -0.36079829 |
| H | 8.34466213 | 0.30419182 | -1.26410349 |
| H | 8.47209638 | 1.49082221 | -0.14589847 |
| O | 7.79164016 | 3.50314889 | -0.04148931 |
| O | 5.79689329 | 4.25599192 | 0.66388957 |

**ASP-390**

| Zero-point correction= | 0.124644 (Hartree/Particle) |
| --- | --- |
| Thermal correction to Energy= | 0.133599 |
| Thermal correction to Enthalpy= | 0.134544 |
| Thermal correction to Gibbs Free Energy= | 0.089282 |
| Sum of electronic and zero-point Energies= | -512.179238 |
| Sum of electronic and thermal Energies= | -512.170283 |
| Sum of electronic and thermal Enthalpies= | -512.169339 |
| Sum of electronic and thermal Free Energies= | -512.214601 |

| C | -0.53525202 | -0.53095277 | 0.36019495 |
| --- | --- | --- | --- |
| H | -0.36277861 | -0.36745784 | 1.43501208 |
| H | -0.43109262 | -1.60425450 | 0.17860182 |
| C | 0.52899897 | 0.23208621 | -0.45083778 |
| H | 0.35073435 | 0.04683345 | -1.52009305 |
| H | 1.90128756 | -0.39728054 | -0.11097486 |
| O | 2.64150632 | 0.35849796 | 0.71069487 |
| H | 2.10228233 | 1.17420199 | 0.86436700 |
| N | 0.55340091 | 1.66061265 | -0.09672069 |
| H | 0.95739246 | 2.19920545 | -0.86034233 |
| H | -0.40076357 | 1.99482873 | 0.02600374 |
| O | 2.24355708 | -1.47783121 | -0.51898890 |
| C | -1.95571580 | -0.09425867 | 0.06632251 |
| O | -2.30380657 | 1.02854230 | -0.23371378 |
| O | -2.83207298 | -1.10737417 | 0.20727757 |
| H | -3.71896390 | -0.73989030 | 0.043108 |

**Gln-386**

| Zero-point correction= | 0.164093 (Hartree/Particle) |
| --- | --- |
| Thermal correction to Energy= | 0.175524 |
| Thermal correction to Enthalpy= | 0.176468 |
| Thermal correction to Gibbs Free Energy= | 0.124991 |
| Sum of electronic and zero-point Energies= | -531.585013 |
| Sum of electronic and thermal Energies= | -531.573582 |
| Sum of electronic and thermal Enthalpies= | -531.572638 |
| Sum of electronic and thermal Free Energies= | -531.624116 |

| H | 3.35851005 | -1.85630545 | -0.45213069 |
| --- | --- | --- | --- |
| C | 2.41491938 | -0.28718388 | -0.01998595 |
| C | 1.17027228 | 0.51561012 | -0.40877196 |
| H | 1.17661205 | 0.61156491 | -1.50378133 |
| C | -0.09538493 | -0.26649237 | 0.00672731 |
| H | -0.13018129 | -0.35561236 | 1.10001695 |
| H | -0.03562381 | -1.28805675 | -0.38629138 |
| C | -1.37957788 | 0.41423489 | -0.47028860 |
| H | -1.45811286 | 0.37731082 | -1.56675208 |
| H | -1.33903279 | 1.47799418 | -0.19713755 |
| C | -2.62758199 | -0.19892347 | 0.16377798 |
| N | -3.79311765 | 0.03660728 | -0.52164382 |
| H | -3.83481413 | 0.68925246 | -1.28888160 |
| H | -4.65871756 | -0.22825004 | -0.07453996 |
| O | -2.60471220 | -0.84697947 | 1.19667384 |
| N | 1.16126332 | 1.85535581 | 0.16996320 |
| H | 1.34594152 | 1.77560262 | 1.16938451 |
| H | 1.93870153 | 2.39479744 | -0.20522120 |
| O | 3.18099641 | 0.00311299 | 0.86856553 |
| O | 2.56919286 | -1.39432244 | -0.78544613 |

**H_2_O**

| Zero-point correction= | 0.021396 (Hartree/Particle) |
| --- | --- |
| Thermal correction to Energy= | 0.024232 |
| Thermal correction to Enthalpy= | 0.025176 |
| Thermal correction to Gibbs Free Energy= | 0.003780 |
| Sum of electronic and zero-point Energies= | -76.399078 |
| Sum of electronic and thermal Energies= | -76.396242 |
| Sum of electronic and thermal Enthalpies= | -76.395298 |
| Sum of electronic and thermal Free Energies= | -76.416694 |

| O | 0.00000000 | -0.00000000 | 0.11081188 |
| --- | --- | --- | --- |
| H | -0.00000000 | 0.78397595 | -0.44324754 |
| H | -0.00000000 | -0.78397595 | -0.44324754 |

**His-Gln-Ser-3,4-Dihydroxybenzaldehyde**

| Zero-point correction= | 0.563204 (Hartree/Particle) |
| --- | --- |
| Thermal correction to Energy= | 0.605498 |
| Thermal correction to Enthalpy= | 0.606442 |
| Thermal correction to Gibbs Free Energy= | 0.476899 |
| Sum of electronic and zero-point Energies= | -1974.945795 |
| Sum of electronic and thermal Energies= | -1974.903501 |
| Sum of electronic and thermal Enthalpies= | -1974.902557 |
| Sum of electronic and thermal Free Energies= | -1975.032100 |

| C | -0.31921964 | -3.61507112 | -1.48481482 |
| --- | --- | --- | --- |
| C | 0.05347151 | -2.32047833 | -1.08313114 |
| C | -0.84410860 | -1.50565677 | -0.40700598 |
| C | -2.14281687 | -1.98304172 | -0.12603333 |
| C | -2.51976488 | -3.26959429 | -0.53755751 |
| C | -1.61250912 | -4.08257188 | -1.20888050 |
| H | 1.04517892 | -1.93368603 | -1.31361419 |
| H | -3.52674202 | -3.61827568 | -0.31645819 |
| H | -1.88544591 | -5.08405747 | -1.53012557 |
| C | 0.64945441 | -4.46466479 | -2.19858692 |
| H | 1.64362158 | -3.98253174 | -2.36730200 |
| O | 0.43965349 | -5.59535338 | -2.59014171 |
| O | -0.45744249 | -0.25886360 | 0.00159509 |
| H | -1.22838848 | 0.30031605 | 0.23971892 |
| C | -5.48183931 | 1.25340020 | 1.08616260 |
| H | -6.43069867 | 1.42750892 | 1.61477091 |
| H | -4.86546623 | 0.59217678 | 1.71677886 |
| C | -5.79321186 | 0.49891788 | -0.24658010 |
| H | -4.82989509 | 0.33542973 | -0.74937946 |
| H | -6.29689959 | -0.89294779 | 0.12673660 |
| N | -6.64714867 | 1.29114812 | -1.10850482 |
| H | -6.94249139 | 0.75951636 | -1.92241338 |
| H | -7.48711199 | 1.58298488 | -0.61561931 |
| O | -5.59607523 | -1.80362714 | 0.54769944 |
| O | -7.62245256 | -1.05065409 | -0.01068658 |
| H | -7.83418950 | -1.95461125 | 0.28420107 |
| O | -4.89740683 | 2.50407107 | 0.88881293 |
| H | -3.98075166 | 2.37664117 | 0.58198424 |
| C | 2.10111900 | 2.96893642 | -1.26538517 |
| C | 4.26514072 | 2.70408494 | -0.89984256 |
| N | 3.03257900 | 2.20525525 | -0.58466163 |
| H | 2.83671583 | 1.40655506 | 0.04096374 |
| H | 5.17519067 | 2.26744365 | -0.50184903 |
| N | 4.19509910 | 3.72969819 | -1.72799687 |
| C | 2.84970014 | 3.90320471 | -1.95603805 |
| C | 0.62306195 | 2.77643268 | -1.14737502 |
| H | 0.13162705 | 3.34657694 | -1.94688362 |
| H | 0.33452025 | 1.72600129 | -1.26806918 |
| C | 0.06316979 | 3.26595599 | 0.22230941 |
| H | 0.45828907 | 2.61543538 | 1.01400207 |
| C | -1.46740936 | 3.11881978 | 0.22614684 |
| N | 0.37765210 | 4.68673685 | 0.43405852 |
| H | 1.21118679 | 4.94110109 | -0.09591495 |
| H | 0.56559820 | 4.88571075 | 1.41301672 |
| O | -2.05100752 | 2.04693653 | 0.31223761 |
| O | -2.11632349 | 4.26093107 | 0.09686721 |
| H | -1.39356096 | 4.95183406 | 0.09690950 |
| C | 6.19514468 | -0.61053696 | -0.46423138 |
| C | 4.78711055 | -1.14711709 | -0.20482109 |
| H | 4.09691659 | -0.45569629 | -0.70719290 |
| H | 4.64011326 | -2.13640807 | -0.65954692 |
| C | 4.46720733 | -1.18953135 | 1.29616100 |
| H | 4.87525170 | -0.28794779 | 1.76859724 |
| H | 4.96454248 | -2.05314298 | 1.75460548 |
| C | 2.94750870 | -1.25480527 | 1.57488235 |
| H | 2.49908100 | -2.01954473 | 0.92794506 |
| O | 6.62112083 | 0.38597522 | 0.10224697 |
| N | 2.30133237 | 0.03763681 | 1.30693373 |
| H | 1.32162810 | -0.10824614 | 1.05461705 |
| H | 2.29996309 | 0.55757090 | 2.18594132 |
| C | 2.71076006 | -1.68952819 | 3.02195462 |
| O | 2.33283656 | -0.95937227 | 3.90840484 |
| O | 3.00117388 | -2.99362827 | 3.21565075 |
| H | 2.85341256 | -3.18087666 | 4.15947618 |
| H | 2.48547677 | 4.67428768 | -2.62734019 |
| N | 6.93882175 | -1.30530169 | -1.37312792 |
| H | 7.83860332 | -0.93039641 | -1.63685015 |
| H | 6.56815606 | -2.08290660 | -1.89548711 |
| O | -2.95046757 | -1.12887044 | 0.54178639 |
| H | -3.86797124 | -1.48057766 | 0.61340793 |

**His403**

| Zero-point correction= | 0.161109 (Hartree/Particle) |
| --- | --- |
| Thermal correction to Energy= | 0.171289 |
| Thermal correction to Enthalpy= | 0.172233 |
| Thermal correction to Gibbs Free Energy= | 0.123871 |
| Sum of electronic and zero-point Energies= | -548.594909 |
| Sum of electronic and thermal Energies= | -548.584729 |
| Sum of electronic and thermal Enthalpies= | -548.583785 |
| Sum of electronic and thermal Free Energies= | -548.632147 |

| C | -1.31525539 | -0.22540261 | 0.38351291 |
| --- | --- | --- | --- |
| C | -3.28260283 | -0.45649241 | -0.62597175 |
| N | -2.03378283 | -1.00502107 | -0.51024951 |
| H | -4.04745757 | -0.88932227 | -1.25999527 |
| N | -3.41421716 | 0.62082068 | 0.11489963 |
| C | -2.19668085 | 0.77127788 | 0.74340138 |
| C | 0.11848098 | -0.48450666 | 0.72308445 |
| H | 0.33035509 | -0.08295595 | 1.72388757 |
| H | 0.33310946 | -1.55869106 | 0.77842723 |
| C | 1.10791604 | 0.14957280 | -0.28256454 |
| H | 0.84932706 | -0.19532403 | -1.29470934 |
| C | 2.54910345 | -0.35730593 | -0.03988418 |
| N | 1.11902831 | 1.61995860 | -0.33357425 |
| H | 1.04453504 | 2.00654788 | 0.60668840 |
| H | 0.32023451 | 1.97038037 | -0.85528621 |
| O | 2.80042576 | -1.47354431 | 0.34009437 |
| O | 3.49029433 | 0.55118333 | -0.32940196 |
| H | 2.99551189 | 1.36641699 | -0.58429294 |
| H | -2.02437233 | 1.58442460 | 1.43972693 |
| H | -1.70997048 | -1.85575446 | -0.94698639 |

**Ser503**

| Zero-point correction= | 0.113830 (Hartree/Particle) |
| --- | --- |
| Thermal correction to Energy= | 0.121422 |
| Thermal correction to Enthalpy= | 0.122366 |
| Thermal correction to Gibbs Free Energy= | 0.081083 |
| Sum of electronic and zero-point Energies= | -398.829377 |
| Sum of electronic and thermal Energies= | -398.821785 |
| Sum of electronic and thermal Enthalpies= | -398.820840 |
| Sum of electronic and thermal Free Energies= | -398.862124 |

| C | 1.19645577 | -0.73608856 | 0.42592344 |
| --- | --- | --- | --- |
| H | 0.98730513 | -0.54214961 | 1.49589261 |
| H | 1.02164041 | -1.79969257 | 0.23486397 |
| C | 0.23772695 | 0.12383858 | -0.43521685 |
| H | 0.41062503 | -0.16120789 | -1.47995791 |
| C | -1.20783483 | -0.24271112 | -0.11442839 |
| N | 0.62870986 | 1.52469944 | -0.27151496 |
| H | 0.25510871 | 2.09722852 | -1.02311962 |
| H | 0.24402487 | 1.89405448 | 0.59527149 |
| O | -1.71615564 | -1.30916688 | -0.36235097 |
| O | -1.87160733 | 0.75150625 | 0.53055854 |
| H | -2.76106044 | 0.40624968 | 0.72293580 |
| O | 2.52957837 | -0.43846120 | 0.09391970 |
| H | 2.54877675 | 0.53136262 | 0.00003112 |

| **No.** | **OSC** | **GenBank ID** | **Species** |
| --- | --- | --- | --- |
| 1 | Hisps | QJQ48095 | *Cloning vector pnnHispS-C1* |
| 2 | A0A2I0A660 | P53416 | [*Apostasia shenzhenica*](https://www.uniprot.org/taxonomy/1088818) |
| 3 | A0A2I0AMU4 | KAK8969165 | [*Apostasia shenzhenica*](https://www.uniprot.org/taxonomy/1088818) |
| 4 | A0A2I0AEN0 | KAK8967435 | [*Apostasia shenzhenica*](https://www.uniprot.org/taxonomy/1088818) |
| 5 | A0A2I0AEL9 | KAK8950878 | [*Apostasia shenzhenica*](https://www.uniprot.org/taxonomy/1088818) |
| 6 | A0A4P8FAP2 | MH992134 | [*Dendrobium officinale*](https://www.uniprot.org/taxonomy/142615) |
| 7 | CAA56277 | X79903 | [*Phalaenopsis sp.*](https://www.uniprot.org/taxonomy/36900) |
| 8 | P53416 | X79904 | [*Phalaenopsis sp.*](https://www.uniprot.org/taxonomy/36900) |
| 9 | CAA56276 | U88077 | *Phalaenopsis sp.* |
| 10 | Q02323 | X60753 | [*Pinus sylvestris*](https://www.uniprot.org/taxonomy/3349) |
| 11 | P48408 | Z46915 | [*Pinus strobus*](https://www.uniprot.org/taxonomy/3348) |
| 12 | P48407 | Z46914 | [*Pinus strobus*](https://www.uniprot.org/taxonomy/3348) |
| 13 | P20077 | X62300 | [*Arachis hypogaea*](https://www.uniprot.org/taxonomy/3818) |
| 14 | P51069 | L00952 | [*Arachis hypogaea*](https://www.uniprot.org/taxonomy/3818) |
| 15 | P20178 | X62299 | [*Arachis hypogaea*](https://www.uniprot.org/taxonomy/3818) |
| 16 | A5AEM3 | AM424663 | [*Vitis vinifera*](https://www.uniprot.org/taxonomy/29760) |
| 17 | P51071 | FN596000 | [*Vitis vinifera*](https://www.uniprot.org/taxonomy/29760) |
| 18 | P51070 | FN597040 | [*Vitis vinifera*](https://www.uniprot.org/taxonomy/29760) |
| 19 | Q9SPW2 | AF128861 | [*Vitis riparia*](https://www.uniprot.org/taxonomy/96939) |
| 20 | A5C9M2 | AM487139 | [*Vitis vinifera*](https://www.uniprot.org/taxonomy/29760) |
| 21 | P28343 | S63225 | [*Vitis vinifera*](https://www.uniprot.org/taxonomy/29760) |
| 22 | A2ICC6 | EF192465 | [*Vitis vinifera*](https://www.uniprot.org/taxonomy/29760) |
| 23 | Q9LKP7 | AF267173 | [*Dianthus monspessulanus*](https://www.uniprot.org/taxonomy/129769) |
| 24 | P06515 | X03710 | [*Antirrhinum majus*](https://www.uniprot.org/taxonomy/4151) |
| 25 | P13114 | M20308 | [*Arabidopsis thaliana*](https://www.uniprot.org/taxonomy/3702) |
| 26 | O04220 | U96661 | [*Chrysosplenium americanum*](https://www.uniprot.org/taxonomy/36749) |
| 27 | Q9AU11 | AF292367 | [*Rubus idaeus*](https://www.uniprot.org/taxonomy/32247) |
| 28 | Q9FUB7 | AF315345 | [*Hypericum androsaemum*](https://www.uniprot.org/taxonomy/140968) |
| 29 | A0A2Z5QL08 | LC133083 | [*Rhododendron dauricum*](https://www.uniprot.org/taxonomy/880079) |

## Table S10 Total 29 genes used for the phylogenetic analysis of figure 4c

## Table S11 Total 15 genes used for the phylogenetic analysis of figure S30a

| **No.** | **OSC** | **GenBank ID** | **Species** |
| --- | --- | --- | --- |
| 1 | OYE1 | X53597 | [*Saccharomyces pastorianus*](https://www.uniprot.org/taxonomy/27292) |
| 2 | Hisps | QJQ48095 | *Cloning vector pnnHispS-C1* |
| 3 | O13437 | X81129 | [*Candida boidinii*](https://www.uniprot.org/taxonomy/5477) |
| 4 | P76469 | U00096 | [*Escherichia coli*](https://www.uniprot.org/taxonomy/83333) |
| 5 | B5R262 | AM933172 | [*Salmonella enteritidis*](https://www.uniprot.org/taxonomy/550537) |
| 6 | Q5ZVK7 | AE017354 | [*Legionella pneumophila subsp. pneumophila*](https://www.uniprot.org/taxonomy/272624) |
| 7 | Q7NRS9 | AE016825 | [*Chromobacterium violaceum*](https://www.uniprot.org/taxonomy/243365) |
| 8 | P0A6L0 | X03224 | [*Escherichia coli*](https://www.uniprot.org/taxonomy/83333) |
| 9 | Q03Q50 | CP000416 | [*Levilactobacillus brevis*](https://www.uniprot.org/taxonomy/387344) |
| 10 | O26909 | AE0006666 | [*Methanothermobacter thermautotrophicus*](https://www.uniprot.org/taxonomy/187420) |
| 11 | Q73A11 | AE017194 | [*Bacillus cereus*](https://www.uniprot.org/taxonomy/222523) |
| 12 | P39121 | X82174 | [*Bacillus subtilis*](https://www.uniprot.org/taxonomy/224308) |
| 13 | Q9KD67 | BA000004 | *Halalkalibacterium halodurans* |
| 14 | Q8Y5R1 | AL591981 | [*Listeria monocytogenes serovar 1/2a*](https://www.uniprot.org/taxonomy/169963) |
| 15 | P39639 | U14003 | *Escherichia coli* |

## Table S12 Total 49 genes used for the phylogenetic analysis of figure S30b

| **No.** | **OSC** | **GenBank ID** | **Species** |
| --- | --- | --- | --- |
| 1 | A0A1M2VPB3 | OJT09444.1 | [*Trametes pubescens*](https://www.uniprot.org/taxonomy/154538) |
| 2 | R7S612 | XP 008044648.1 | [*Trametes versicolor*](https://www.uniprot.org/taxonomy/717944) |
| 3 | VTO73DRAFT | KAL1946175.1 | [*Trametes versicolor*](https://www.uniprot.org/taxonomy/717944) |
| 4 | BDN72DRAFT | TFK58637.1 | *Pluteus cervinus* |
| 5 | FKP32DRAFT | KAI9060051 | *Pluteus cervinus* |
| 6 | B0H18DRAFT | XP 047896736 | *Fomitopsis serialis* |
| 7 | NLJ89_g7335 | KAJ3505596 | *Agrocybe chaxingu* |
| 8 | FRC10_000357 | KAG8743140 | *Ceratobasidium sp.* |
| 9 | VKT23_001495 | KAK7470057 | *Marasmiellus scandens* |
| 10 | VKT23_020501 | KAK7433876 | *Marasmiellus scandens* |
| 11 | unnamed protein product | CAA7258758.1 | *Cyclocybe aegerita]* |
| 12 | VNI00_009047 | KAK7041758.1 | *Paramarasmius palmivorus* |
| 13 | BC827DRAFT_1228401 | KAH9956851 | *Russula dissimulans* |
| 14 | LAESUDRAFT_769211 | XP 040757699.1 | *Laetiporus sulphureus* |
| 15 | L210DRAFT_3670875 | KAF8439829.1 | *Boletus edulis* |
| 16 | EV363DRAFT_1343667 | KAF8127578 | *Boletus edulis* |
| 17 | F5J12DRAFT_728538 | XP 051594635.1 | *Pisolithus orientalis* |
| 18 | M404DRAFT_995627 | KIO10430.1 | *Pisolithus tinctorius* |
| 19 | BKA82DRAFT_995627 | KAI6145492.1 | *Pisolithus tinctorius* |
| 20 | F5148DRAFT_1301247 | KAI9450044.1 | *Russula earlei* |
| 21 | FRC15_003352 | KAG8771551.1 | *Serendipita sp.* |
| 22 | BJV74DRAFT_775506 | KAH9985617.1 | *Russula compacta* |
| 23 | BJV74DRAFT_894984 | KAH9983129.1 | *Russula compacta* |
| 24 | LXA43DRAFT_1136373 | KAI1791811.1 | *Ganoderma leucocontextum* |
| 25 | EDB83DRAFT_596139 | KAH9081916.1 | *Lactarius deliciosus* |
| 26 | EDB86DRAFT_2887796 | KAH9004279.1 | *Lactarius hatsudake* |
| 27 | BJV77DRAFT_345080 | KAH9981924.1 | *Russula vinacea* |
| 28 | DFH94DRAFT_785619 | KAF8464347.1 | *Russula ochroleuca* |
| 29 | PHACADRAFT_118113 | XP 007394286.1 | *Phanerochaete carnosa* |
| 30 | AZE42_06798 | OJA18044.1 | *Rhizopogon vesiculosus* |
| 31 | K503DRAFT_74641 | OAX31557.1 | *Rhizopogon vinicolor* |
| 32 | EUX98_g5521 | THH28675.1 | *Antrodiella citrinella* |
| 33 | EVG20_g8701 | TFY57033.1 | *Dentipellis fragilis* |
| 34 | unnamed protein product | VDC01372.1 | *Peniophora sp.* |
| 35 | M407DRAFT_214897 | KIO23305.1 | *Peniophora sp.* |
| 36 | EWM64_g5261 | TFY78750.1 | *Hericium alpestre* |
| 37 | FOMMEDRAFT_142497 | XP 007269807.1 | *Fomitiporia mediterranea* |
| 38 | EDB85DRAFT_2147336 | KAH9029412.1 | *Lactarius pseudohatsudake* |
| 39 | M408DRAFT_331350 | KIM25253.1 | *Serendipita vermifera* |
| 40 | CONPUDRAFT_159253 | XP007774549.1 | *Coniophora puteana* |
| 41 | PAXINDRAFT_177375 | KIJ11189.1 | *Paxillus involutus* |
| 42 | EDB86DRAFT_3087553 | KAH8980960.1 | *Lactarius hatsudake* |
| 43 | EDB87DRAFT_1672238 | KAH9066404.1 | *Lactarius vividus* |
| 44 | EDB89DRAFT_2110805 | KAH9180786.1 | *Lactarius sanguifluus* |
| 45 | EDB92DRAFT_1962115 | KAH9001043.1 | *Lactarius akahatsu* |
| 46 | FRC03_000833 | KAG8965213.1 | *Tulasnella sp.* |
| 47 | H4582DRAFT_1989651 | KAI9433211.1 | *Lactarius indigo* |
| 48 | H4582DRAFT_1111952 | KAI9432395.1 | *Lactarius indigo* |
| 49 | TRAPUB_14079 | OJT09448.1 | *Trametes pubescens* |

# 4. References

1. T. Kobayashi, M. Ikeda, Y. Okada, Y. Higurashi, S. Okugawa, K Moriya, *Microbiol. Spectr.* **2021**, *9*, e0139921.
2. B. Y. Zhou, J. C. Sun, X. Li, Y. Zhang, B. Luo, N. Jiang, M. C. Liu, E. Criscuolo, *J. Immunol. Res*. **2018**, *2018*, 1.
3. L. Wang, W. Wei, P. Zhou, H. S. Liu, B. L. Yang, L. Feng, R. L. Ge, R. L. Li, F. Tang, *Acta Trop.* **2021**, *222*, 106066.
4. X. F. Hou, Y. J. Song, M. Zhang, W. X. Lan, S. Meng, C. X. Wang, H. X. Pan, C. Y. Cao, G. L. Tang, *Angew. Chem. Int. Edit*. **2018**, *57*, 13475.
5. J. S. Li, Y. L. Du, D. Gu, W. L. Cai, A. Green, S. Ng, A. Leung, A. D. R. Flores, W. J. Zhang, *Org. Lett*. **2020**, *22*, 8204.
6. S. Simm, J. Einloft, O. Mirus, E. Schleiff, *Biol. Res.* **2016**, *49*, 31.
7. B. Rost, C. Sander, *J. Mol. Biol*. **1993**, *232*, 584.
8. A. Schlessinger, G. Yachdav, B. Rost, *Bioinformatics* **2006**, *22*, 891.
9. L. Bartonek, D. Braun, B. Zagrovic, *Proc. Natl. Acad. Sci. USA*. **2020**, *117*, 5907.
10. D. A. Mak, S. Dunn, D. Coombes, C. R. Carere, J. R. Allison, V. Nock, A. O. Hudson, R. C. J. Dobson, *Biochem Mol. Biol. Educ.* **2024**, *52*, 348.
11. S. Bienert, A. Waterhouse, T. A. de Beer, G. Tauriello, G. Studer, L. Bordoli, T. Schwede, *Nucleic Acids Res*. **2017**, *45*, D313.
12. G. Studer, G. Tauriello, S. Bienert, M. Biasini, N. Johner, T. Schwede, *PLoS Comput. Biol*. **2021**, *17*, e1008667.
13. G. Studer, C. Rempfer, A. M. Waterhouse, G. Gumienny, J. Haas, T. Schwede, *Bioinformatics* **2020**, *36*, 1765.
14. C. Camacho, G. Coulouris, V. Avagyan, V. Ma, J. Papadopoulos, K. Bealer, T. L. Madden, *BMC Bioinformatics* **2009**, *10*, 421.
15. M. Steinegger, M. Meier, M. Mirdita, H. Vöhringer, S. J. Haunsberger, J. Söding, *BMC Bioinformatics* **2019**, *20*, 473.
16. C. Zhang, P.L. Freddolino, Y. Zhang, *Nucleic Acids Res*. **2017**, *45*, W291.
17. M. J. Abraham, T. Murtola, R. Schulz, S. Páll, J. C. Smith, B. Hess, E. Lindahl. *SoftwareX* **2015**, *1*, 19.
18. D. Van Der Spoel, E. Lindahl, B. Hess, G. Groenhof, A.E. Mark, H. J. C. Berendsen. *J. Comput. Chem.* **2005**, *26*, 1701.
19. M. J. Frisch, G. W. Trucks, H. B. Schlegel, G. E. Scuseria, M. A. Robb, J. R. Cheeseman, G. Scalmani, V. Barone, G. A. Petersson, H. Nakatsuji, X. Li, M. Caricato, A. V. Marenich, J. Bloino, B. G. Janesko, R. Gomperts, B. Mennucci, H. P. Hratchian, J. V. Ortiz, A. F. Izmaylov, J. L. Sonnenberg, D. W. Young, F. Ding, F. Lipparini, F. Egidi, J. Goings, B. Peng, A. Petrone, T. Henderson, D. Ranasinghe, V. G. Zakrzewski, J. Gao, N. Rega, G. Zheng, W. Liang, M. Hada, M. Ehara, K. Toyota, R. Fukuda, J. Hasegawa, M. Ishida, T. Nakajima, Y. Honda, O. Kitao, H. Nakai, T. Vreven, K. Throssell, J. Montgomery, J. E. Peralta, F. Ogliaro, M. J. Bearpark, J. J. Heyd, E. N. Brothers, K. N. Kudin, V. N. Staroverov, T. A. Keith, R. Kobayashi, J. Normand, K. Raghavachari, A. P. Rendell, J. C. Burant, S. S. Iyengar, J. Tomasi, M. Cossi, J. M. Millam, M. Klene, C. Adamo, R. Cammi, J. W. Ochterski, R. L. Martin, K. Morokuma, O. Farkas, J. B. Foresman, D. J. Fox, *Gaussian 16, Revision A.03*, Gaussian, Inc, CT, Wallingford **2016**.
20. A. D. Becke, *Phys. Rev. A*. **1988**, *38*, 3098.
21. A. D. Becke, *J. Chem. Phys*. **1993**, *98*, 5648.
22. R. Ditchfield, W. J. Hehre, J. A. Pople, *J. Chem. Phys.* **1971**, *54*, 724.
23. P. C. Hariharan, J. A. Pople, *Theoret. Chim. Acta.* **1973**, *28*, 213.
24. K. Fukui, H. Fujimoto, *Frontier Orbitals and Reaction Paths: Selected Papers of Kenichi Fukui*, World Scientific, River Edge, NJ, Singapore **1997.**
